# The Japanese Clinical Practice Guidelines for Management of Sepsis and Septic Shock 2016 (J‐SSCG 2016)

**DOI:** 10.1002/ams2.322

**Published:** 2018-02-05

**Authors:** Osamu Nishida, Hiroshi Ogura, Moritoki Egi, Seitaro Fujishima, Yoshiro Hayashi, Toshiaki Iba, Hitoshi Imaizumi, Shigeaki Inoue, Yasuyuki Kakihana, Joji Kotani, Shigeki Kushimoto, Yoshiki Masuda, Naoyuki Matsuda, Asako Matsushima, Taka‐aki Nakada, Satoshi Nakagawa, Shin Nunomiya, Tomohito Sadahiro, Nobuaki Shime, Tomoaki Yatabe, Yoshitaka Hara, Kei Hayashida, Yutaka Kondo, Yuka Sumi, Hideto Yasuda, Kazuyoshi Aoyama, Takeo Azuhata, Kent Doi, Matsuyuki Doi, Naoyuki Fujimura, Ryota Fuke, Tatsuma Fukuda, Koji Goto, Ryuichi Hasegawa, Satoru Hashimoto, Junji Hatakeyama, Mineji Hayakawa, Toru Hifumi, Naoki Higashibeppu, Katsuki Hirai, Tomoya Hirose, Kentaro Ide, Yasuo Kaizuka, Tomomichi Kan'o, Tatsuya Kawasaki, Hiromitsu Kuroda, Akihisa Matsuda, Shotaro Matsumoto, Masaharu Nagae, Mutsuo Onodera, Tetsu Ohnuma, Kiyohiro Oshima, Nobuyuki Saito, So Sakamoto, Masaaki Sakuraya, Mikio Sasano, Norio Sato, Atsushi Sawamura, Kentaro Shimizu, Kunihiro Shirai, Tetsuhiro Takei, Muneyuki Takeuchi, Kohei Takimoto, Takumi Taniguchi, Hiroomi Tatsumi, Ryosuke Tsuruta, Naoya Yama, Kazuma Yamakawa, Chizuru Yamashita, Kazuto Yamashita, Takeshi Yoshida, Hiroshi Tanaka, Shigeto Oda

**Affiliations:** ^1^ Department of Anesthesiology and Critical Care Medicine Fujita Health University School of Medicine Toyoake Japan; ^2^ Department of Traumatology and Acute Critical Medicine Osaka University Graduate School of Medicine Suita Japan; ^3^ Department of Anesthesiology Kobe University Hospital Kobe Japan; ^4^ Center for General Medicine Education Keio University School of Medicine Tokyo Japan; ^5^ Department of Intensive Care Medicine Kameda Medical Center Kamogawa Japan; ^6^ Department of Emergency and Disaster Medicine Juntendo University Tokyo Japan; ^7^ Depatment of Anesthesiology and Critical Care Medicine Tokyo Medical University School of Medicine Tokyo Japan; ^8^ Depatment of Emergency and Critical Care Medicine Tokai University Hachioji Hospital Hachioji Japan; ^9^ Department of Emergency and Intensive Care Medicine Kagoshima University Graduate School of Medical and Dental Sciences Kagoshima Japan; ^10^ Department of Disaster and Emergency Medicine Kobe University Graduate School of Medicine Kobe Japan; ^11^ Division of Emergency and Critical Care Medicine Tohoku University Graduate School of Medicine Sendai Japan; ^12^ Department of Intensive Care Medicine Sapporo Medical University School of Medicine Sapporo Japan; ^13^ Department of Emergency and Critical Care Medicine Nagoya University Graduate School of Medicine Nagoya Japan; ^14^ Department of Advancing Acute Medicine Nagoya City University Graduate School of Medical Sciences Nagoya Japan; ^15^ Department of Emergency and Critical Care Medicine Chiba University Graduate School of Medicine Chiba Japan; ^16^ Critical Care Medicine National Center for Child Health and Development Tokyo Japan; ^17^ Division of Intensive Care Department of Anesthesiology and Intensive Care Medicine Jichi Medical University School of Medicine Shimotsuke Japan; ^18^ Department of Emergency and Critical Care Medicine Tokyo Women's Medical University Yachiyo Medical Center Tokyo Japan; ^19^ Department of Emergency and Critical Care Medicine Institute of Biomedical and Health Sciences Hiroshima University Higashihiroshima Japan; ^20^ Department of Anesthesiology and Intensive Care Medicine Kochi Medical School Ernakulam Kerala; ^21^ Department of Emergency and Critical Care Medicine School of Medicine Keio University Tokyo Japan; ^22^ Department of Surgery Beth Israel Deaconess Medical Center Harvard Medical School Boston MA USA; ^23^ Healthcare New Frontier Promotion Headquarters Office Kanagawa Prefectural Government Yokohama Japan; ^24^ Department of Anesthesia and Pain Medicine The Hospital for Sick Children Toronto Ontario Canada; ^25^ Department of Anesthesia Faculty of Medicine University of Toronto Toronto Ontario Canada; ^26^ Division of Emergency and Critical Care Medicine Department of Acute Medicine Nihon University School of Medicine Tokyo Japan; ^27^ Department of Acute Medicine The University of Tokyo Tokyo Japan; ^28^ Department of Anesthesiology and Intensive Care Hamamatsu University School of Medicine Hamamatsu Japan; ^29^ Department of Anesthesiology St. Mary's Hospital Fukuoka Japan; ^30^ Division of Infectious Diseases and Infection Control Tohoku Medical and Pharmaceutical University Hospital Sendai Japan; ^31^ Department of Emergency Medicine Beth Israel Deaconess Medical Center Harvard Medical School Boston MA USA; ^32^ Department of Anesthesiology and Intensive Care Faculty of Medicine Oita University Oita Japan; ^33^ Department of Emergency and Intensive Care Medicine Mito Clinical Education and Training Center Tsukuba University Hospital Mito Kyodo General Hospital Mito Japan; ^34^ Department of Anesthesiology and Intensive Care Medicine Kyoto Prefectural University of Medicine Kyoto Japan; ^35^ Department of Intensive Care Medicine Yokohama City Minato Red Cross Hospital Yokohama Japan; ^36^ Emergency and Critical Care Center Hokkaido University Hospital Sapporo Japan; ^37^ Kagawa University Hospital Emergency Medical Center Kita Japan; ^38^ Department of Anesthesia and Critical Care Kobe City Medical Center General Hospital Kobe City Hospital Organization Kobe Japan; ^39^ Department of Pediatrics Kumamoto Red Cross Hospital Kumamoto Japan; ^40^ Emergency and Critical Care Medical Center Osaka Police Hospital Osaka Japan; ^41^ Department of Emergency and ICU Steel Memorial Yawata Hospital Kitakyushu Japan; ^42^ Department of Emergency and Critical Care Medicine Kitasato University Tokyo Japan; ^43^ Department of Pediatric Critical Care Shizuoka Children's Hospital Shizuoka Japan; ^44^ Department of Anesthesia Obihiro Kosei Hospital Obihiro Japan; ^45^ Department of Surgery Nippon Medical School Chiba Hokusoh Hospital Inzai Japan; ^46^ Department of Emergency and Critical Care Medicine Tokushima University Hospital Tokushima Japan; ^47^ Department of Epidemiology University of North Carolina Gillings School of Global Public Health Chapel Hill NC USA; ^48^ Department of Emergency Medicine Gunma University Graduate School of Medicine Maebashi Japan; ^49^ Shock and Trauma Center Nippon Medical School Chiba Hokusoh Hospital Inzai Japan; ^50^ Department of Emergency and Critical Care Medicine Juntendo University Nerima Hospital Tokyo Japan; ^51^ Department of Emergency and Intensive Care Medicine JA Hiroshima General Hospital Hiroshima Japan; ^52^ Department of Intensive Care Medicine Nakagami Hospital Okinawa Japan; ^53^ Department of Aeromedical Services for Emergency and Trauma Care Ehime University Graduate School of Medicine Matsuyama Japan; ^54^ Division of Acute and Critical Care Medicine Department of Anesthesiology and Critical Care Medicine Hokkaido University Graduate School of Medicine Sapporo Japan; ^55^ Department of Traumatology and Acute Critical Medicine Osaka University Osaka Japan; ^56^ Department of Emergency and Critical Care Medicine Hyogo College of Medicine Nishinomiya Japan; ^57^ Department of Emergency and Critical Care Medicine Yokohama City Minato Red Cross Hospital Yokohama Japan; ^58^ Department of Intensive Care Medicine Osaka Women's and Children's Hospital Osaka Japan; ^59^ Department of Anesthesiology and Intensive Care Medicine Kanazawa University Kanazawa Japan; ^60^ Advanced Medical Emergency and Critical Care Center Yamaguchi University Hospital Ube Japan; ^61^ Department of Diagnostic Radiology Sapporo Medical University School of Medicine Sapporo Japan; ^62^ Division of Trauma and Surgical Critical Care Osaka General Medical Center Osaka Japan; ^63^ Department of Healthcare Economics and Quality Management Graduate School of Medicine Kyoto University Kyoto Japan; ^64^ Intensive Care Unit Osaka University Hospital Suita Japan; ^65^ Department of Emergency and Disaster Medicine Juntendo University Graduate School of Medicine Tokyo Japan

**Keywords:** Sepsis, septic shock, guidelines, evidence‐based medicine, systematic review, Medical Information Network Distribution Service (Minds)

## Abstract

**Background and Purpose:**

The Japanese Clinical Practice Guidelines for Management of Sepsis and Septic Shock 2016 (J‐SSCG 2016), a Japanese‐specific set of clinical practice guidelines for sepsis and septic shock created jointly by the Japanese Society of Intensive Care Medicine and the Japanese Association for Acute Medicine, was first released in February 2017 in Japanese. An English‐language version of these guidelines was created based on the contents of the original Japanese‐language version.

**Methods:**

Members of the Japanese Society of Intensive Care Medicine and the Japanese Association for Acute Medicine were selected and organized into 19 committee members and 52 working group members. The guidelines were prepared in accordance with the Medical Information Network Distribution Service (Minds) creation procedures. The Academic Guidelines Promotion Team was organized to oversee and provide academic support to the respective activities allocated to each Guideline Creation Team. To improve quality assurance and workflow transparency, a mutual peer review system was established, and discussions within each team were open to the public. Public comments were collected once after the initial formulation of a clinical question (CQ), and twice during the review of the final draft. Recommendations were determined to have been adopted after obtaining support from a two‐thirds (>66.6%) majority vote of each of the 19 committee members.

**Results:**

A total of 87 CQs were selected among 19 clinical areas, including pediatric topics and several other important areas not covered in the first edition of the Japanese guidelines (J‐SSCG 2012). The approval rate obtained through committee voting, in addition to ratings of the strengths of the recommendation and its supporting evidence were also added to each recommendation statement. We conducted meta‐analyses for 29 CQs. Thirty seven CQs contained recommendations in the form of an expert consensus due to insufficient evidence. No recommendations were provided for 5 CQs.

**Conclusions:**

Based on the evidence gathered, we were able to formulate Japanese‐specific clinical practice guidelines that are tailored to the Japanese context in a highly transparent manner. These guidelines can easily be used not only by specialists, but also by non‐specialists, general clinicians, nurses, pharmacists, clinical engineers, and other healthcare professionals.

## INTRODUCTION

Sepsis is a serious disease affecting all age groups, and the societal significance of developing high‐quality guidelines is very high. Japanese guidelines formulated in consideration of the clinical environment in Japan were announced by the Japanese Society of Intensive Care Medicine in 2012.^1^, ^2^ During the 2016 revision, a joint committee was organized in conjunction with the Japanese Association for Acute Medicine. Rather than simply releasing another revised edition, we strove to create high‐quality guidelines that are still easy to understand for general practitioners in order to encourage their spread throughout the target medical community. These guidelines are the English‐language version prepared in reference to The Japanese Clinical Practice Guidelines for Management of Sepsis and Septic Shock 2016 (J‐SSCG 2016)^3^, ^4^ originally published in Japanese in February 2017. The Japanese version of the J‐SSCG 2016^3^, ^4^ is a large‐scale guideline containing 232 pages of main body content and 157 pages of appendix materials. While preparing the English version, the content of the Japanese version was digested and translated into English. It should also be noted that these guidelines were originally prepared while taking medical conditions in Japan into consideration and are wholly independent of “Surviving Sepsis Campaign: International Guidelines for Management of Severe Sepsis and Septic Shock: 2016 (SSCG 2016)”. For this reason, these guidelines contain some instances in which the recommendations offered differ from those offered for similar clinical questions (CQs) in the SSCG 2016, or that address topics not covered in the SSCG 2016. New topics not covered in the first edition of the J‐SSCG^1^, ^2^ include controlling of the origin of infection, blood transfusion preparations, management of analgesia, sedation and delirium, acute kidney injury, body temperature regulation, venous thromboembolism countermeasures, intensive care unit (ICU)‐acquired weakness, and post‐intensive care syndrome. Moreover, there are few pediatric ICUs in Japan, and as healthcare professionals handling adult patients will inevitably need to treat pediatric sepsis cases as well, new CQs related to pediatric sepsis patients were also added to this edition. As a result, these guidelines ultimately comprised a large‐scale reference material covering a total of 19 clinical areas and 87 CQs. However, therapy administration to patients in the prone position during respiratory management has been recently addressed by the Japanese Acute Respiratory Distress Syndrome (ARDS) Clinical Practice Guidelines. As such, some CQs in the J‐SSCG 2016 avoid more specialized discussion of some topics related to this area and some overlapping topics are not covered. To improve quality assurance and workflow transparency, a mutual peer review system was established, and discussions within each team were open to the public. Public comments were collected once after the initial formulation of a CQ, and twice during the review of the final draft. These guidelines were published simultaneously in both the Journal of Intensive Care, the English‐language journal of the Japanese Society of Intensive Care Medicine, and in Acute Medicine and Surgery, the English‐language journal of the Japanese Association for Acute Medicine.

## OVERVIEW AND BASIC PRINCIPLES OF THESE GUIDELINES

1 *Title:* These guidelines were titled, “The Japanese Clinical Practice Guidelines for Management of Sepsis and Septic Shock 2016”, which is abbreviated to “J‐SSCG2016”, in accordance with international versions (SSCG2016).
2 *Purpose:* The purpose of these guidelines is to support the capacity of healthcare professionals to appropriately judge patient condition in the treatment of sepsis and septic shock in order to improve prognosis.
3 *Target patient population:* These guidelines target pediatric to adult patients presenting with confirmed or suspected sepsis or septic shock. These patients may include not only those in ICUs, but also general wards or emergency outpatients. However, although physicians may understand the diagnosis and treatment of some cases, sepsis cases require advanced systemic management. As such, we emphasize that prompt transfer of patients presenting with confirmed or suspected sepsis to the ICU is desirable as circumstances permit.
4 *Target audience (anticipated users of these guidelines):* These guidelines are meant for healthcare professionals such as specialists, non‐specialists, general practitioners, nurses, pharmacists, and clinical engineering technicians who perform or contribute to sepsis treatment.
5 *Usage warnings:* These guidelines were designed to improve overall treatment outcomes. Although they are non‐binding, their societal impact is great. These guidelines are not laws, and if other experts in this field achieve superior treatment results through other methods, adhering to these guidelines in their entirety is not necessary in such instances. Accordingly, the contents of these guidelines were designed to be easy for general practitioners to understand, and highly specialized topics were avoided. Clear recommendations could not be offered for some CQs. Pathogens and infections capable of causing sepsis are diverse, and the disease can appear in varying degrees of severity. Sepsis cannot be managed effectively by simply applying a standardized algorithm or recommendation. Although it is important to abide by treatment guidelines, healthcare professionals using these guidelines are encouraged to do so as necessary based on the circumstances of each case, and to avoid becoming overly concerned with adherence. The Guideline Creation Committee does not allow these guidelines to be used or admitted as evidence in court.
6 *Organizational structure:* Members of the Japanese Society of Intensive Care Medicine and the Japanese Association for Acute Medicine were selected and organized into 19 committee members and 52 working group members. The Academic Guidelines Promotion Team was organized to oversee guideline creation from a neutral position in order to integrate each subject area into a single unified guideline. The Academic Guidelines Promotion Team audits the activities of each Guideline Creation Team to ensure uniformity throughout the guidelines, and also creates academic materials and provides support to improve systematic reviews.  In view of the broad range of advanced medical knowledge required to understand the complexity and pathophysiology of sepsis, it was also decided that members of patients’ families and patient advocates would be withheld in a committee holding voting rights. Although a separate organization, the Guideline Creation Committee occasionally acted based on the guidance and support of the Medical Information Network Distribution Service (Minds).
7 *Quality and transparency assurance:* In addition to establishing the Academic Guidelines Promotion Team, the following efforts were made to ensure quality and transparency. 
  (a) Collaboration with Minds and workshop activities Occasional guidance was received from Minds during the process of formulating these guidelines. In addition, external lecturers and librarians were invited to participate in a seminar on “Literature Acquisition Techniques for Systematic Reviews” we held independently.
  (b) Peer review Activities were performed for various work processes while mutual peer review was conducted by team members across the region. Work products from each group were repeatedly edited and revised, with each revised draft being discussed by the Guideline Creation Committee.
  (c) Multiple rounds of public comments CQs underwent multiple rounds of public comments generally from registered contributors: once after the initial formulation of a CQ, and twice during the review of the final draft. During finalization, public commenters were requested to disclose any conflicts of interest. Opinions regarding draft CQs were also solicited over the internet.
  (d) Transparency Although it is difficult to create guidelines that will be accepted universally, improving visibility and transparency in the development process is crucial. Members of each team created an official mailing list (ML) and discussions among team members were held using these MLs as much as possible. Core members and members of the Academic Guidelines Promotion Team joined the MLs established by each team as read‐only members. Through these measures, we aimed to increase the transparency of team discussions, and by implementing the appropriate interventions, we were able to coordinate the directions taken by each team and achieve consistency throughout the entirety of the guidelines.
  (e) Vote anonymization Votes were tallied after all 19 Committee members had participated, and the rate of agreement achieved was mentioned in each recommendation. To avoid confounding from academic conflicts of interest (COIs) of committee members, committee votes concerning draft recommendations were anonymized.
  (f) Disclosure of COIs and members’ roles Financial and academic COIs as well as the role(s) of each committee member are disclosed in the “Appendix”. Financial COIs were disclosed in accordance with the standards used by the Japanese Association of Medical Sciences since 2013 through 2016.
8 *Funding*: These guidelines were prepared with financial support from the Japan Society of Intensive Care Medicine and the Japanese Association for Acute Medicine. No member of the Guideline Creation Committee received any form of financial compensation during the preparation of these guidelines. The views and interests of these societies as well as Minds were not reflected in the preparation of the guideline's recommendations.
9 *Guideline dissemination strategy*: The Japanese version of these guidelines is open access. In addition, to promote ease of use, the digest version of the guidelines booklet as well as apps viewable on smartphones and tablet devices are available for purchase at the affordable price of 2,500 JPY. We will strive to make these guidelines available at various academic meetings and seminars and also monitor activities related to sepsis practice as well as the spread of these guidelines throughout the target medical community.
10 *Planned revisions*: These guidelines are scheduled to undergo revision every 4 years. The next revision will occur in 2020. Should important new information warranting revision be obtained beforehand, partial revision will be considered.

## THE PROCESS OF MAKING RECOMMENDATIONS IN THE JAPANESE CLINICAL PRACTICE GUIDELINES FOR MANAGEMENT OF SEPSIS AND SEPTIC SHOCK 2016

Each recommendation in the Japanese Clinical Practice Guidelines for Management of Sepsis and Septic Shock 2016 went through four steps in its formulation: (i) clinical question (CQ) development, (ii) systematic review, (iii) evaluation of the quality of evidence (QoE), and (iv) determination of the recommendation. In principle, this method proceeded in accordance with the Minds 2014 system(http://minds4.jcqhc.or.jp/minds/guideline/handbook2014.html).

When formulating the recommendations, teams involved in the management of pediatric patients, in addition to adult patients were assembled, and each team developed CQs, conducted systematic reviews, evaluated the QoE, and drafted a recommendation in one of the following areas: “Definition and diagnosis of sepsis”, “Diagnosis of infection”, “Antimicrobial therapy”, “Imaging diagnoses”, “Source control”, “Initial resuscitation and vasoactive medications”, “Respiratory management”, “Nutrition”, “Corticosteroid therapy”, “Disseminated Intravascular Coagulation (DIC) management”, “Acute Kidney Injury (AKI)/Blood purification and renal replacement therapy”, “Immunoglobulins”, “Analgesia/Sedation/Delirium”, “Post Intensive Care Syndrome (PICS)/Intensive Care Unit‐Acquired Weakness (ICU‐AW) ”, “Body temperature regulation”, “Glucose control”, “Blood products”, and “Venous thromboembolism prophylaxis”.

In three areas (Respiratory management, Nutrition, and Analgesia/Sedation/Delirium), recommendations were formulated based on the existing recently published clinical guidelines in collaboration with members of the clinical guideline committees of related local academic societies.

## STRENGTH OF RECOMMENDATIONS

The recommendations were made based on four factors: QoE, the balance between benefit and harm, patients’ values and preferences, and the costs and resources involved in carrying out the intervention. The strength of the recommendations was defined based on the Minds 2014 system. The strength of the recommendations is classified into one of four categories: recommend, suggest, recommend against, or recommend against (table 1).

**Table 1:** Strength of recomendations

Strength of Recommendation	Recommend (1)	Suggest (2)	Suggest against (2)	Recommend against (1)
Content of recommendation	Strong recommendation in support of an intervention	(Weak) Suggestion in support of an intervention under certain conditions	(Weak) Suggestion against an intervention under certain conditions	Strong recommendation against an intervention
Wording of recommendation	We recommend—[intervention]	We suggest—[intervention]	We suggest against—[intervention]	—We recommend against [intervention]

Following the formulation of statements through discussion in each group and deliberation among all committee members during face‐to‐face meetings at which the groups presented their draft statements, all committee members voted to indicate their agreement or disagreement with the statement, or abstention. Acceptance of a statement required votes from 66.6% of the 19 committee members. The accepted recommendations were edited and finalized by the committee. Voters could provide feedback for consideration in revising statements that did not receive consensus in up to two rounds of voting.

As a result, the two CQs that were not accepted after two rounds of voting are presented as expert consensuses.

## EXPERT CONSENSUS PRESENTATION

An expert consensus is presented for CQs for which no systematic review or randomized clinical trial could be identified after a comprehensive literature search, or when the recommendation statement was unable to be accepted by the committee.

Recommendations are presented as an expert consensus only when they are feasible clinical solutions (clinically important aspects that cannot be verified via intervention trials as they are physiologically common phenomena) after consideration of the appropriate physiological or pathophysiological circumstances. When it was not possible to make recommendations as an expert consensus, or if a consensus could not be reached, it was stated that no recommendation for that CQ could be offered with the related discussions.

## CQ1: SEPSIS: DEFINITION AND DIAGNOSIS

### Introduction

According to the Third International Consensus Definitions for Sepsis and Septic Shock (Sepsis‐3),^5^, ^6^, ^7^ sepsis is defined as “life‐threatening organ dysfunction caused by a dysregulated host response to infection.” The clinical criteria of sepsis are suspected or documented infection and an acute increase in the Sequential Organ Failure Assessment (SOFA) score of 2 points or more. Septic shock is defined as a subset of sepsis in which the underlying circulatory and cellular/metabolic abnormalities are profound enough to substantially increase mortality. Septic shock can be clinically identified by a vasopressor requirement to maintain a mean arterial pressure of 65 mmHg or higher and a serum lactate level greater than 2 mmol/L (18 mg/dL) despite adequate volume resuscitation.

In out‐of‐hospital, emergency department, or general hospital ward settings, adult patients with suspected infection can be rapidly identified as being more likely to have poor outcomes typical of sepsis if they have at least 2 of the following clinical criteria that together constitute the quick SOFA (qSOFA): a respiratory rate of 22 breaths/min or higher, altered consciousness, and a systolic blood pressure of 100 mmHg or less.^5^, ^6^, ^7^ The qSOFA criteria can be used to prompt clinicians to further investigate for organ dysfunction, to initiate or escalate therapy as appropriate, and to consider referral for critical care. Ultimately, an acute increase in the SOFA score of 2 or more points constitutes a confirmation of the diagnosis of sepsis. Daily routine sepsis screening is recommended to support the early diagnosis and treatment of sepsis.

Various biomarkers believed to be useful in diagnosing sepsis have been reported; in the Sepsis‐2 (2003),^8^ leukocyte count (>12,000/μL or <4,000/μL or >10% immature forms), c‐reactive protein level (CRP, >reference value + 2 standard deviation (SD)), and procalcitonin level (PCT, >reference value + 2 SD) were listed as inflammatory biomarkers. CRP and PCT are also commonly used by physicians in Japan. In addition to this, Japanese‐developed presepsin (P‐SEP, sCD14‐ST) came under the coverage of the National Health Insurance in January 2014. Although the test for interleukin‐6 (IL‐6) is not yet covered, a kit for clinical use has been developed and is currently in use by some medical facilities as part of the management of sepsis. This guideline covers CRP, PCT, PSEP, and IL‐6 based on the background described above.

### CQ1‐1: Can we use procalcitonin (PCT), presepsin (P‐SEP,sCD14‐ST), and interleukin‐6 (IL‐6) for the diagnosis of sepsis?

#### Answer (recommendations)

1. (P‐SEP: 2B, PCT: 2C) We suggest the measurement of P‐SEP or PCT levels as an adjunct to the diagnosis of infection when sepsis is suspected in critically‐ill patients such as those in intensive care units (rate of agreement: 89.4%). We do not recommend the routine measurement of IL‐6 levels as an adjunct to the diagnosis of infection in such patients (2C) (rate of agreement: 89.4%).
2. We suggest against the routine measurement of P‐SEP, PCT, or IL‐6 levels as an adjunct to the diagnosis of infection when sepsis is suspected in non‐critically ill patients such as those in emergency rooms or general wards (P‐SEP: 2C, PCT: 2D, IL‐6: 2D) (rate of agreement: 94.7%).

#### Rationale

This clinical question (CQ) offers recommendations regarding the validity of the three biomarkers, PCT, P‐SEP, and IL‐6 to support the diagnosis of sepsis in two clinical settings: (i) settings with critically‐ill patients, such as in ICUs, where infection is suspected but difficult to confirm, and (ii) settings in which infection is suspected but patients are not critically ill such as the emergency room or general ward. The clinical utility of each marker was assessed individually in these two settings.

Hierarchical summary receiver operating characteristic (ROC) analysis was used during meta‐analysis (data integration) of the diagnostic test accuracy for each marker, and the assessment of the quality of experience (QoE) and the recommended settings were calculated based on the estimated number of patients presenting as true positives, false positives, or false negatives determined by the “diagnostic Grading of Recommendations Assessment, Development and Evaluation (GRADE) system,” and the benefit‐risk balance was assessed based on a pre‐examination probability of 40%. We adopted CRP, a widely used biomarker in clinical practice, as a control. Representative meta‐analyses of PCT,^9^ P‐SEP,^10^ IL‐6,^11^ and CRP^12^ were selected.

In the settings where most patients were critically ill, the benefits were evaluated to outweigh risks regarding the measurement of P‐SEP or PCT, but not of IL‐6 levels. As a result, we recommend the measurement of P‐SEP or PCT levels as supplementary tests in the diagnosis of infection in critically‐ill patients when sepsis is suspected. In settings where most patients are not critically ill, significant benefit has not been established regarding the measurement of P‐SEP, PCT, or IL‐6 levels. Thus, we do not recommend the routine measurement of any of these biomarkers as a supplementary test in the diagnosis of infection in non‐critically ill patients even when sepsis is suspected.

Access to tests for these biomarkers is variable among hospitals or facilities. Currently, only a limited number of hospitals or facilities in Japan are capable of measuring P‐SEP and IL‐6 values as part of routine examinations. Moreover, even in hospitals or facilities capable of performing these measurements, these tests are performed in central laboratories, and may not be as useful as point‐of‐care‐testing (POCT).

## CQ2: DIAGNOSIS OF INFECTION

### Introduction

Identifying the source of infection is important for the diagnosis of sepsis or septic shock. It is necessary to narrow down the potential foci of infection as quickly as possible based on the patient's medical history, physical examination, imaging examinations, and other records, as well as to properly collect specimens from the suspected foci and perform a blood culture examination. The blood culture is the most important test in the management of sepsis, and the clinical significance of identifying the pathogenic microorganisms causing bacteremia is substantial. Treatment optimization including de‐escalation can be achieved with the aid of the results of culture and antimicrobial susceptibility tests of blood samples or other specimens. On the other hand, contamination is associated with unnecessary treatment and increases in medical costs, which can be an impediment to treatment optimization. Therefore, it is critical for all clinicians involved in the management of sepsis to understand when and how to collect culture specimens.

In general, sepsis is to be suspected, and blood culture examinations are to be performed proactively in patients presenting with suspected symptoms of bacteremia (fever, chills, hypotension, tachypnea, etc.), hypothermia and hypotension of unknown cause, altered consciousness (particularly in elderly patients), unexplained increase or decrease in leukocyte count, unexplained metabolic acidosis or respiratory failure, acute renal damage, or acute liver damage of unknown origin in immunocompromised patients.^13^

Disinfectants used on the skin include chlorhexidine gluconate, povidone iodine, 70% alcohol, and others, but their effectiveness in suppressing potential contaminants has not been established. According to a small scale meta‐analysis^14^ comparing alcohol‐containing chlorhexidine gluconate with povidone iodine, chlorhexidine gluconate was shown to decrease contamination, but some of these studies used 2% chlorhexidine gluconate, which is not used in Japan. Povidone iodine requires ~2 min to take effect, and there is the concern that medical staff tasked with collecting specimens may not wait for a sufficient amount of time.^15^ In contrast, alcohol‐containing chlorhexidine gluconate has both immediate and sustained effects. Ensuring an aseptic procedure is crucial.^16^

Because the quantity of bacteria in blood during sepsis is very small, the sensitivity of blood cultures depends on the amount of blood collected.^17^ It has been reported that sensitivity increases by 10% if the quantity of the blood sample increases from 40 to 60 mL,^18^ but this increment in sensitivity gets smaller as more blood is collected. In addition, as the volume of blood collected increases, the risk of iatrogenic anemia becomes a concern. In general, a blood sample volume of 20–30 mL per set is recommended.^15^

Cockerill *et al*. examined 163 patients presenting with bloodstream infections (excluding infective endocarditis) and collected more than two sets of blood cultures within 24 h; the test sensitivity was 65.1% for the first set, 80.4% for the first and second sets, and 95.7% for three sets.^17^ In addition, Lee *et al*. examined 629 patients whose blood culture tests yielded positive results after three or more sets were collected within 24 h; the sensitivity was 73.1% for the first set, 89.7% for the first and second sets, and 98.2% with three sets.^19^ Based on the above data, we conclude that a minimum of two sets (three sets, if possible) should be collected within 24 h. A further increase in test sensitivity should not be expected if the number of sets collected exceeds three. If infective endocarditis is suspected, three sets must be collected within 24 h.^20^

In cases where catheter‐related bloodstream infections are suspected (signs of local infection, long‐term indwelling catheter, frequent use of stopcocks, catheter occlusion, thrombus formation, etc.), one set of blood culture should be aspirated from the catheter lumen. If the test results from the catheter and peripheral vessels are positive for the same pathogen, and the former returns positive earlier by >2 h, the catheter is considered to be the source of infection.^21^, ^22^ Many bacterial species from resident cutaneous flora can cause contamination. Examples are coagulase‐negative staphylococci, *Bacillus*,* Corynebacterium*, and *Propionibacterium*. If test results are positive for these bacteria after 48–72 h for only from one sample bottle or set, contamination should be suspected.^15^

Although there is no scientific basis for collecting specimens from possible foci of infection prior to administering antimicrobial agents, this practice is recommended in many guidelines.^23^, ^24^, ^25^, ^26^, ^27^ De‐escalation based on the culture results is expected to reduce costs and adverse events, and prevent the emergence of resistant bacteria without increasing the harm to patients. Therefore, it is reasonable to collect specimens from suspected foci of infection prior to the administration of antimicrobial agents, so as not to decrease detection sensitivity.

Sputum can be contaminated together with the resident flora of the upper respiratory tract. In severe cases of pneumonia, sputum cultures (specimens collected by intratracheal aspiration if tracheal intubation is performed), as well as urinary antigen testing for *Legionella pneumophila* and *Streptococcus pneumoniae*, may be performed in addition to blood culture.^24^ When switching to broad‐spectrum antimicrobials in the management of hospital‐acquired pneumonia or ventilator‐associated pneumonia, specimens should be taken from the lower respiratory tract before switching to different antimicrobials.^25^

Urine specimens should also be taken before administering antimicrobials. When interpreting test results, it is necessary to differentiate urinary tract infection from asymptomatic bacteriuria.^26^

If a lumbar puncture is required, can be performed quickly, cerebrospinal fluid should be collected prior to antimicrobial administration. However, bacterial meningitis requires urgent treatment, and if lumbar puncture cannot be performed for some reason, administration of antimicrobials should be given priority.^27^ Even in such cases, blood cultures should be collected prior to the administration of antimicrobial agents.^28^

The practice of referring to Gram stain findings when selecting empiric antimicrobial agents has been widely adopted in Japan, and this practice is considered to have some validity from the pathophysiological standpoint as well. However, in general, the sensitivity and specificity of Gram stain findings are greatly affected by the quality of the specimen (i.e., presence or absence of contamination) as well as the level of experience of the assessor. As such, when referring to Gram stain results in antimicrobial agent selection, one should keep these factors in mind.

### CQ 2‐1: When and how should a blood culture be taken?

#### Answer (opinion)

A blood culture should be taken prior to antimicrobial administration in patients with sepsis or septic shock (expert consensus/no evidence) (rate of agreement: 100%).

#### Rationale

No randomized controlled trial (RCT) was found to conform to the Patient, Intervention, Comparison, Outcome (PICO) process. The diagnosis of bacteremia fortifies the diagnostic accuracy of infection. Identifying the causative microorganisms and subsequently performing antimicrobial susceptibility tests lead to treatment optimization. However, as the volume of blood collected increases so does the risk of iatrogenic anemia, but the benefits of taking a blood culture are considered to outweigh the potential risks in all cases of sepsis. Also, because the detection sensitivity is not expected to increase beyond three culture sets, oversampling should be avoided.

### CQ 2‐2: When and how should culture specimens other than blood be collected?

#### Answer (opinion)

In patients presenting with sepsis or septic shock, various culture specimens other than blood may be collected as necessary prior to administering antimicrobials (expert consensus/no evidence) (rate of agreement: 100%).

#### Rationale

No RCT conforming to the PICO process has been identified. Substantial benefits can be obtained from the culture results of suspected site of infection. Such benefits are thought to outweigh the potential risks in any case of sepsis. However, as there are risks associated with this procedure, specimens should not be collected unless the collection site is suspected to be a focus of infection.

### CQ 2‐3: Is Gram staining useful in the selection of antimicrobial agents before obtaining culture results?

#### Answer (opinion)

When selecting antimicrobial agents for empiric treatment, Gram staining may be considered (expert consensus/no evidence) (rate of agreement: 100%).

#### Rationale

No RCT conforming to the PICO process has been identified; thus the risk‐benefit balance is unknown. However, favorable specificity has been reported in community‐acquired pneumonia, urinary tract infections, and bacterial meningitis. Considering the simplicity, rapidity, and low costs associated with this technique, the benefits may sufficiently outweigh any potential risks.

## CQ3: IMAGING DIAGNOSES

### Introduction

In sepsis, rapid therapeutic intervention to treat the focus of infection is recommended.^29^, ^30^ Therefore, detecting the sites of infection is critical. Detection of the sites of infection based on physiological findings and culture tests from each suspected region is essential for determining the intervention. Thus, the following clinical question (CQ) concerning imaging diagnoses is presented.

First, the question of whether imaging diagnoses should be performed is addressed. There have been no studies conducted to date that examine whether any difference in prognosis can be obtained as a result of performing diagnostic imaging, and such a study is not expected to be conducted in the future. However, in the clinical setting, some types of imaging examinations are routinely performed depending on the disease and the suspected sites of infection. The following paragraphs offer an explanation for specific diagnostic imaging techniques relevant to each organ and their associated diseases.

In bacterial meningitis, it is generally accepted not to perform a routine cranial computed tomography (CT) scan prior to lumbar puncture. However, performing brain CT examinations is recommended in patients presenting with altered consciousness, neurological symptoms, convulsions, and in patients over 60 years of age.^31^ In addition, magnetic resonance imaging (MRI) yields more informative results than CT images and are excellent for evaluating the spread of lesions. Fluid attenuation inversion recovery (FLAIR) images are also useful for identifying sites of inflammation.^32^

Diagnoses based on transesophageal echocardiography following transthoracic echocardiography is recommended in cases where infective endocarditis is suspected, particularly those involving prosthetic valve replacement, when the clinical criteria indicate a strong possibility of infective endocarditis, or in high‐risk cases accompanied by complications such as annular abscess.^33^ Performing a contrast‐enhanced CT scan is necessary to determine the drainage range for deep cervical abscesses and descending mediastinitis. If symptoms do not improve, a second contrast‐enhanced CT scan should be performed to identify the spread of the abscess, after which prompt source control should be taken.^34^ Chest x‐rays are important when diagnosing respiratory infections. Pulmonary CT scans can also be used to diagnose pleural effusion, atelectasis, and tumorous lesions that are difficult to distinguish via chest x‐ray, and the use of this technique is recommended as an auxiliary diagnostic method under the Acute respiratory distress syndrome (ARDS) diagnostic criteria (Berlin definition).^35^

For the diagnosis of intraperitoneal infections, abdominal ultrasonography and abdominal CT examinations are useful for identifying the origin of infection and are recommended in line with relevant guidelines and treatment policies.^36^ Diagnostic imaging using ultrasound is recommended in cases of acute suppurative cholangitis, and reaching a definitive diagnosis via CT or magnetic resonance cholangiopancreatography (MRCP) is important when local complications such as perforation or abscess formation are suspected.^37^ In cases of sepsis caused by urinary tract infection (caused by a kidney stone or indwelling catheter) or infection of the male genitalia, the source of infection can be identified through abdominal ultrasonography or abdominal CT examination.^38^ Although kidney, ureter, and bladder simple X‐ray image (KUB) is useful in diagnosing conditions such as kidney stones, performing a CT scan is necessary for evaluation of perinephric inflammation. It has also been reported that ultrasonography can be utilized to assess the presence of hydronephrosis or nephromegaly, and may also be useful as a diagnostic imaging method in cases of obstructive urinary tract infections.^39^

There are no randomized controlled trials (RCTs) evaluating the validity of whole body contrast‐enhanced CT examination in patients without apparent infectious foci. Yanagawa *et al*. have reported in a retrospective study that the detection rate for infectious foci was 38.8% when estimating by chief complaints and physical examination findings, whereas it increased to 88.8% when using whole‐body contrast‐enhanced CT examination in geriatric patients with suspicion of infection.^40^

In addition, in a retrospective study by Just *et al*. examining emergency room patients for whom the origin of infection was unknown,^41^ out of 144 CT photographs taken, infectious foci were identified in 76 (52.8%), of which 65 (85.5%) had undergone surgery in connection with the change in treatment plan. Based on the above, an expert consensus that performing whole‐body contrast CT examination is recommended when the infectious focus is unknown was reached.

It is known that the availability of CT apparatus per population is much higher in Japan in comparison with Europe and the United States. Therefore, it can be presumed that imaging by whole body contrast‐enhanced CT when the foci of infection are unknown is easy to perform. However, the risk of contrast‐induced nephropathy (CIN) may increase. No RCT has been conducted to evaluate the relationship between the administration of contrast media and CIN in patients with sepsis or septic shock. Therefore, the existence of a causal relationship is not clear. In a systematic review/meta‐analysis performed in 2013, McDonald *et al*.,^42^ found that the relative risk (RR) of acute kidney injury (AKI) development, requiring intermittent hemodialysis, and mortality were 0.79, 0.88, 0.95, respectively, and no significant differences were observed (15582 patients exposed to contrast agents, 10368 patients not exposed.). Ng *et al*.^43^ and Polena *et al*.^44^ have reported in retrospective studies that the incidence of AKI development after contrast media administration did not increase in ICU patients. Therefore, it is unlikely that the frequency of the onset of AKI increases after intravenous administration of a contrast agent in comparison to patients who were not injected with a contrast agent.

However, the guideline for the use of iodine contrast medium in patients with kidney injury^45^ states that in patients with impaired renal function, (i) reduction in the amount of contrast agent used, and (ii) performing fluid transfusion prior to conducting the contrast CT, may reduce the likelihood of CIN onset. Nevertheless, because there is a large amount of information concerning CT examination using a contrast agent and this technique is an important method of diagnosing infections and determining a therapeutic approach, there is no need to hesitate to perform contrast‐enhanced CT examinations due to concern over the onset of CIN.

### CQ 3‐1: Should imaging examinations be used to diagnose the foci of infection?

#### Answer (opinion)

The use of imaging examinations is recommended in the diagnosis of the foci of infection in sepsis/septic shock patients (expert consensus/no evidence; rate of agreement: 100%).

#### Rationale

There is currently no supporting RCT that conforms to the PICO process, and there is little evidence available in support of performing diagnostic imaging. The detection of infectious foci is important in sepsis and septic shock. If the diagnosis of an infectious focus can be performed accurately through imaging, the optimal treatment method can be selected, and unnecessary treatments can be avoided. However, various complications may also occur, such as allergic reaction to the iodine‐based contrast agents, impaired renal function, or gadolinium‐based contrast‐associated nephrogenic systemic fibrosis, and caution is required when treating patients who are at risk. In addition, there are some concerns that the condition of patients with unstable hemodynamics and respiration might worsen when they are transported to the examination room. In consideration of the above, in patients with sepsis and septic shock, “performing imaging examinations for diagnosis of infectious foci is recommended (expert consensus)” while paying attention to the complications and dangers associated with patient transportation.

### CQ 3‐2: Can early‐stage (whole body contrast) CT examination be useful when the foci of infection are unknown?

#### Answer (opinion)

Performing early (whole body contrast enhanced) CT examination is recommended to aid in diagnosing the foci of infection in patients with sepsis/septic shock (expert consensus/no evidence) (rate of agreement: 89.5%).

#### Rationale

There is no RCT that conforms to the Patient, Intervention, Comparison, Outcome (PICO) process, and the evidence for performing whole body contrast‐enhanced CT at an early stage is poor. The diagnosis of the foci of infection is important for the diagnosis of sepsis/septic shock, but sometimes, it is difficult to determine the origin of infection based on simple CT examination alone. Infectious foci become apparent on contrast‐enhanced CT images, which can lead to the selection of a more effective treatment for the infection. However, various complications may also occur, such as allergic reaction to the iodine‐based contrast agent, impaired renal function, or gadolinium‐based contrast‐associated nephrogenic systemic fibrosis, and caution is required when treating at‐risk patients. In sepsis and septic shock, “performing CT scans (whole body contrast enhanced) at an early stage is recommended (expert consensus).” while paying ample attention to possible complications.

## CQ4: CONTROLLING THE ORIGIN OF INFECTION

### Introduction

The two basic principles guiding the approach to controlling infectious foci are that measures should be taken “early” and should be “effective while minimally invasive.” This guideline offers a discussion about determining the source of infection, which is key to controlling it. In addition, the following five examples of infection sources are evaluated: (i) intra‐abdominal infection, (ii) infectious pancreatic necrosis, (iii) vascular catheter‐associated infection, (iv) acute pyelonephritis resulting from ureteral obstruction, and (v) necrotizing soft tissue infection. The clinical questions (CQs) accompanying this guideline were formulated based on these discussion components. It was concluded that each infection source exhibits clear and distinct characteristics after compiling research findings regarding their respective methods of control. As having a deep understanding of these characteristics is believed to be helpful when attempting to control infections, specific details of each example are provided in their corresponding CQ.

No randomized controlled trial (RCT) has been conducted to date to compare the prevalence of surgery to address intra‐abdominal sepsis between two groups. However, prospective multicenter observational studies examining factors related to outcomes of cases of generalized peritonitis have reported that the success or failure in controlling the foci of infection has the highest odds ratio pertaining to patient outcome.^46^ The Surviving Sepsis Campaign Guidelines (SSCG) 2012^23^ as well as guidelines published by the Infectious Diseases Society of America (IDSA) and the Surgical Infection Society (SIS) regarding intra‐abdominal infections^47^ each emphasize the importance of achieving adequate control of intra‐abdominal infections sources. As no RCT demonstrating the efficacy of achieving early control of infected lesions has been conducted to date, this guideline discusses the results of a systematic review and one observational study. This study targeted cases in which intra‐abdominal infection persisted following surgical intervention, and conducted a two‐group comparison of the elapsed time until reoperation. The results indicated that the mortality rate was lower in the group that underwent reoperation sooner.^48^ In addition, the 30‐day mortality rate rises by 2.4% for each hour treatment is delayed for an intra‐abdominal infection arising from peptic ulcer perforation,^49^ and extension of the preoperative period has been linked to poor outcomes in patients presenting with septic shock caused by gastrointestinal perforation.^50^ Accordingly, achieving control of the focus of infection as soon as possible is considered to be the favored approach when treating cases of sepsis arising from intra‐abdominal infection.

Regarding the classification of local pancreatic complications accompanying acute pancreatitis, in the 2012 revision of the Atlanta Classification,^51^ peripancreatic fluid collections can be categorized into “fluid collections” pertaining to the liquid component only (which causes interstitial edematous pancreatitis), or “necrotic collections” (occurring after the onset of necrotizing pancreatitis), referring to solid components mixed with necrotic materials and liquids. “Fluid collections” may be further categorized as acute peripancreatic fluid collections within the first 4 weeks after onset and pseudocysts after the first 4 weeks, and “necrotic collections” may be categorized as acute necrotic collections within the 4 weeks after onset and walled‐off (pancreatic) necrosis after the first 4 weeks. In addition, infectious pancreatic necrosis has been reported to be accompanied by acute necrotic collections or bacterial/fungal infections in conjunction with walled‐off necrosis as described previously.^51^ Based on this classification, any significance of performing early (within 72 h after onset) surgery in necrotizing pancreatitis cases can be ruled out with respect to achieving control of the source of infection, and reports state that as a general rule, conservative treatment should be offered, and interventional treatment is appropriate when necrotizing pancreatitis is complicated by infection (infectious pancreatic necrosis). Therefore, both the timing of treatment and method were evaluated in the context of controlling the source of infection in cases of infectious pancreatic necrosis.

An indwelling vascular catheter can be a source of infection. Accordingly, a CQ was prepared to examine the types of cases in which early removal of a vascular catheter is recommended after it is determined to be a source of infection. Early removal of a vascular catheter is limited to cases where bloodstream infection has been confirmed or when a patient's hemodynamics have become unstable with the aim of reducing instances of unnecessary removal of vascular catheters, which is believed to reduce both medical costs and risks to patients associated with reinsertion. According to the 2009 IDSA guideline,^52^ routine catheter removal should not be performed (B‐II) in ICU patients based solely on the observation of novel fever symptoms not accompanying severe sepsis or bloodstream infection findings. However, in the event of other unexplained sign of sepsis or redness/suppuration at the catheter insertion site, the central venous catheter (and the arterial catheter if placed) should be removed (B‐II). Based on these recommendations, early removal of vascular catheters is believed to be beneficial only for patients in whom a vascular catheter was placed as part of sepsis treatment where bloodstream infection has been confirmed or where hemodynamics have become unstable, and not when a bloodstream infection is merely suspected.

Pyelonephritis caused by obstruction of the ureter is one of the several conditions requiring control of the source of infection. No RCTs were found to examine whether infections in patients who developed sepsis due to pyelonephritis caused by ureteral obstruction should be controlled more quickly. However, removal of the ureteral obstruction can be an effective means of controlling the infection source, and therefore reopening the ureter as quickly as possible is believed to be beneficial. Guidelines published by the American Urological Association and the European Association of Urology^53^, ^54^, ^55^ both recommend swift cystectomy at grade A in cases of sepsis caused by urinary tract obstruction due to ureteral calculus and although there is no RCT‐based evidence, the importance of taking action quickly is widely accepted. Treatment methods for this condition also include percutaneous nephrostomy and transurethral ureteral stent placement. While target patients are those who have contracted infection as a result of ureteral calculus obstruction rather than sepsis patients, both methods were shown to be equally effective in a small‐scale RCT conducted by Pearle *et al*.^56^ (1998, enrolling a total of 42 subjects). Both of the guidelines mentioned previously^53^, ^54^, ^55^ also support this result. Based on these observations, it is believed that quickly achieving control of the origin of infection through approaches such as percutaneous nephrostomy or transurethral ureteral stent placement is beneficial in cases of sepsis caused by acute pyelonephritis arising from ureteral obstruction.

No RCT could be found that compared the usefulness of achieving early source control in sepsis caused by necrotizing soft tissue infection, although there exist guidelines^57^, ^58^ and a review^59^ on this subject. Although early diagnosis and administration of broad‐spectrum antimicrobials can be effective in improving the prognosis of patients with necrotizing soft tissue infection, when treating patients with organ dysfunction arising from necrotizing soft tissue infection, that is, patients with sepsis, surgical intervention including swift and aggressive drainage of infected lesions is recommended by two different guidelines.^57^, ^58^ A review study examining the timing of surgical procedures also suggests that initiating surgery within 24 h after diagnosis can improve the mortality rate by ~20% more than surgeries performed after this period.^59^ If clinical symptoms persist after surgery, practical guidelines^57^ recommend performing reoperation while continuing antimicrobial administration for an additional 24–36 h. Based on the above, it is believed that surgery should be initiated at the earliest opportunity in cases of sepsis arising from necrotizing soft tissue infection.

### CQ4‐1: What approach should be taken to control the source of intra‐abdominal infection?

#### Answer (opinion)

Controlling the source of infection as soon as possible is recommended in cases of sepsis arising from intraperitoneal infection (expert consensus/quality of evidence “D”; rate of agreement: 100%).

#### Rationale

No RCTs conforming to the Patient, Intervention, Comparison, Outcome (PICO) process could be found, and so a systematic review of observational studies was conducted. As a result, one observational study was extracted.^48^ If sepsis arises from an intraperitoneal infection, controlling the source of infection at an early stage may improve patient outcome. Performing surgery to control the infection is invasive to the patient, but it is believed that no side effects will result if the surgery is performed early. Based on this study, it was concluded that performing early surgery may improve patient outcomes and that the benefits to patients outweigh the potential harms.

### CQ4‐2: What approach should be taken to control the source of infectious pancreatic necrosis?

#### Answer (recommendations and opinion)

1. We suggest waiting to perform interventional treatment until week 4 after onset that acute necrotic collections become walled‐off, in cases of sepsis arising from infectious pancreatic necrosis with stable general condition (2C; rate of agreement: 100%).
2. We suggest performing interventional treatment without waiting until week 4 after onset, in cases of sepsis arising from infectious pancreatic necrosis with unstable general condition (expert consensus/no evidence; rate of agreement: 100%).
3. We suggest performing drainage first (percutaneously or endoscopically) and then resection of necrotic tissue (via retroperitoneal or endoscopic approaches) if improvement is not seen (2C; rate of agreement: 100%).

#### Rationale

Infectious pancreatic necrosis is a disease in which the early initiation of intervention, the usual principle in controlling the source of infection, does not apply. In an RCT comparing mortality rates with regard to differences in the timing of treatment approaches to control the source of infection,^60^ 36 patients presenting with severe necrotizing pancreatitis were included in the early intervention group and underwent necrotic tissue resection 48–72 h after onset, while the late intervention group underwent surgery 12 days after onset. As a result of the comparison, the mortality rate was lower in the late intervention group compared to the early intervention group.^60^ Two RCTs have been reported on the treatment of infected pancreatic necrosis.^61^, ^62^ In the first RCT, the minimally invasive step‐up approach to treating infectious pancreatic necrosis was compared with open necrosectomy, and no significant difference in mortality rates was observed (19% versus 16%). However, the ICU stay times and hospitalization times associated with the minimally invasive step‐up approach tended to be shorter. Regarding the frequency of complications, few incident cases of multiple organ failure or general complications, intraperitoneal bleeding requiring treatment, enterocutaneous fistula requiring treatment, or perforation into intraperitoneal organs were observed in the minimally invasive step‐up approach group (a significant difference was observed with respect to the incidence of multiple organ failure and systemic complications). The second RCT was a comparison of endoscopic transgastric necrosectomy and surgical necrosectomy. As a result, it was found that the mortality rate was lower in the endoscopic transgastric necrosectomy group, and the incidence of complications such as multiple organ failure, intraperitoneal bleeding requiring treatment, enterocutaneous fistula requiring treatment, perforation into intraperitoneal organs, and pancreatic fistula was low in the endoscopic transgastric necrosectomy group. A significant difference was observed with respect to the incidence of multiple organ failure and pancreatic fistulas. Although there was no difference in survival outcomes between these two RCTs, the effectiveness of a minimally invasive approach was demonstrated by the reduction in the incidence of complications.

Based on the above observations, controlling infected lesions in patients with sepsis due to infectious pancreatic necrosis by first performing drainage (percutaneously or endoscopically) and then resecting necrotized tissue (via retroperitoneal or endoscopic approaches) is considered to be beneficial.

### CQ4‐3: What circumstances call for the early removal of vascular catheters in patients with sepsis?

#### Answer (recommendation)

We suggest removing vascular catheters only when bloodstream infection is suspected (2D; rate of agreement: 94.7%).

#### Rationale

One RCT^63^ was found as the result of a comprehensive literature search. In this study of 144 patients in whom vascular catheter‐related bloodstream infection was suspected, 64 patients (excluding 80 cases predicted to have been caused by vascular catheter infection) were divided into two groups (with 32 patients each). As a result, no significant difference in ICU mortality rate was observed. Accordingly, unnecessary vascular catheter removals can be reduced by restricting early withdrawals to cases when bloodstream infection is confirmed or when the patient becomes hemodynamically unstable. Such measures can be expected to lead to reductions in medical costs and risks arising from catheter reinsertion. However, it has been reported that after a catheter‐related bloodstream infection is diagnosed, removal of the catheter within 24 h is associated with improved patient outcomes.^64^ Based on these observations, the early removal of a vascular catheter from a patient with sepsis is considered to be beneficial only in cases where a bloodstream infection has been confirmed, or the patient has become hemodynamically unstable.

### CQ4‐4: What approach should be taken to control the source of infection in cases of sepsis arising from acute pyelonephritis resulting from ureteral obstruction?

#### Answer (opinion)

Controlling the source of infection as quickly as possible via percutaneous nephrostomy or transurethral ureteral stent placement is recommended in cases of sepsis arising from acute pyelonephritis caused by ureteral obstruction (expert consensus/no evidence; rate of agreement: 94.7%).

#### Rationale

As no RCTs conforming to the PICO process could be found, this CQ referred to the American Urological Association (AUA) guidelines.^53^ When considering the treatment of acute pyelonephritis caused by ureteral obstruction as well as the costs of transporting patients to specialist facilities, it is believed that the potential benefits obtained by performing a percutaneous nephrostomy or transurethral ureteral stent placement likely outweigh potential complications such as bleeding or the spreading of infection to the retroperitoneum.

### CQ4‐5: What approach should be taken to control the source of necrotizing soft tissue infection?

#### Answer (Opinion)

Proceeding with early surgical intervention is recommended in cases of sepsis arising from necrotizing soft tissue infection (expert consensus/no evidence; rate of agreement: 100%).

#### Rationale

No RCTs conforming to the PICO process could be found. When complicated by organ failure due to necrotizing soft tissue infection (i.e., cases of sepsis) it is likely that initiating surgical intervention including aggressive and early drainage of the infected lesion will be more beneficial to the patient. Although there is a risk of harm caused by the surgery, the benefits outweigh the risk, compared to when surgery is not performed despite the development of sepsis. Therefore, although no RCT conforming to the PICO process could be found, it was concluded that there is a strong possibility that the benefits outweigh the potential harms.

## CQ5. ANTIMICROBIAL THERAPY

### Introduction

Antimicrobial therapy is an essential fundamental component in the management of sepsis. One concern related to antimicrobial use is the threat of drug‐resistant bacteria. The excessive use of antimicrobials is linked to a greater risk of loss of effective drugs in the future due to the emergence of drug‐resistant bacteria. These guidelines were formulated with specific regard to the management of sepsis cases, and do not offer guidance related to antimicrobial drug selection. However, the selection of antimicrobials in sepsis cases is similar in principle to the treatment of general infections. Antimicrobials should be selected based on factors such as the patient's background, organs suspected to be affected, epidemiological information pertaining to the region and the medical facility, and recent history of antimicrobial use, after anticipating to the extent possible the specific microbial strain to be targeted, as well as any drug resistances. However, prompt administration of an effective antimicrobial targeting the causative microorganism is more critical in comparison to non‐severe cases. The issue of microbial drug resistance also warrants consideration, and consultation with an infectious disease specialist is also important at facilities where such specialists are available.

The evidence currently available for the clinical question (CQ) “Should antimicrobial therapy be initiated within 1 h?” was reexamined and a recommendation offered by the Guideline Creation Committee. According to the results of a retrospective cohort study, the mortality rate among septic shock patients increases by 7.6% for each hour antimicrobial administration is delayed.^65^ In addition, in emergency outpatient sepsis cases, time to initiation of antimicrobial therapy and patient mortality were factors related to the severe patient group.^66^ Contrastively, in a meta‐analysis of observational studies, no benefit was found with respect to mortality risk in patients who received antimicrobial drugs within 1 h of shock onset.^67^ However, we believe that abandoning the widely‐accepted clinical target of initiating antimicrobial therapy within 1 h based on the results of a meta‐analysis of observational studies is inappropriate.

Combination therapy in the context of antimicrobial therapy refers to antibiotic combination therapy targeting Gram‐negative bacilli. In addition to the therapeutic effects of combination therapy, the recommendation was evaluated with emphasis on the potential risks of treatment, such as kidney injury. As a result, these guidelines recommend against the routine use of combination therapies. However, physicians should decide whether to use such therapies on a case‐by‐case basis when handling refractory infection cases involving multidrug‐resistant Gram‐negative bacilli, origination from artificial materials, or immunocompromised patients.

Regarding the various types of antifungal therapy, a CQ specifically addressing anticandidal therapy was judged to be beyond the scope of these guidelines on the reasoning that such infections and other fungal infection cases requiring intensive care were infrequent and that expert knowledge and experience may be required depending on the decisions made regarding the initiation of treatment. Known risk factors for deep Candida infection include deposition of live Candida into the body, artificial ventilation, high Acute Physiology and Chronic Health Evaluation (APACHE) II score, use of broad‐spectrum antimicrobials, use of immunosuppressants, central venous catheter use, total parenteral nutrition, neutropenia (<500/mm^3^), recent surgery (especially gastrointestinal surgery), renal failure, hemodialysis, malnutrition, severe acute pancreatitis, diabetes, recent organ transplantation, indwelling urinary catheter use, advanced age, chemotherapy, malignant tumor presence, and the use of antacids.^68^, ^69^, ^70^, ^71^ The combined use of anticandidal drugs as well as conventional antimicrobials should be considered when handling sepsis cases involving patients exhibiting more than one of these risk factors. Whether physicians should consider serum (1‐3)‐β‐d‐glucan values when determining whether to add anticandidal drugs when treating sepsis patients exhibiting the aforementioned risk factors remains unclear, and is a question to be addressed in the future.

The bactericidal action and therapeutic effects of β‐lactam drugs correspond to periods when the drug serum concentration exceeds the minimum inhibitory concentration (MIC) of the target bacteria. In view of this characteristic, extended infusion time or continuous infusion lengthens the drug's time above MIC (the proportion of time within a 24‐h period during which drug serum concentration exceeds the applicable MIC), and these techniques are expected to result in superior clinical efficacy.^72^ In environments such as the intensive care unit (ICU) in particular, pathogenic bacteria tend to exhibit a higher MIC, raising concern that intermittent infusion, a standard practice in many care settings, will be unable to achieve sufficient time above MIC.^73^ The respective efficacy profiles of the continuous infusion, extended infusion, and intermittent infusion methods of drug administration were evaluated during meta‐analysis, and as a result, no significant differences were observed between ICU mortality rate, in‐hospital mortality rate, and rate of achievement of the target drug serum concentration. Accordingly, we believe that consideration of utilizing continuous infusion of β‐lactam antibiotics has low significance.

As there are some concerns regarding the safety of the de‐escalation approach in Japan, we decided to offer recommendations after reorganizing our findings. De‐escalation is supported by the results of numerous observational studies. The first randomized controlled trial (RCT) enrolling sepsis patients, albeit in a small number, was completed only recently, and as a result, de‐escalation had no observable impact on either total ICU stay time or 90‐day mortality rate.^74^ Based on the above, de‐escalation can be assumed to be safe, and these guidelines suggest that physicians implement de‐escalation in the usual manner.

Decreased procalcitonin (PCT) levels have been reported to be linked to a lower risk of mortality,^75^, ^76^, ^77^ and active research efforts have focused on instances where the decision to discontinue antimicrobial therapy regimens is made based on a protocol using PCT values to determine whether the period of antimicrobial drug use can be shortened without negatively influencing turning points in a patient's course. We referred to 9 RCT reports during our meta‐analysis on this topic.^78^, ^79^, ^80^, ^81^, ^82^, ^83^, ^84^, ^85^, ^86^ No significant differences were observed between the intervention and control groups with respect to ICU stay time, hospitalization period, 60‐day mortality rate, and 90‐day mortality rate. However, a significant improvement in the 28‐day mortality rate was observed. The duration of antimicrobial use in days was also significantly shortened. Based on the above, the use of PCT values in determining whether to discontinue antimicrobial therapy in sepsis cases is suggested, as the potential benefits outweigh the potential risks.

Typical antimicrobial treatment periods and the rationales for decisions to discontinue such treatment in sepsis cases may differ by country. Meanwhile, no Japanese RCTs investigating the discontinuation of antimicrobial therapy based on PCT values have been completed to date. Whether basing discontinuation decisions on PCT values can reduce the period of antimicrobial use or improve survival prognosis also remains unclear in sepsis treatment in Japan. We expect that research in these areas will progress in the years to come.

Lastly, it is known that the pharmacokinetic properties of antimicrobial drugs can change drastically in sepsis patients as a result of vital reactions and therapeutic interventions.^87^ As such, it may become necessary to reduce or increase dosage or to extend or shorten the administration interval more than has conventionally been believed when treating sepsis patients. Although this is a critical area of concern, current research activity is inadequate. Because of this, we determined that a recommendation and a CQ addressing this topic could not be offered at this time.

### CQ5‐1 Should antimicrobial therapy be initiated within 1 h after recognition of sepsis?

#### Answer (Opinion)

Sepsis and septic shock patients should begin receiving an effective antimicrobial within 1 h (expert consensus/no evidence; rate of agreement: 100%).

#### Rationale

Initiating antimicrobial therapy within 1 h when handling sepsis cases is now recommended in the Surviving Sepsis Campaign Guidelines based on the results of observational studies, and has gained global acceptance. However, it is also true that there is no particularly strong basis for this recommendation, as no relevant RCTs have been completed to date. As such, although we have strong reservations regarding the possibility of negatively impacting patient prognosis by refraining from promptly administering antimicrobials in sepsis cases, we decided that it was necessary to offer our opinion as a target although it comes in the form of an expert consensus.

No RCTs investigating the impact of antimicrobial administration within 1 h could be found, and only results of observational studies were considered as evidence. Although the results of multiple observational studies indicate that initiation of antimicrobial therapy within 1 h or earlier reduces the risk of mortality, no significant improvement in mortality risk was observed in a systematic review of such observational studies.^67^

Initiating antimicrobial therapy within 1 h after diagnosis may contribute to a lower risk of mortality, and no associated adverse effects have been reported. The increased burden placed on medical staff when antimicrobial therapy is ordered to be initiated within 1 h after diagnosis arising from the need to prioritize the corresponding preparatory tasks over others (e.g., confirmation of drugs dispensed and transportation from the hospital pharmacy) may be considered as an assumed burden. Another obstacle is the issue of space limitations for drug storage accompanying the need to routinely prepare a variety of antimicrobials for emergency outpatients. Even with the above considered, we believe that the potential benefits of this practice likely exceed any potential harms.

### CQ5‐2 Should combination therapy be used when administering empirical antimicrobial therapy in sepsis cases?

#### Answer (recommendation)

We recommend against routinely administering antimicrobial combination therapy when treating infections caused by Gram‐negative bacilli (1B; rate of Agreement: 89.5%).

#### Rationale

In the past, there has been a view that combination therapies using antimicrobial drugs for sepsis and septic shock cases, especially in the treatment of Gram‐negative bacilli, will expand the antimicrobial spectrum and that a synergistic effect should be expected. However, due to the considerable risks associated with antimicrobial combination therapies, it was important to present an opinion based on clear reasoning that was also reflective of the realities of clinical practice.

We referred to a single meta‐analysis that verified the effects of using aminoglycosides in combination with β‐lactam drugs.^88^ No difference in mortality rate was observed for monotherapy in comparison to combination therapy, but a significant increase in the frequency of kidney injury, believed to be a side effect of aminoglycoside antimicrobials, was observed with respect to the use of combination therapy. In addition to this meta‐analysis, another RCT verified the effect of using a quinolone antimicrobial (moxifloxacin) in combination with a carbapenem (meropenem), a β‐lactam antibiotic.^89^ This study found that while mortality rate remained unchanged as a result of using this combination therapy, the frequency of side effects associated with these drugs increased.

No significant difference in mortality rate was observed between the intervention and control groups in this study, and apart from there being no observable benefit, the frequency of kidney injury was significantly higher in patients receiving combination therapy compared with those who received monotherapy only. The development of new onset kidney injury may increase patient burden as well as medical costs as a result of the greater need for related treatment interventions. In addition, in consideration of the time and cost of prescribing, dispensing, and administering multiple antimicrobials, the potential harms associated with this practice clearly outweigh the benefits.

### CQ5‐3 In what situations should anticandidal drug therapy be initiated?

#### Answer (Opinion)

The administration of anticandidal drugs in addition to general antimicrobials should be considered when treating sepsis and septic shock patients exhibiting multiple risk factors for invasive candidiasis (expert consensus/no evidence; rate of agreement: 78.9%).

#### Rationale

It is known that the *Candida* genus of fungi is a primary cause of fungal sepsis, and also that the mortality rate associated with candidemia is higher than the rates attributed to other forms of bacteremia. Despite this, candidiasis is also frequently overlooked. As such, it is necessary to establish criteria for administering anticandidal drugs when handling cases refractory to conventional antimicrobial therapies.

No RCTs evaluating the use of anticandidal drugs in sepsis cases could be found, and the evidence considered for this CQ considered candidemia or invasive candidiasis. Multiple observational studies have been conducted with respect to the known risk factors for these conditions, and risk factors specific to ICU patients have also been reported. In addition, the sensitivity and specificity of (1‐3)‐β‐d‐glucan, a serum biomarker, have also been evaluated in the context of invasive candidiasis.

The administration of antifungal drugs following risk assessment may improve patient prognosis in invasive candidiasis or candidemia cases, but at the same time, poses a risk of adverse reactions. However, no assessment of this risk as it pertains to sepsis patients has been conducted to date. In consideration of the above, we believe that the potential benefits of anticandidal drug use likely outweigh the potential risks.

### CQ5‐4 Should β‐lactam drugs be continuously infused or should their infusion period be extended when treating sepsis or septic shock patients?

#### Answer (Recommendation)

We suggest against administering β‐lactam drugs using continuous infusion or extended infusion periods when treating sepsis and septic shock patients (2B; rate of agreement: 100%).

#### Rationale

To date, intermittent administration of antimicrobial drugs has been a common practice. However, it has been found that time‐dependent β‐lactam drugs may be more effective in terms of pharmacokinetic characteristics when administered continuously or over an extended period. Verification of the efficacy of continuous infusion of β‐lactam drugs may lead to improved patient outcomes in sepsis cases and is considered to be an important clinical issue.

We referred to 4 RCT reports.^72^, ^90^, ^91^, ^92^ Among these studies, no significant differences were observed between the respective study groups with respect to 90‐day mortality rate (odds ratio: 0.94; 95% confidence interval [CI]: 0.69–1.28, *P *=* *0.68), ICU mortality rate (odds ratio: 0.79; 95% CI: 0.59–1.06, *P *=* *0.11), in‐hospital mortality rate (odds ratio: 0.78; 95% CI: 0.59–1.03, *P *=* *0.08), or target serum drug concentration achievement rate (odds ratio: 1.88; 95% CI: 0.89–3.98, *P *=* *0.10).

Although decreased mortality frequency is an anticipated benefit of this intervention, no significant differences were observed between the intervention and control groups in any study regarding 90‐day mortality rate, in‐hospital mortality rate, and ICU mortality rate. In addition, no significant differences were observed with regard to target serum drug concentration achievement rate. However, although no evaluation of side effects was conducted, because β‐lactam drugs are normally administered to ICU patients via intravenous infusion, we believe that few burdens that arise from this intervention warrant consideration. We have determined accordingly that the risks and benefits associated with this intervention are comparable.

### CQ5‐5 Is de‐escalation a recommended approach with respect to antimicrobial therapy for sepsis and septic shock patients?

#### Answer (Recommendation)

We suggest the use of de‐escalation in conjunction with antimicrobial therapy administered to sepsis and septic shock patients (2D; rate of agreement: 84.2%).

#### Rationale

Although broad‐spectrum antimicrobials are frequently given at an early stage to address sepsis cases in the ICU, this practice is linked to the appearance of drug‐resistant bacteria and accompanying increases in medical costs. As such, the capacity to de‐escalate, or switch treatment regimens from broad‐spectrum antimicrobials to drugs with narrower therapeutic indices, without risking patient safety, can be regarded as a favorable practice from the perspectives of both infection control and medical economics.

We referred to one RCT report.^74^ No significant differences were observed with respect to 90‐day mortality rate between the 2 groups, and a significant increase in the frequency of coinfection was observed in the de‐escalation group.

The primary benefit expected as a result of de‐escalation is the prevention of the development of drug‐resistant bacteria, but this outcome could not be evaluated based on this body of evidence. Meanwhile, although de‐escalation did not increase mortality rate, the results suggested that it may increase patients’ risk of contracting coinfections. However, all of the evidence considered originated from a single RCT; thus we believe this body of evidence lacks the strength necessary to constitute a basis for overriding the notion that de‐escalation can be implemented safely, which is based on the results of observational studies conducted to date. In consideration of the above, we have determined that the potential benefits of de‐escalation likely exceed any potential harms.

### CQ5‐6 Should PCT values be used as an index to determine whether to discontinue antimicrobial therapy?

#### Answer (Recommendation)

We suggest using PCT values as an index when determining whether to discontinue antimicrobial therapy administered to address sepsis or septic shock (2B; rate of agreement: 78.9%).

#### Rationale

The measurement of PCT levels has become feasible in routine treatment, and studies investigating the use of PCT values in cases of infection have also increased; RCTs examining the discontinuation of antimicrobial therapy in accordance with the PCT guide have been conducted. However, there is still a paucity of high‐quality systematic reviews of the subgroup of these RCTs that focused on sepsis cases. To establish criteria for discontinuing antimicrobial therapy in sepsis cases, the validity of interventions calling for antimicrobial therapy discontinuation based on PCT values, particularly those accumulated by RCTs, must be evaluated.

We referred to 9 RCT reports.^78^, ^79^, ^80^, ^81^, ^82^, ^83^, ^84^, ^85^, ^86^ No significant differences were observed between the intervention and control groups with respect to ICU stay time, hospitalization period, 60‐day mortality rate, and 90‐day mortality rate, but significant improvement in 28‐day mortality rate was observed. The number of days of antimicrobial drug use also decreased significantly (a meta‐analysis was conducted only with respect to studies that clearly described the mean administration period. Significant reduction in the number of days of therapy was also observed in the studies providing median value data). In the intervention group, no significant increase in mortality rate or the period of antimicrobial drug use was observed in comparison to the control group. Potential harms could not be considered as no other side effects were assessed. PCT is measured in conjunction with other blood parameters and can be grouped among routine blood tests. As such, we believe that this intervention adds minimal additional burden, and its potential benefits likely exceed any potential harms.

## CQ6: INTRAVENOUS IMMUNOGLOBULIN (IVIG) THERAPY

### Introduction

IVIGs comprise antibodies specific to various bacteria, toxins, and viruses. In addition to exerting an opsonic effect and complementary component activation when bound to antigen particles, IVIGs also have a neutralizing effect on toxins and viruses and inhibit inflammatory cytokines.^93^, ^94^ In 60% of septic shock patients, apparent hypogammaglobulinemia (serum IgG level <650 mg/dL) is present from the beginning, due to suppressed immunoglobulin production, protein leakage, and exhaustion.^95^ Although serum IgG level has been linked to the incidence of shock and mortality rate of patients entering the intensive care unit (ICU),^96^ IVIG administration, when used in combination with adequate antibiotics and appropriate fluid resuscitation, may improve the survival of patients with sepsis.^97^

According to the results of a study conducted by Masaoka *et al*.^98^ in Japan, IVIG is listed in the National Health Insurance Registry as a supplementary treatment for severe infections, and as such, IVIGs are often administered in patients with severe sepsis/septic shock. A logistic regression analysis to investigate whether early IVIG administration within 48 h of onset affects the 28‐day survival rate of patients with septic shock was conducted by the Special Sepsis Registry Committee of the Japanese Association for Acute Medicine using the data of 624 patients with severe sepsis between May 2009 and May 2011. Early IVIG administration was found to be an independent factor contributing to improved prognosis (odds ratio: 1.904, 95% confidence interval (CI): 1.044–3.471, *P *=* *0.036),^99^ supporting the assertion that IVIG administration results in improved prognosis in cases of severe sepsis. In contrast, Tagami *et al*. used Diagnosis Procedure Combination (DPC)‐based data of patients with septic shock requiring mechanical ventilation to extract 1081 cases involving emergency laparotomy to address lower gastrointestinal perforation^100^ and 1045 cases of severe pneumonia^101^ and examined the 28‐day mortality rate through propensity analysis. As a result, Tagami, *et al*. reported no significant improvement in the IVIG administration group (emergency laparotomy: IVIG group: 20.6% versus control group: 19.3%; 95% CI: −2.0 to 4.5; severe pneumonia: IVIG group: 36.7% versus control group: 36.0%; 95% CI: −3.5 to 4.8). However, DPC data alone does not give detailed information such as the definition of sepsis, the severity (Acute Physiology and Chronic Health Evaluation II score), and the time to administration of IVIGs after the onset of sepsis. Although there are reports suggestive of a prognostic improvement effect based on large‐scale retrospective studies, this effect has not yet been established.

### CQ6‐1: Should IVIG be administered to adult patients with sepsis?

#### Answer (Opinion)

The prognostic improvement effect of IVIG administration in adult patients with sepsis is unknown based on the current randomized controlled trial (RCT) results available, and accordingly clear recommendations pertaining to IVIG administration cannot be offered (expert consensus/quality of evidence “C” ; a rate of agreement of 67% or higher in support of its use could not be obtained).

#### Rationale

While formulating this clinical question, it was considered to be critical to examine the effectiveness of immunoglobulin administration to patients with sepsis while considering benefits based on reductions in all‐cause mortality rate, ICU mortality rate, and ICU treatment period, and side effects caused by IVIG administration as potential harms.

In the literature search, 978 references are screened without limits on investigation period, the severity of sepsis, or IVIG dosage. Six papers were secondarily extracted via peer review of abstracts.^98^, ^102^, ^103^, ^104^, ^105^, ^106^ The all‐cause mortality rate in the IVIG group was significantly lower than that of the control group (*n* = 6, risk ratio: 0.7 [95% CI: 0.56–0.95]) and the ICU mortality rate was also significantly lower (*n* = 1, risk ratio: 0.71 [95% CI: 0.60–0.84]). Reduced ICU stay time, which was the second most important and significant benefit, was also shortened significantly (*n* = 3, mean difference: −3.71 [95% CI: −7.32 to −0.09]). There was no significant increase in the risk ratio for the onset of side effects due to IVIG administration (1.63, 95% CI: 0.65–4.11). The side effects were minor symptoms such as skin rash, and no serious cases or deaths were reported. IVIG administration to adult patients with sepsis resulted in improved all‐cause mortality and ICU mortality rates and also significantly shortened ICU stay time without increasing the frequency of side effects in comparison to the control group.

When considering the benefit‐risk balance in terms of outcomes, although there was an increase in complications, emphasis was placed on reduction in the all‐cause mortality rate and ICU mortality rate, and it was determined that the potential benefits likely outweigh the potential harms. However, members of the guideline committee expressed concerns about the quality of the systematic review and the body of evidence. Another body of evidence was proposed by the Academic Guidelines Promotion Team that narrowed the subjects to cases of severe sepsis and the internal peer review team proposed the 2013 Cochran Review.^107^ The team responsible for immunoglobulin treatments suggested that “IVIGs may be administered to adult patients with sepsis (2C [weak])”, but only a 63.2% agreement was obtained in the initial vote of the committee. The reasons for this were as follows: (i) we could not evaluate the effect as there were no new studies with current sepsis definitions and standard treatment; and (ii) although ICU mortality rates were improved based on the three bodies of evidence presented, there was no consistency with regard to evidence for the benefit in the 28‐day mortality among the three bodies of evidence. The rate of agreement was still 63.2% after the second committee vote, and the required proportion for agreement of an over two‐thirds majority was not obtained. The guideline committee reached an expert consensus that “the prognostic improvement effect of IVIGs in adult patients with sepsis is unknown based on the RCT results currently available, and a clear recommendation concerning IVIG administration cannot be presented at this time.”

## CQ7: INITIAL RESUSCITATION/INOTROPES

### Introduction

In response to infection, various self‐defense mediators are released. These mediators dilate peripheral vessels, resulting in a relative decrease in intravascular volume. As such, the treatment strategy for septic shock is focused on early‐stage control of infection (administering antimicrobials, gaining control of infected lesions) and appropriate control of circulation (improving cardiac output and oxygen supply, managing tissue hypoperfusion).

According to a meta‐analysis assessing goal‐directed therapy (GDT) that set target values and circulatory management for septic shock, the mortality rate was not reduced by achieving the goals alone, but was reduced if the goal was achieved within 6 h.^108^ Stated differently, time is a critical factor with respect to the effectiveness of initial resuscitation in septic shock. The early goal‐directed therapy (EGDT) capable of improving tissue hypoperfusion within 6 h introduced by Rivers *et al*.^109^ was strongly recommended in both the Surviving Sepsis Campaign Guidelines (SSCG) 2012^29^ and the Japanese Clinical Practice Guidelines for the Management of Sepsis and Septic Shock (1st edition).^2^ However, the three large‐scale randomized controlled trials (RCTs; Protocolized Care for Early Septic Shock [ProCESS],^110^ Australasian Resuscitation in Sepsis Evaluation [ARISE],^111^ and Protocolised Management in Sepsis [ProMISe]^112^) subsequently reported in 2014 and 2015 failed to demonstrate the usefulness of EGDT. As such, the guideline committee for this clinical question (CQ) conducted a systematic review based on the question, “CQ7‐1: Is EGDT recommended for initial resuscitation in patients with sepsis or septic shock?” The EGDT discussed herein refers to the resuscitation method proposed by Rivers *et al*.^109^ (calling for initial fluid resuscitation and administration of vasoconstrictors with the goal of achieving a central venous pressure (CVP) of 8–12 mmHg, mean arterial pressure ≥65 mmHg, urine volume ≥0.5 mL/kg/h, and ScvO_2_ ≥70% within 6 h).

A detailed assessment of these RCTs^110^, ^111^, ^112^ revealed that large‐volumes of fluid (crystalloid solution 30 mL/kg or more) had already been given before protocol initiation. Therefore, the guideline committee for this CQ concluded that the methods of initial fluid resuscitation should be assessed separately from the EGDT intervention, and the next CQ was presented, “CQ7‐2: What volume of fluid should be given in the initial resuscitation of patients with septic shock?”

Septic shock may be attributed not only to a relative decrease in intravascular volume associated with vasodilatation but also to a type of cardiomyopathy known as sepsis‐induced myocardial dysfunction (SIMD).^113^, ^114^ Therefore, “CQ7‐3: Should cardiac function be assessed using echocardiography when initiating fluid resuscitation in sepsis?” was presented, but no RCT conforming to the Patients, Intervention, Comparison, Outcome (PICO) process was found for this CQ.

The following two CQs were presented with regard to the fluid of choice for initial resuscitation and subsequent intravascular volume replacement in patients with septic shock, “CQ7‐4: Should a crystalloid solution or an artificial colloidal solution be used in the initial fluid resuscitation?” and “CQ7‐5: Should albumin solution be used during the initial resuscitation fluid in septic shock?” During the first public comment for CQ7‐5, it was pointed out that the directions of the recommendations offered and the results of the accompanying systematic review regarding mortality rate appeared to differ. The guideline committee reevaluated the evidence originating from the RCTs conforming to the PICO process only and found a slight improvement in survival associated with albumin administration. However, the strength of this evidence was considered to be weak, and albumin use in this context was found to have only a limited effect. The strength of the recommendation offered was determined by considering the potential for complications such as unknown infections and allergies caused by blood products. However, because the situations differ for patients requiring substantial amounts of crystalloids until shock recovery and those who develop hypoalbuminemia, we considered it necessary to deal with them separately and added an expert consensus.

With respect to monitoring during initial resuscitation, the following question, “CQ7‐6: What method should be used to predict fluid responsiveness during initial resuscitation?” was presented, and five RCTs conforming to the PICO process were analyzed. There were four interventions involving assessment through Passive Leg Raising (PLR), one intervention involving assessment through transpulmonary thermodilution, and two interventions (including redundancies) involving assessment through stroke volume variation (SVV). While a meta‐analysis was performed for each method of assessment, the meta‐analysis conducted for this CQ was unable to show any improvement in prognosis. The intrathoracic blood volume index obtained through the pulmonary thermodilution method^115^ as well as dynamic parameters such as SVV and pulse pressure variation have been reported to be more useful for the prediction of fluid response than CVP.^116^ However, caution is warranted when interpreting these findings, as test reliability is poor in patients with arrhythmias such as atrial fibrillation, patients with spontaneous respiration, and patients with restrictions in tidal volume during mechanical ventilation due to acute respiratory distress syndrome. PLR also has poor reliability in patients with elevated intra‐abdominal pressure.^117^

The practice guidelines reported so far^2^, ^29^ have highlighted the importance of measuring lactate levels as a marker of tissue hypoperfusion. This guideline also presents the following CQs, “CQ7‐7: Should lactate levels be used as an indicator during initial resuscitation in sepsis?” and “CQ7‐8: ScvO_2_ or lactate clearance: which is more useful as an indicator of initial resuscitation?” A systematic review was performed on the above CQs, but since only one RCT conforming to the PICO process could be found (Jones *et al*.^118^), it was judged that offering guidance at the recommendation level would be difficult for these CQs.

Regarding cardiovascular agents used in the management of septic shock, we considered two kinds of cardiovascular agents, vasopressors (dopamine, noradrenaline, adrenaline, vasopressin) and an inotropic drug (dobutamine). A systematic review and meta‐analysis were performed for the CQ “CQ7‐9: Noradrenaline or dopamine: which should be used as a first‐line vasopressor to treat patients with septic shock that are unresponsive to initial fluid resuscitation?” In addition, the subsequent two CQs are presented to address situations where noradrenaline use does not achieve a sufficient increase in blood pressure, “CQ7‐10: Should adrenaline be used in septic shock when noradrenaline fails to improve the blood pressure?” and “CQ7‐11: Should vasopressin be used in patients with septic shock who fail to achieve the target blood pressure despite the use of noradrenaline?”

Because the difference between the usage of adrenaline and vasopressin has not been established in the contents of the above CQ and an expert consensus, a brief supplement is provided on this topic. In septic shock, despite appropriate fluid resuscitation and noradrenaline administration, the following factors can create difficulty in maintaining hemodynamics: (i) difficulty in controlling peripheral vascular resistance accompanying vasodilatation (relative hypovolemic shock)^119^ and (ii) cardiac dysfunction associated with SIMD (cardiogenic shock).^113^, ^114^ These pathologies can be distinguished relatively easily through echocardiography. Administering adrenaline as well as a small amount of vasopressin (0.03 units/min) and noradrenaline is effective for patients exhibiting relative circulating hypovolemic shock (vasodilatory shock). On the other hand, in cases of cardiogenic shock, administering adrenaline to obtain a cardiac contractile potentiating effect (β1‐receptor stimulating action) can be effective, but administering vasopressin, which does not yield this effect, may cause further exacerbation of this pathological state leading to cardiogenic shock. For these reasons, appropriate vasopressors should be selected after assessing cardiac preload and contractility via techniques such as echocardiography in septic shock.

Meanwhile, it has been reported that in septic shock, intracellular signaling mediated by β1 adrenergic receptors is impaired due to early‐phase pro‐inflammatory cytokines, impeding the ability of dobutamine to improve cardiac function.^120^, ^121^ As such, with respect to dobutamine, an inotropic drug, the following CQ, “CQ7‐12: Should dobutamine be used in patients with septic shock who show evidence of cardiac dysfunction?” was presented and a systematic review was conducted. The 28‐day mortality rate in the RCTs^122^, ^123^ was 41.9% in the control group (adrenaline group) and 36.7% in the intervention group (dobutamine group; *P* = 0.31), and dobutamine was demonstrated to be comparable or non‐inferior to adrenaline. According to the SSCG 2012,^29^ dobutamine use is recommended (Grade 1C) (i) when cardiac function is declining, and (ii) in amounts of up to 20 μg/kg/min when low perfusion persists despite adequate fluid resuscitation. However, the Japanese Clinical Practice Guidelines for the Management of Sepsis and Septic Shock (1st edition)^2^ states that, “As improvement of reduced cardiac function is difficult to achieve with dobutamine in septic shock, combined administration with a phosphodiesterase III inhibitor or a calcium sensitivity enhancer should be considered as an alternative.” One RCT (the LeoPARDS [Levosimendan for the Prevention of Acute oRgan Dysfunction in Sepsis] trial) to evaluate calcium sensitivity enhancers in patients with sepsis was performed recently, but no prognostic improvement effect was observed.^124^ It was determined that the quality of the evidence supporting the recommendation of these drugs is currently poor, and accordingly, this guideline does not include a CQ on their use. Meanwhile, regarding the usefulness of β‐blockers in septic shock, Morelli *et al*.^125^ conducted an RCT evaluating ultra‐short‐acting β‐blockers, and Wang *et al*.^126^ conducted an RCT investigating the efficacy of combination therapy of an ultra‐short‐acting β‐blocker and a phosphodiesterase III inhibitor. In both of these studies, it was found that the use of β‐blockers resulted in a reduced mortality rate, suggesting the possibility that β‐blockers may have effects beyond rate control. However, for reasons such as the fact that the evidence supporting the usefulness of β‐blockers in septic shock is still somewhat controversial,^127^ this guideline does not include a CQ regarding their use.

The recommendations and expert consensus concerning initial resuscitation and cardiovascular agents with respect to septic shock presented in this guideline are based on the RCTs and/or systematic reviews that have been reported so far. However, the treatment of sepsis can vary significantly depending on the level of care offered by a given facility and the level of knowledge and skills of the attending physician and staff. This guideline related to sepsis and septic shock should be used wisely with these things in mind. Time is a critical factor with respect to the effectiveness of initial resuscitation and cardiovascular agents in septic shock, and it is important to fully understand that “sepsis is an emergency” and to treat patients with septic shock promptly.

### CQ7‐1: Is EGDT recommended for initial resuscitation in patients with sepsis or septic shock?

#### Answer (Recommendation)

We suggest against the use of EGDT when performing initial resuscitation in patients with sepsis or septic shock (2A; rate of agreement: 100%).

#### Rationale

Three RCTs^110^, ^111^, ^112^ conforming to the PICO process were identified based on a search of the PubMed database and were used in the final analysis for this CQ. Regarding the 90‐ and 28‐day mortality rates, EGDT was not effective in improving mortality rate in comparison to the standard treatment [90‐day mortality rate: risk ratio: 0.98 (95% confidence interval [CI]: 0.88–1.10); 28‐day mortality rate: risk ratio: 0.98 (95% CI: 0.84–1.13)]. The time to shock reversal was not assessed by any RCT. Regarding intensive care unit (ICU) length of stay, the mean difference (MD) was 0.27 (95% CI: −0.33 to 0.87) in the comparison between the EGDT group and the standard treatment group, and no significant difference was observed.

Regarding the benefit‐risk balance, no improvement in mortality rate as a result of complying with EGDT was observed in comparison to the standard treatment. In addition, no shortening of ICU length of stay as a result of complying with EGDT was observed [MD: 0.27 (95% CI: −0.33 to 0.87)], and no benefit of EGDT over the standard treatment could be found. However, dobutamine dosages and the quantity of blood transfused increased significantly in the EGDT group,^110^, ^111^ and due to the increased frequency of arrhythmias associated with dobutamine, greater overall risk of side effects associated with transfusions, and increased time and quantity of work required of hospital staff, it is possible that compliance with EGDT may increase the risk of harm (burden) faced by patients. Based on the above, it was determined that the potential harms presented by EGDT likely outweigh its potential benefits.

### CQ7‐2: What volume of fluid should be given in initial resuscitation in septic shock?

#### Answer (Opinion)

We suggest that 30 mL/kg or more of an extracellular fluid replacement solution is administrated when performing initial fluid resuscitation in patients with septic shock with a relative decrease in intravascular volume associated with vasodilatation (expert consensus/no evidence; rate of agreement: 100%).

Comment: 30 mL/kg or more of an extracellular fluid replacement solution should be administered after assessing the decrease in intravascular volume.

#### Rationale

No RCTs applicable to this CQ could be found as a result of a search of the PubMed database. As such, it was concluded that an expert consensus should be offered, as the evidence for this CQ is inadequate to support a recommendation. In addition, in three large‐scale RCTs evaluating the effectiveness of EGDT (ProCESS,^110^ ARISE,^111^ and ProMISe^112^), when the differences in intergroup (EGDT group versus standard treatment group) total volume of fluid transfused prior to study protocol initiation were calculated, the following differentials were revealed: ProCESS (2.3 ± 1.5 L versus 2.1 ± 1.4 L), ARISE (2.5 ± 1.2 L versus 2.6 ± 1.3 L), and ProMISe (1.9 ± 1.1 L versus 2.0 ± 1.1 L). All the subjects had already received over 30 mL/kg of crystalloid solution during the initial resuscitation prior to group assignment.

Regarding the benefit‐risk balance, the concept of large‐volume initial fluid resuscitation (infusion of 30 mL/kg or 2,000 mL within ~1 h) became recognized as a common sense approach based on the conventional guidelines, and there is a possibility that the prognosis of patients with sepsis may be improved by supplementing the relative decrease in intravascular volume associated with vasodilatation and optimizing the balance of tissue oxygen supply and demand as quickly as possible.

On the other hand, excessive extracellular fluid replacement may cause a deterioration in cardiac function (heart failure) and pulmonary function (pulmonary edema). Frequent assessment of hemodynamics is necessary to avoid excessive volume loading, which may increase the burden on medical staff. The cost of extracellular fluid replacement solution may be a burden on the intervention group but is relatively low. Based on the above considerations, it was concluded that the benefits of administering 30 mL/kg or more of an extracellular fluid replacement solution during the initial resuscitation in septic shock clearly outweigh the potential risks.

### CQ7‐3: Should cardiac function be assessed using echocardiography when initiating fluid resuscitation in sepsis?

#### Answer (Opinion)

We suggest the cardiac function using echocardiography is assessed when initiating fluid resuscitation in patients with sepsis (expert consensus/no evidence; rate of agreement: 100%).

Comment: The assessment of cardiac function using echocardiography discussed in this CQ indicates the simple evaluation of cardiac function performed at the bedside. It is focused on cardiac function (movement of the heart), and measurements related to vascular (inferior vena cava diameter, intracardiac volume) intended to afford an approximate assessment of intravascular volume prior to initiating resuscitation. It is desirable that all physicians, and not just cardiologists, involved in the resuscitation of patients with sepsis be proficient with this technique.

#### Rationale

Although a literature search was conducted to identify RCTs examining whether the assessment of cardiac function using echocardiography affects the prognosis of patients with sepsis undergoing initial resuscitation, no RCTs pertaining to this CQ could be found. Therefore, it was concluded that an expert consensus should be offered as the evidence for this CQ is inadequate to support a recommendation.

Regarding the benefit‐risk balance, although no supporting evidence could be found, assessing cardiac function and intravascular volume using echocardiography when initiating resuscitation in patients with sepsis is useful in determining the infusion rate and in catecholamine selection. Therefore, it is believed that conducting this assessment will lead to more appropriate fluid resuscitation and drug administration. Echocardiography assessment is simple and non‐invasive, and little patient burden for physicians is associated with the intervention itself. In institutions that do not routinely use echocardiography for assessment, the use of this technique will require additional time and may contribute to delays in initiating resuscitation. In addition, the price of most echocardiography devices is in the range of several million yen (approximately USD 77,000), and thus the financial burden placed on facilities will be substantial when purchasing a new device. However, such devices have high versatility, are believed to be adequate for their desired uses, and cost‐effective. Therefore, it was concluded that the benefits of assessing cardiac function using echocardiography when initiating resuscitation in patients with sepsis clearly outweigh the potential harms.

### CQ7‐4: Should a crystalloid solution or an artificial colloidal solution be used in the initial fluid resuscitation?

#### Answer (Recommendation)

We suggest against the use of an artificial colloidal solution during the initial resuscitation of patients with sepsis or septic shock (2B; rate of agreement: 89.5%).

#### Rationale

Nine RCTs^128^, ^129^, ^130^, ^131^, ^132^, ^133^, ^134^, ^135^, ^136^ were identified as a result of the systematic review^137^ conducted for this CQ. The effect of infusing an artificial colloidal solution on the risk ratios for the different mortality rates examined were as follows: ICU mortality rate: 0.56 (95% CI: 0.34–0.94), 28‐day mortality rate: 1.11 (95% CI: 0.96–1.28), 90‐day mortality rate: 1.14 (95% CI: 1.04–1.26). The impact on other risk ratios examined was as follows: acute kidney injury (AKI) incidence risk ratio: 1.32 (95% CI: 1.09–1.60), renal replacement therapy (RRT) performance risk ratio: 1.46 (95% CI: 1.21–1.77), red blood cell (RBC) transfusion risk ratio: 1.19 (95% CI: 1.04–1.36), fresh frozen plasma transfusion risk ratio: 1.18 (95% CI: 0.94–1.49). Although ICU mortality rate decreased because of artificial colloidal solution use, the 90‐day mortality rate, AKI incidence rate, RRT performance rate, and the RBC transfusion rate each increased significantly.

Regarding the benefit‐risk balance, it is difficult to determine whether mortality rate will improve by using an artificial colloidal solution during the initial fluid resuscitation, as the AKI incidence risk ratio, the RRT performance risk ratio, and the RBC transfusion risk ratio each increased significantly. The cost of artificial colloidal solutions is higher than crystalloid solutions and may cause allergies, which can place an additional burden on the intervention group. Based on the above, it was determined that the potential harms associated with the use of artificial colloidal solution likely outweigh the potential benefits.

### CQ7‐5: Should albumin solution be used during the initial resuscitation in septic shock?

#### Answer (Recommendation and opinion)

We suggest against the routine use of albumin solution during the initial resuscitation of patients with sepsis (2C).

The administration of albumin solution may be considered when large volumes of crystalloid solution are required for resuscitation, or when hypoalbuminemia is observed (expert consensus/no evidence; rate of agreement: 94.7%).

#### Rationale

A search of the PubMed database was conducted using the keywords “sepsis,” “septic shock,” and “albumin.” Five systematic reviews and one new RCT (CRISTAL [Colloids Versus Crystalloids for the Resuscitation of the Critically Ill] trial^138^) were extracted. The systematic reviews^139^ and RCT^138^ found using the most recent literature search period and that scored highly on the AMSTAR (A Measurement Tool to Assess Systematic Reviews) measurement tool (9 points) were adopted as high‐quality studies for this CQ.

Among these studies, only the SAFE (Saline Versus Albumin Fluid Evaluation) 2011 study^140^ was identified as an applicable RCT. No significant difference between the mortality risk ratio of 0.87 (95% CI: 0.74–1.02) and ICU length of stay of 0.7 (95% CI: −0.10 to 1.50) was observed. No assessment was carried out regarding time to shock reversal.

Regarding the benefit‐risk balance, although there was a tendency for a decrease in mortality rate, no significant difference was observed between the albumin and control groups. In addition, several complications, including infection and allergic reactions, may occur following albumin administration. Based on these findings, the potential risks for the albumin use as a standard resuscitation fluid likely outweighed the potential benefits.

### CQ7‐6: What method should be used to predict fluid responsiveness during initial fluid resuscitation?

#### Answer (Opinion)

We suggest the combination of multiple monitoring methods is used while considering the limitations of each indicator, for predicting fluid responsiveness during initial fluid resuscitation in patients with sepsis and septic shock (expert consensus/quality of evidence “C” ; rate of agreement: 94.7%).

Comment: The evidence was insufficient to support the recommendation of specific monitoring techniques to be used during initial resuscitation in patients with sepsis and septic shock.

#### Rationale

270 reports were identified as a result of a search for studies assessing survival in sepsis and septic shock patients who had undergone initial fluid resuscitation using various monitoring methods. After primary and secondary screening, five RCTs were extracted and used in the analysis.^141^, ^142^, ^143^, ^144^, ^145^ Four RCTs included evaluation of PLR, one for transpulmonary thermodilution, and two for SVV; a meta‐analysis was performed for each evaluation method.

No significant effect on the defined outcomes for analysis (mortality rate, ICU length of stay, time to shock reversal) was observed for these three evaluation methods. The monitoring methods used in the control group also varied and “performance of initial resuscitation without use of a specific monitoring method” established as the control for PICO (C) was not adopted. Therefore, it was determined that a serious issue regarding indirectness existed. Because of the difficulty in implementing study blinding, small sample size, the risk of bias, and various inaccuracies, the quality of the study was lowered, and the strength of this evidence was classified as weak (C) or very weak (D). Based on these findings, it was decided that the current evidence was not sufficient to support a recommendation and that an opinion (expert consensus) would be presented.

Regarding the benefit‐risk balance, central venous catheters and arterial catheters are indwelled in most cases, and the use of some form of monitoring and optimization of infusion volume can result in improved prognosis. Therefore, it was concluded that the benefits of predicting fluid responsiveness during initial resuscitation likely outweigh the potential harms.

### CQ7‐7: Should lactate levels be used as an indicator during initial resuscitation in sepsis?

#### Answer (Opinion)

We suggest that lactate levels over time are used when performing initial resuscitation in patients with sepsis (expert consensus/no evidence; rate of agreement: 94.7%).

#### Rationale

Primary and secondary screening of literature (174 sources) obtained after a search was conducted. As no RCTs applicable to this CQ (comparing lactate levels over time during initial resuscitation in sepsis) could be found, it was decided that an expert consensus would be presented instead of a recommendation.

Regarding the benefit‐risk balance, lactate levels are associated with the patient's prognosis in sepsis, and measuring lactate levels can aid in identifying critically ill patients. In addition, according to a report by Jansen *et al*., as a result of comparing patients with a lactate level of 3.0 mEq/L or higher (proportion of patients with sepsis was approximately 40% in both groups) to a comparator group that underwent initial therapy with lactate clearance as an indicator, no significant difference in in‐hospital mortality rate was observed in a univariate analysis. However, in a multivariate analysis, in‐hospital mortality rate improved in the group in which lactate clearance was used as an indicator.^146^ Therefore, performing initial resuscitation while monitoring and assessing lactate levels over time may improve patient's prognosis in sepsis. Arterial punctures and invasive arterial catheter insertion may also cause mechanical complications such as hematoma and embolism as well as infection, and in order to confirm lactate clearance, frequently measuring and analyzing blood gas content during initial resuscitation becomes necessary. However, as monitoring through an arterial catheter is believed to be performed in many patients; the quantity of blood collected per measurement is small, and the burden on patients appears to be minimal. Therefore, it was concluded that the potential benefits of the use of lactate levels as an indicator during initial resuscitation clearly outweigh the potential harms.

### CQ7‐8: ScvO_2_ or lactate clearance: which is more useful as an indicator of initial resuscitation?

#### Answer (Opinion)

Either ScvO_2_ or lactate clearance may be used as indicators of initial resuscitation (expert consensus/quality of evidence “D”; rate of agreement: 94.7%).

#### Rationale

Only one RCT (Jones *et al*.^118^) comparing ScvO_2_ and serum lactate values was identified, and there was no significant difference in in‐hospital mortality rates between using ScvO_2_ and lactate clearance to guide initial resuscitation.

Regarding the benefit‐risk balance, a specific central venous catheter is needed for continuous ScvO_2_ monitoring. Collecting blood samples to evaluate ScvO_2_ or lactate levels may increase the workload on physicians and medical staff, as well as the risk of infection. However, the potential benefits were determined to outweigh the potential harms since both measurements enable the assessment of oxygen transport capacity in tissues.

### CQ7‐9: Noradrenaline or dopamine: which should be used as a first‐line vasopressor to treat patients with septic shock that are unresponsive to initial fluid resuscitation?

#### Answer (Recommendation)

We recommend the use of noradrenaline as a first‐line vasopressor to treat patients with septic shock that is unresponsive to initial resuscitation (1B; rate of agreement: 100%).

#### Rationale

The systematic review reported by Avni *et al*.,^147^ was adopted as evidence as it had the highest quality. A total of 14 RCTs comparing noradrenaline and dopamine were identified, but these studies did not examine the time required to recover from septic shock. Noradrenaline administration significantly improved the 28‐day mortality rate in comparison to dopamine (risk ratio: 0.89 [95% CI: 0.81–0.98]). Regarding the ICU length of stay, the MD was 1.01 (95% CI: −0.65 to 2.66) as a result of the comparison between noradrenaline and other vasopressors, and no significant differences were observed. Regarding the incidence of complications, noradrenaline use resulted in a significantly lower incidence of complications compared to dopamine (risk ratio: 0.34 [95% CI: 0.14–0.84]).

Regarding the benefit‐risk balance, noradrenaline use resulted in a significant improvement in 28‐day mortality rate in comparison to dopamine, and the frequency of harmful complications (fatal arrhythmias, myocardial/cerebral/upper or lower limb ischemia/infarction, etc.) was significantly lower. Therefore, it was concluded that the potential benefits of noradrenaline use to treat patients with septic shock clearly outweighed the potential harms.

### CQ7‐10: Should adrenaline be used in septic shock when noradrenaline fails to improve the blood pressure?

#### Answer (Opinion)

We suggest that adrenaline is used in cases in which the maintenance of hemodynamic status is insufficient despite appropriate fluid resuscitation and noradrenaline administration (expert consensus/no evidence; rate of agreement: 100%).

#### Rationale

A literature search yielded 365 reports evaluating the effects of adrenaline in septic shock when noradrenaline fails to achieve a target blood pressure, and of these, eight were identified through the primary screening. No RCTs conforming to the PICO process for this CQ were found.

Regarding the benefit‐risk balance, no RCT was examined for this CQ. Adrenaline as a first‐line vasopressor has not shown significant improvements in mortality rates in comparison with noradrenaline. However, 20%–40% of cases of septic shock are associated with SIMD which predicts worse outcomes,^148^ and it has been suggested that the administration of adrenaline may improve cardiac function in cases complicated by SIMD.^149^ Although adrenaline use is associated with side effects such as tachycardia, decreased tissue perfusion, and lactate acidosis, no study has shown worse outcomes following adrenaline administration. It was concluded that the potential benefits of adrenaline use when noradrenaline fails to achieve target blood pressure clearly outweighed the potential harms.

### CQ7‐11: Should vasopressin be used in patients with septic shock who fail to achieve target blood pressure despite the use of noradrenaline?

#### Answer (Opinion)

We suggest that vasopressin is used in patients with septic shock who show evidence of persistent hypotension despite adequate fluid resuscitation and the use of noradrenaline (expert consensus/quality of evidence “B” ; rate of agreement: 100%).

#### Rationale

A literature search yielded 365 records, and two RCTs^150^, ^151^ were extracted for the meta‐analysis after primary and secondary screening. ICU length of stay, 28‐day mortality rate, and complication rate were assessed in these two RCTs, but time to shock reversal was not assessed. The risk ratios for 28‐day mortality rate and complication rate were 0.90 (95% CI: 0.76–1.07) and 0.73 (95% CI: 0.24–2.23), respectively. The MD in ICU length of stay was ‐ 0.95 days (95% CI: −1.73 to −0.17).

In the two RCTs,^150^, ^151^ noradrenaline or noradrenaline plus vasopressin was administered when vasopressors were required to maintain target blood pressure despite adequate fluid resuscitation. Compared to using noradrenaline alone, the evidence of this systematic review and meta‐analysis showed that adding vasopressin decreased the ICU length of stay by one day, but no difference was observed regarding 28‐day mortality rate. In addition, no difference was observed in complication rate between the two groups. Based on these findings, it was concluded that the benefits of adding vasopressin when using noradrenaline alone fails to achieve target blood pressure likely outweigh the harms of adding it.

### CQ7‐12: Should dobutamine be used in patients with septic shock who show evidence of cardiac dysfunction?

#### Answer (Opinion)

We suggest that dobutamine is used in septic shock when cardiac function remains diminished, and maintenance of hemodynamics is insufficient despite adequate fluid resuscitation and noradrenaline administration (expert consensus/quality of evidence “C” ; rate of agreement: 94.7%).

#### Rationale

Two RCTs^122^, ^123^ were identified involving patients with septic shock in whom blood pressure could not be maintained with adequate fluid resuscitation and noradrenaline administration, and cardiac function was normal or decreased. Adrenaline was administered to the control group. The risk ratio with respect to 28‐day mortality rate was 0.88 (95% CI: 0.69–1.13), and the incidence rate of complications was 0.87 (95% CI: 0.62–1.22). The MD for the time to shock reversal and ICU length of stay were −1.00 day (95% CI: −1.89 to 0.11) and 1.00 day (95% CI: 0.33–1.67), respectively.

Regarding the benefit‐risk balance, although the superiority of dobutamine versus adrenaline is not recognized, the 28‐day mortality rate remained at approximately 40% in the RCT including patients predicted to have a very high risk of death, and as such there appears to be some benefit in administering dobutamine. There were also no differences observed regarding the frequency of complications such as arrhythmia in comparison to the patients that received adrenaline. Based on the above observations, it was determined that the benefits of administering dobutamine likely outweigh the potential harms.

## CQ8: CORTICOSTEROID THERAPY FOR SEPTIC SHOCK

### Introduction

Cortisol is produced depending on the physiological state of the body. Its production and secretion are increased in response to various invasive insults for maintaining homeostasis, and is thus recognized as the “stress hormone.” Corticosteroids have been used as an adjunctive treatment for shock, since circulatory shock often develops in cortisol‐deficient patients, such as in Addison's disease and acute adrenal insufficiency.

In septic shock, apart from the insufficient cortisol secretion (relative adrenal insufficiency), a reduction in glucocorticoid receptor expression and their diminished responsiveness are also observed, which may lead to the so‐called “critical illness‐related corticosteroid insufficiency (CIRCI)”.^152^ The administration of steroids emerged as a treatment option suited to this particular pathophysiology and was adopted into the Surviving Sepsis Campaign Guidelines (SSCG) 2004,^153^ according to the concept of “relative adrenal insufficiency.” Since then, however, the adrenocorticotropic hormone (ACTH) stimulation test was proved not to be useful in identifying corticosteroid‐responsive patients, partly because the concentration of free cortisol, actually present *in vivo*, could not be measured or estimated by a measurement of total cortisol concentration, and therefore the rapid ACTH stimulation test was “not recommended” (class 2B) in SSCG 2008.^154^ In studies assessing the effect of low‐dose corticosteroids in patients with sepsis of various severities, their effectiveness was observed only in critically‐ill patients with septic shock.^155^, ^156^ In 2016, Keh *et al*. showed that the administration of corticosteroids to patients with severe sepsis but without septic shock, did not reduce the incidence of shock and mortality rate by a randomized clinical trial (RCT; the HYPRESS [Hydrocortisone for Prevention of Septic Shock] Randomized Clinical Trial).^157^ Based on these findings, the use of corticosteroids is not recommended for septic patients, who are not in shock or who have recovered from shock following initial fluid resuscitation and administration of vasopressors. Currently, low‐dose corticosteroid therapy is indicated in adult septic patients who are not responsive to initial fluid resuscitation and remain in shock (systolic blood pressure 90 mmHg or less) for more than 1 h regardless of administration of high‐dose catecholamines.

In addition to its effects as supplementary therapy, corticosteroids downregulate the production of inflammatory cytokines by inhibiting the nuclear translocation of NFκB and promote the recovery of catecholamine receptor function.

Corticosteroids had been given to patients with septic shock since the 1940s, and there was a time when this approach received most clinicians’ and researchers’ attention. However, in 1987, Bone *et al*. showed that a high‐dose (also referred to as “pharmacological dose”) corticosteroid regimen (methylprednisolone [MPSL] 30 mg/kg × 4/day) did not decrease the mortality rate, but increased the incidence of adverse events, such as gastrointestinal bleeding and hyperglycemia in an RCT.^158^, ^159^ Thus, after 2000, low‐dose (also referred to as “stress dose”) corticosteroid therapy (hydrocortisone (HC) 200–300 mg/day) has become mainstream. Although improvement in the proportion of shock reversal and shortening of the time to shock reversal have been observed, conflicting results have been reported with regard to mortality rate. In 2004, Annane *et al*. published a meta‐analysis, including their previous RCT (French study),^156^ which showed that low‐dose corticosteroids significantly decreased mortality rate, in addition to an improvement in the proportion of shock reversal, shortening of vasopressor therapy periods, and without an increase in adverse events. In contrast, an RCT, the Corticosteroid Therapy of Septic Shock (CORTICUS) study, with a sample size of 500, reported no improvement in 28‐day mortality rate and an increase in the incidence of complications, including infection, hyperglycemia, and hypernatremia in 2008.^157^ There are criticisms that the severity of the condition of patients enrolled into the CORTICUS study was lower, while the timing of initial corticosteroid therapy was later than those of the patients enrolled into the French study.

During the long history of corticosteroid therapy for sepsis, there have also been changes in the definition, usual care, and the kind of corticosteroid used in sepsis. The definitions for sepsis, severe sepsis, and septic shock were proposed in 1992, and the usage of corticosteroid has changed substantially from a high dosage to a lower dosage since 2000. Usual care for patients with sepsis has been standardized after the initiation of Surviving Sepsis Campaign in 2004. Thus, we decided to assess low‐dose corticosteroid therapy by searching for RCTs conducted on septic shock after 2004. The first clinical question (CQ) on this topic is whether we should use low‐dose corticosteroid (HC) to treat adult patients with septic shock (e.g., patients who are unresponsive to initial fluid resuscitation and exhibit a systolic blood pressure of 90 mmHg or lower for more than 1 h regardless of the administration of high dose vasopressors).^160^, ^161^ The next practice‐oriented CQs addresses the three questions: “when should we administer corticosteroids?”, “what are the optimal dosage and administration period?” and “should we use HC among commercially available corticosteroids?”

The largest‐scale double blinded RCT is currently underway in Australia, New Zealand and Europe (ADjunctive coRticosteroid trEatment iN criticAlly iLl patients with septic shock (ADRENAL)) trial by Australian and New Zealand Intensive Care Society (ANZICS), which evaluated the 90‐day mortality rate following low‐dose corticosteroid therapy (continuous intravenous administration of hydrocortisone 200 mg/day for 7 days). It is supposed to recruit 3800 patients with septic shock, and its results are being awaited.

### CQ8‐1: Should we use low‐dose corticosteroids (HC) for adult patients with septic shock who are not responsive to initial fluid resuscitation and vasopressors?

#### Answer (recommendation)

Corticosteroids should not be administered if patients recover from septic shock with adequate fluid resuscitation and vasopressor therapy. We suggest that a low‐dose corticosteroid (HC) is administered to promote recovery from shock in adult patients with septic shock that is not responsive to the initial fluid resuscitation and vasopressors (2B; rate of agreement: 94.7%).

#### Rationale

We searched for references published in 2004 or later, when the standardized care for sepsis was introduced by SSCG 2004 after being defined in 1992 as a systemic inflammatory response syndrome (SIRS) accompanying infection, with the keywords “septic shock” and “low‐dose steroid,” and identified eight RCTs (Bollaert *et al*.,^162^ Briegel *et al*.,^163^ Chawla *et al*.,^164^ Annane *et al*.,^165^ Oppert *et al*.,^166^ Mussack *et al*.,^167^ Sprung *et al*.,^156^ and Arabi^168^). As these eight studies were all included in the meta‐analysis by Wang *et al*.,^160^ we initially decided to use it for this CQ. However, after a second search with the same keywords at the end of December 2015, one new report by Gordon *et al*.^161^ was identified, and we thus performed a new meta‐analysis including this study.

There were nine and six RCTs studying 28‐day mortality rate and the proportion of shock reversal by day 7, respectively. With regards to adverse events, six RCTs analyzed infection, six assessed gastrointestinal bleeding, and three assessed hyperglycemia. There were no problems regarding a risk of bias, inconsistency, or indirectness in the analysis of 28‐day mortality rate, the proportion of shock reversal by day 7, and complication rates. However, the confidence intervals (CIs) for the two complications (infections, gastrointestinal bleeding) were wide, susceptible to inaccuracy, and their evidence levels were thus lowered by one. The risk ratios (RRs) for 28‐day mortality rate and the proportion of shock reversal by day 7 were 0.96 (95% CI: 0.81–1.13) and 1.32 (95% CI: 1.19–1.46), respectively. The RRs for infection, gastrointestinal bleeding, and hyperglycemia were 1.09 (95% CI: 0.88–1.35), 1.35 (95% CI: 0.85–2.13), and 1.15 (95% CI: 1.07–1.25), respectively.

Although the rates of adverse events increased or tended to increase, the proportion of shock reversal by day 7 increased significantly, and 28‐day mortality rate tended to decrease; thus we judged that the benefits likely outweighed the harms.

### CQ8‐2: Should we administer corticosteroids earlier or later for adult patients with septic shock who are not responsive to initial fluid resuscitation and vasopressors?

#### Answer (opinion)

We suggest that corticosteroids are administered within 6 h after the onset of septic shock to treat adult patients with septic shock that is not responsive to the initial fluid resuscitation and vasopressors (expert consensus/no evidence; rate of agreement: 94.7%).

#### Rationale

No RCTs comparing whether the therapeutic effects and side effects of low‐dose corticosteroids in adult patients with septic shock differ depending on the timing of administration (early initiation versus late initiation) could be found. The French study involving the administration of corticosteroids within 8 h after onset of shock^165^ has demonstrated a superior 28‐day mortality rate as well as improved the proportion of shock reversal compared to the CORTICUS study^156^ involving corticosteroid administration within 72 h after the onset of shock. Two observational studies have recently reported efficiencies in the timing of administering corticosteroids to treat septic shock. In 2012, Park *et al*.^169^ conducted a retrospective study using a time‐dependent Cox regression model to assess the administration of corticosteroids in patients with septic shock (178 cases). As a result, it was found that the 28‐day mortality rate was significantly lower in the early group that received corticosteroids within 6 h of onset of shock compared to the late group that received steroids after 6 h or more from the onset of shock (51% versus 32%, RR 0.63; 95% CI: 0.42–0.93, *P *=* *0.002). According to the prospective study (170 cases) conducted by Katsenos *et al*.^170^ in 2014, inotropes may be discontinued earlier in patients with early initiation of HC (<9 hrs after inotropes) in comparison to patients with late initiation of HC (>9 hrs after inotropes; log‐rank: 18.248, *P *=* *0.000019), and 28‐day mortality rate also declined (52.2% versus 30.6%; Fisher's exact test, *P *=* *0.012). Based on the above findings, the early administration of corticosteroids within 6 h of the onset of shock is recommended when steroids are to be administered for the treatment of septic shock.

Regarding the benefit‐risk balance, early steroid administration for the treatment of septic shock can be expected to promote shock reversal, resulting in the prevention of irreversible organ failure caused by prolonged hemodynamic derangement and to reduce the mortality rate. There have been no reports concerning complications arising due to the timing of administration, and no increased burden on medical staff associated with the timing of corticosteroids administration (early initiation, late initiation) is anticipated. However, since no RCTs conforming to the Patients, Intervention, Comparison, Outcome (PICO) process could be found, it was determined that the benefit‐risk balance is still unknown.

### CQ8‐3: What are the optimal dose and administration period when administering corticosteroids?

#### Answer (opinion)

We suggest that 300 mg/day or less of HC for a maximum period of approximately 7 days is used when steroids are required for patients with septic shock (expert consensus/no evidence; rate of agreement: 94.7%).

#### Rationale

No RCTs examining and comparing whether the therapeutic effects and side effects of corticosteroids administered to adult patients with septic shock differ depending on the dosage and administration period could be found. It was concluded that high‐dose steroid administration, which had been practiced until the 1990s, was ineffective or even harmful based on the results of two RCTs and one meta‐analysis.^158^, ^159^ Low‐dose, long‐term administration of HC was practiced in the 2000s, and, improvements in mortality rates were also reported in addition to improvements in the proportion of and time to shock reversal. In large‐scale RCTs by Annane *et al*.^165^ and Sprung *et al*.,^156^ faster shock reversal was observed in both studies as a result of administering HC 200 mg/day in four divided doses, although the 28‐day mortality rate was significantly lower in the Annane study than the Sprung study. In a meta‐analysis by Annane *et al*.^171^ assessing 17 RCTs, HC administration methods were divided into four according to the high‐dose/low‐dose (with a 300 mg/day limit) and long‐term/short‐term (with a 5‐day limit) and examined. Improvements in both the proportion of shock reversal and the 28‐day mortality rate were observed only in the low‐dose/long‐term administration groups. Also, in the meta‐analysis of the latest Cochrane Review of dose and duration of treatment among the steroid administration groups by Annane *et al*.,^172^ treatment with a long course of low‐dose corticosteroids (at least 5 days and 300 mg/day or less) significantly reduced 28‐day mortality rate (RR 0.87, 95% CI: 0.78–0.97), but did not improve in the high‐dose/short‐term administration groups.

While several reports recommend continuous infusion at 10 mg/hr after intravenous infusion of 100 mg is given for the management of blood glucose,^152^ the utility of continuous intravenous infusion of steroids with long half‐lives is not clear. The period of administration is not fixed at 5 days, and administration of steroids for a long time should be avoided. When discontinuing steroid therapy, gradually tapering off the dosage is safer than sudden discontinuation from the viewpoints for maintenance of hemodynamics and prevention of rebounds in immune function.

As mentioned above, administration of steroids at dosages not exceeding the 300 mg/day equivalent of HC is recommended to promote the proportion of shock reversal in patients with septic shock (over a maximum period of ~7 days).

Regarding the benefit‐risk balance, while low‐dose long‐term steroids that are administered until the time to shock reversal are expected to increase proportions of shock reversal and decrease the frequency of mortalities. On the other hand, high‐dose short‐term steroid administration is associated with a greater frequency of hyperglycemia and gastrointestinal bleeding resulting in worsened prognosis compared with the non‐administration group. Although low‐dose/long‐term steroids did not increase the frequency of complications after an overall assessment of low‐dose long‐term steroids, it is necessary to pay sufficient attention to the risk of deterioration of long‐term prognosis due to hyperglycemia, gastrointestinal bleeding, and increased risk of infection. However, no RCTs conforming to the PICO process could be found, and it was determined that the benefit‐risk balance is still unknown.

### CQ8‐4: Should hydrocortisone be administered?

#### Answer (opinion)

We suggest that HC or MPSL is administered to treat patients with septic shock (expert consensus/no evidence; rate of agreement: 100%).

#### Rationale

There were no RCTs comparing the therapeutic effects and side effects of different steroids administered in adult patients with septic shock. Glucocorticoids resulted in an improvement in the proportion of shock reversal and reduced the 28‐day mortality rate in adult patients with septic shock. Although HC, a pharmacologic form of physiological cortisol, is most commonly used in large‐scale RCTs, it is a short‐acting steroid and has both glucocorticoid and mineralocorticoid effects. In addition, Meduri *et al*.^173^ administered 1 mg/kg of MPSL, an intermediate‐acting steroid with no mineralocorticoid effects, and then continued MPSL administration at 1 mg/kg/day for 14 days to patients with septic shock in the same way as is given to patients with acute respiratory distress syndrome (ARDS). MPSL is five times more potent than HC in terms of glucocorticoid activity, and its half‐life is 1.3 times that of HC. However, the actual dosage of MPSL is approximately half of that of HC.^174^ In a retrospective observational study^175^ (involving HC: 21 patients: 50 mg × 4/day; MPSL: 19 patients: 20 mg × 2/day) comparing HC and MPSL with bioequivalent doses, no difference in 28‐day mortality rate, the proportion of shock reversal, or incidence of complications were observed. We do not recommend combining fludrocortisone and HC. In an RCT assessing the combination of fludrocortisone and HC,^165^ there was no additional benefit associated with adding fludrocortisone to HC, and adding fludrocortisone increased the infection rate, especially for urinary tract infections. Dexamethasone which has greater glucocorticoid activity and a longer half‐life should not be administered due to its immediate and prolonged suppressive effects on the hypothalamic‐pituitary‐adrenal axis.^163^ Based on the above findings, we suggest using HC or methylprednisolone MPSL in patients with septic shock.

Regarding the benefit‐risk balance, even if HC, or alternatively, MPSL is administered, although there was no significant difference between the groups with respect to the incidence of complications, it is necessary to pay sufficient attention to the risk of deterioration of long‐term prognosis due to hyperglycemia, gastrointestinal bleeding, and increased risk of infection. However, no RCTs conforming to the PICO process could be found, and it was determined that the benefit‐risk balance pertaining to patients in either group is still unknown.

## CQ9: BLOOD TRANSFUSION PREPARATIONS

### Introduction

The treatment of sepsis in Japan involves the use of blood component preparations (red blood cell concentrate, fresh‐frozen plasma, and platelet concentrate) as well as plasma fraction preparations (albumin preparations, immunoglobulin preparations, and antithrombin preparations). Among these blood products, usage standards for blood component preparations and albumin preparations from plasma fraction preparations have been formulated in accordance with the “Guidelines for Blood Product Use” (2012 revision) established by the Ministry of Health, Labour and Welfare,^176^ from the perspective of limited medical resources regarding blood donations as well as the risk of side effects associated with the use of human blood. The use of these products is recommended during medical treatment covered under Japan's National Health Insurance system as well based on these standards. However, whether the Guidelines for Blood Product Use are also valid in the context of sepsis has not yet been established, and there are also some who believe that blood products should be actively administered to address coagulopathies and hypoalbuminemia occurring in sepsis cases. On that basis, this guideline discusses the use of appropriate blood products in the management of sepsis and presents several clinical questions (CQs) addressing key questions.

In the Japanese Guideline for the Management of Sepsis (First Edition),^2^ “blood component preparations” is not an independent item, but rather red blood cell transfusions are discussed in conjunction with “initial resuscitation,” and fresh‐frozen plasma and platelet concentrate are discussed in the context of “disseminated intravascular coagulopathy (DIC).” In addition, of the various plasma fraction preparations, albumin preparations are discussed alongside “initial resuscitation,” “immunoglobulin preparations” are addressed as a separate entry, and antithrombin preparations are discussed alongside “DIC.” Although plasma fraction preparations are considered in a similar context as the first edition, it was decided that blood component preparations would be addressed as a separate “blood transfusions” entry, as the guideline would be created based on evidence related to sepsis and the Ministry of Health, Labour and Welfare's Guidelines for Blood Product Use.

The “blood transfusions” team designed a CQ to address each type of blood component preparation (red blood cell concentrate, fresh‐frozen plasma, and platelet concentrate). However, the recommendation that red blood cell transfusions be performed after patients have recovered from shock, are hemodynamically stable, and when hemoglobin values fall below 7 g/dL is present in both the Japanese Guideline for the Management of Sepsis (First Edition) and the Surviving Sepsis Campaign Guidelines (SSCG) 2012,^29^ and is also not inconsistent with the Guidelines for Blood Product Use established by the Ministry of Health, Labour and Welfare. In contrast, the SSCG 2012 describes red blood cell transfusion with a target hematocrit value of 30% as one means of maintaining oxygen supply to tissues when performing initial resuscitation in septic shock cases, and the debate on this subject is ongoing. Accordingly, in this guideline, it was determined that no consensus could be reached regarding administering red blood cell transfusions to patients with stable hemodynamics. Thus CQ9‐1 was formulated to focus on the topic of red blood cell transfusions during initial resuscitation of septic shock patients and addresses the question “When should red blood cell transfusion be initiated when performing initial resuscitation in septic shock cases?” In addition, discussion of the appropriate performance of transfusions of fresh‐frozen plasma and platelet concentrate in coagulation factor supplementation, when surgical intervention is needed, or in the event of hemorrhaging was determined to be necessary, and accordingly CQ9‐2 and CQ9‐3 address the questions of “Should fresh‐frozen plasma be used in sepsis cases?” and “Should platelet transfusions be performed in sepsis cases?”, respectively.

### CQ 9‐1: When should red blood cell transfusion be initiated when performing initial resuscitation in septic shock cases?

#### Answer (Recommendation)

We recommend initiating red blood cell transfusion during initial resuscitation in septic shock cases at hemoglobin levels of ≤7 g/dL (1B; rate of agreement: 94.7%).

Comment: This CQ addresses red blood cell transfusions during initial resuscitation in septic shock cases, and does not address transfusions performed after the patient is hemodynamically stable.

#### Rationale

Although the SSCG 2012^29^ proposed performing red blood cell transfusions with a target hematocrit of 30% or higher while considering the risks of hypoxia and myocardial damage during septic shock, the debate is currently ongoing. As such, an analysis of the timing of initiation of red blood cell transfusions during initial resuscitation in septic shock cases was conducted.

Two randomized controlled trials (RCTs)^177^, ^178^ conforming to the Patient, Intervention, Comparison, and Outcome (PICO) process were selected as final targets for analysis as a result of a search of the PubMed database. According to the results of a combined meta‐analysis of the control groups (Group C: 520 participants) and the intervention groups (Group I: 524 participants) of these two RCTs, the risk ratio for the 28‐day mortality rate in Group I versus Group C was 0.95 (95% confidence interval [CI]: 0.80–1.11). The incidence of ischemic complications with respect to organ damage was reported for one study only, and the risk ratio for organ damage in the control group (Group C: 489 participants) versus the intervention group (Group I: 488 participants) was 0.9 (95% CI: 0.58–1.39).

After a comparison of transfusions initiated at a hemoglobin level of ≤7 g/dL with transfusions initiated at ≤10 g/dL, no difference was observed with respect to 28‐day mortality rate and incidence of ischemic complications, and no evidence supportive of initiating blood transfusions at hemoglobin values of ≤7 g/dL or ≤10 g/dL was found. However, targeting a higher hemoglobin value requires transfusion of greater volumes of red blood cells and increases the risk of adverse effects and complications associated with transfusions, such as infection and allergic reactions. In addition, in consideration of medical economics and the fact that these kinds of blood products originate from donated blood, initiating transfusions at hemoglobin values of ≤10 g/dL should be avoided due to the risk of various adverse events, and a threshold of ≤7 g/dL is recommended instead. However, threshold hemoglobin values for initiating red blood cell transfusions may change in cases involving heart failure or ischemic heart disease as underlying diseases, and further studies are required.

### CQ 9‐2: Should fresh‐frozen plasma be used in sepsis cases?

#### Answer (Opinion)

We suggest against administration of fresh‐frozen plasma to correct coagulopathies when patients exhibit no bleeding tendencies, and no surgical intervention is required (expert consensus/no evidence; rate of agreement: 100%).

Comment: The use of fresh‐frozen plasma should be considered in keeping with the Japanese Guidelines for Blood Product Use^176^ when patients exhibit a bleeding tendency or when surgical treatment is needed.

#### Rationale

In Japan, fresh‐frozen plasma is administered to sepsis patients who exhibit bleeding tendencies or when surgical intervention is required, but may also be given to treat coagulopathies. To date, no consensus has been reached regarding the clinical utility of administering fresh‐frozen plasma to correct coagulopathies in sepsis patients. In addition, potential harms associated with fresh‐frozen plasma use include the onset of transfusion‐related acute lung injury (TRALI; frequency of fatal TRALI due to fresh‐frozen plasma use: 1:2–300,000 products^179^), among various other dangers. Thus, the use of fresh‐frozen plasma in sepsis cases was examined for this CQ.

No RCTs assessing the clinical utility of administering fresh‐frozen plasma to manage coagulopathies in sepsis patients could be found in the PubMed database. As there is currently insufficient evidence conforming to the PICO process, no recommendation could be offered for this CQ at this time, and an expert consensus is presented as an alternative.

Although there are currently no proven benefits or harm associated with the administration of fresh‐frozen plasma to manage coagulopathies in patients not exhibiting bleeding tendencies or when no surgical intervention is required, administering fresh‐frozen plasma increases patients’ risks of allergic reactions and infection accompanying blood transfusion, which can become a burden on circulation. Moreover, as of 2016, the cost of fresh‐frozen plasma was approximately 80 United States Dollar (USD)/unit (one unit of plasma (~120 mL) corresponds to 200 mL of blood). In actual practice, physicians should take note of patients’ individual views regarding donated blood products and also be aware that some patients or their family members may refuse blood transfusions for reasons such as religious beliefs.

### CQ 9‐3: Should platelet transfusions be performed in sepsis cases?

#### Answer (Opinion)

We suggest performing a platelet transfusion in sepsis cases when patients exhibit a bleeding tendency or when surgical treatment is needed, in keeping with the Japanese Guidelines for Blood Product Use^176^ (expert consensus/no evidence; rate of agreement: 100%).

#### Rationale

In Japan, platelet preparations are often administered to sepsis patients who exhibit general bleeding tendencies or when surgical intervention is needed^176^ in keeping with the Japanese Guidelines for Blood Product Use. However, there is currently no evidence upon which to base an assessment of how platelet transfusions affect the clinical course of sepsis patients. In addition, potential risks associated with platelet administration include the onset of TRALI (frequency of fatal TRALI due to platelet use: 1:3–400,000 products^179^), among various others. As such, the use of platelet preparations in sepsis patients was examined in this CQ.

No RCTs examining the clinical utility of platelet administration to sepsis patients could be found in the PubMed database. As there is currently insufficient evidence that conforms to the PICO process, no recommendation can be offered for this CQ at this time, and an expert consensus is presented as an alternative.

Although there are currently no proven benefits or harm associated with the administration of platelet preparations to patients not exhibiting bleeding tendencies or when no surgical intervention is required, platelet transfusion increases patients’ risks of allergies and infection, which can increase the circulatory burden. As of 2016, the cost of platelet preparations was approximately 720 USD/10 units (200 mL), and when administering platelets, physicians should take note of how patients’ individual views regarding donated blood products and the concept of blood transfusions can differ. Also, physicians must also be aware that some patients or their family members may refuse blood transfusions for reasons such as religious beliefs.

## CQ10: MANAGEMENT OF THE MECHANICALLY VENTILATED PATIENT

### Introduction

Sepsis‐induced respiratory system disorders occur at a high frequency and can result in hypoxemia in more severe cases, presenting as acute respiratory distress syndrome (ARDS). This condition is a typical organ system disorder in sepsis cases, and the pathology of ARDS is easy to understand if the condition is viewed as the pulmonary component of multiple organ failure. Severe sepsis is considered to be an important underlying condition together with severe pneumonia.^180^ There has also been movement in recent years towards defining severe pneumonia as sepsis of the lungs, and under such a definition approximately 80% of ARDS cases arise from sepsis.^181^, ^182^ However, the incidence of ARDS among patients with sepsis as a whole is surprisingly low, and some reports have estimated that it to be approximately 6%–7%.^183^, ^184^ Therefore, although mechanical ventilation plays an important role in the management of sepsis patients, it should be noted that there are also cases where marked deterioration in respiratory function does not occur, as well as cases where respiratory decline may be preventable through treatment intervention.

Since the establishment of the new definition of ARDS in 2012, the concept of initiating therapeutic interventions according to disease severity was introduced,^35^, ^185^ and numerous reports have been made in recent years describing the clinical utility of techniques such as oxygen therapy in mild ARDS cases, oxygen therapy administered via high‐flow nasal cannula (high‐flow nasal therapy), and noninvasive positive pressure ventilation.^186^, ^187^, ^188^, ^189^ Administering oxygen in some form to sepsis patients presenting with hypoxemia is widely practiced, and while this approach is believed to help prevent the onset of acute respiratory failure leading to ARDS, there is currently no clear supporting evidence. In addition, the ventilation strategy is of critical importance when managing sepsis patients requiring mechanical ventilation, together with the treatment of the underlying sepsis. Specifically, a “lung protective ventilation” strategy is considered to be key to reducing lung injuries, and the prevention and treatment of ventilator‐associated lung injuries (VALI) and ventilator‐associated pneumonia should also be considered once mechanical ventilation is initiated.

On this background, four clinical questions (CQs) focusing on the general management of mechanically ventilated patients were selected among the 13 CQs presented in the ARDS Clinical Practice Guidelines 2016 (ARDSGL)^190^ published by the Japanese Society of Intensive Care Medicine, the Japanese Society of Respiratory Care Medicine, and the Japanese Respiratory Society. Regarding the topics of appropriate positions for preventing complications during mechanical ventilation, ventilation in the prone position to address severe hypoxemia, and the use of muscle relaxants, it was decided that this guideline will not describe the interventions due to the need for safety instruction by specialists and are not applicable in regular wards outside the ICU. For more specialized knowledge, please refer to the ARDSGL.^190^

CQ10‐1 addresses tidal volume settings. As a result of a large‐scale, multicenter randomized controlled trial (RCT) comparing a group of ARDS patients that had a comparatively large tidal volume (12 mL/kg predicted body weight) while undergoing mechanical ventilation to another group with low tidal volume (6 mL/kg predicted body weight), the 30‐day mortality rate was significantly lower in the low tidal volume group.^191^ Based on this report, it is apparent that the concepts underlying the management of mechanically ventilated patients have changed significantly, and lung protective ventilation strategies capable of preventing VALI have been introduced into intensive care medical practice. No RCT comparing low tidal volume with conventional ventilation volume has been published since 2006. To date, no basis for establishing a target ventilation volume of 6 mL/kg predicted body weight has been demonstrated, and further study is needed.

CQ10‐2 addresses plateau pressure settings. VALI tends to occur in conjunction with decreases in lung compliance during mechanical ventilation in adult ARDS patients. Although there has been some concern regarding how VALI not only extends the period of mechanical ventilation use but can also lead to increased mortality,^192^ this increase arises from elevations in tidal volume and airway pressure during mechanical ventilation, and as such, it is expected that both can be controlled by limiting plateau pressure.^193^ However, the results of limiting plateau pressure are not all beneficial; lower plateau pressure may also lead to adverse events such as hypercapnia.^194^ Therefore, the optimum plateau pressure affording benefits without causing VALI is currently uncertain, and validation is necessary.

CQ10‐3 addresses the positive end‐expiratory pressure (PEEP) setting. It is widely known that atelectasis can be prevented and oxygenation can be improved by using PEEP. Particularly in patients with ARDS, evidence suggests that PEEP does not only help to correct hypoxemia but can also prevent further worsening of VALI by stimulating the recruitment of alveoli that have become collapsed due to inflammation or exudate.^195^, ^196^ However, the optimum PEEP value is currently unknown. Various other treatment concepts such as driving pressure, transpulmonary pressure, and electrical activity of the diaphragm have been proposed in addition to these, and future research attention is anticipated.^197^, ^198^, ^199^

Lastly, CQ10‐4 addresses the management of hydration, which is closely related to the management of sepsis. Pulmonary edema in patients with ARDS is believed to be caused by vascular endothelial damage or vascular hyperpermeability.^180^ A positive balance in transfusion volume in ARDS patients increases the frequency of mortality,^200^ and extravascular lung fluid volume is linked to disease severity and mortality rate.^201^ In contrast, relatively high‐volume fluid transfusion is recommended in guidelines for managing septic shock patients. Therefore, proper fluid management is required after patients recover from the shock state in the early stages of sepsis.

This CQ is excerpted in part from the ARDSGL.^190^

### CQ10‐1: Should a lower tidal volume be set when performing mechanical ventilation in adult patients with ARDS?

#### Answer (Recommendation)

We recommend setting the tidal volume to 6–8 mL/kg predicted body weight when performing mechanical ventilation in adult patients with ARDS (1B: excerpted from the ARDSGL).

#### Rationale

Mechanical ventilation management in ARDS patients is very important in addition to treatment of the underlying disease. In particular, mechanical ventilation settings are the highest priority for ARDS patients. Several studies have been conducted regarding ventilation strategies designed for ARDS patients that restrict tidal volume as a lung protective measure to reduce further lung injury and limit airway pressure.

As a result of the systematic review, only six 2013 Cochrane Review^202^‐adopted RCTs^191^, ^203^, ^204^, ^205^, ^206^, ^207^ involving the use of lung protective ventilation methods focusing primarily on low tidal volume in adult ARDS patients were identified. Mortality statistics were reported in all six studies (*n* = 1,305), and while there was a difference in the follow‐up period, this period tended to be shorter in the low tidal volume group (risk ratio: 0.84, 95% confidence interval [CI]: 0.67–1.07). Airway pressure‐associated injury (pneumothorax arising from elevated airway pressure) was also reported in all 6 studies, but no significant decrease was observed (risk ratio: 0.82, 95% CI: 0.48–1.41). Although the results of three RCTs were integrated with respect to the number of ventilator‐free days (VFD), the mean difference significantly increased to 2.52 days (95% CI: 0.53–4.51).

VFDs increase despite the observation of high carbon dioxide levels and respiratory acidosis in conjunction with low tidal volume. Low tidal volume can be used just by adjusting the mechanical ventilator settings, and resources required remain unchanged. Therefore, it is likely that the potential benefits outweigh the potential harms.

### CQ10‐2: How should plateau pressure be set when performing mechanical ventilation in adult ARDS patients?

#### Answer (Recommendation)

We suggest a plateau pressure of ≤30 cmH_2_O when performing mechanical ventilation in adult ARDS patients (2B: excerpted from the ARDSGL).

#### Rationale

Mechanical ventilation‐related lung injury in adult patients with ARDS is likely to occur in conjunction with decreased pulmonary compliance. There has been some concern regarding how VALI not only extends the period of mechanical ventilation but also leads to an increased risk of mortality. Increased tidal volume during mechanical ventilation and elevated airway pressure have been raised as potential causes of VALI, and it is expected that both can be controlled by limiting plateau pressure (airway pressure at the point when airflow temporarily stops at the end of inspiration). However, the results of limiting plateau pressure are not all beneficial; lower plateau pressure may also lead to adverse events such as hypercapnia. Accordingly, the optimum plateau pressure for obtaining treatment benefits without causing VALI is currently uncertain, and validation is necessary and a high priority.

As a result of the systematic review, four RCTs (1,132 patients)^191^, ^204^, ^205^, ^207^ were identified. Prolonged VFD (2.5 days on average, 95% CI: 0.5–4.45) was confirmed by setting plateau pressure to ≤30 cmH_2_O for 5–7 days after the initiation of mechanical ventilation. Although the risk of death (risk ratio: 0.84, 95% CI: 0.62–1.15) and pressure‐related injury (risk ratio: 0.92, 95% CI: 0.65–1.31) exhibited declining trends, these observations were not statistically significant.

VFDs are extended by limiting plateau pressure to ≤30 cmH_2_O. In contrast, hypoxemia, hypercapnia, and respiratory workload are each anticipated to increase, but each has a wide range of tolerance, and the risk of iatrogenic harm is considered low. This is possible only by adjusting the mechanical ventilator settings, and resources required remain unchanged. Therefore, it is likely that the potential benefits outweigh the potential harms.

### CQ 10‐3: How should PEEP values be set when performing mechanical ventilation in adult patients with ARDS?

#### Answer (Recommendation)

We suggest setting PEEP values within a range such that the plateau pressure is ≤30 cmH_2_O and hemodynamics are not impacted when performing mechanical ventilation in adult patients with ARDS (2B: excerpted from the ARDSGL). We suggest the use of higher PEEP values in patients with moderate or severe forms of ARDS (2B: excerpted from the ARDSGL).

#### Rationale

Some evidence suggests that PEEP may be a factor in correcting hypoxemia by promoting the recruitment of collapsed alveoli and preventing progression of VALI, but the optimal PEEP value for this purpose remains unclear.

Seven RCTs were found as a result of the systematic review, and no significant difference was observed between the high PEEP group and the low PEEP group with respect to in‐hospital mortality (risk ratio: 0.93, 95% CI: 0.83–1.04), pressure‐associated injury (risk ratio: 0.97, 95% CI: 0.66–1.42), and VFD (risk ratio: 1.89, 95% CI: −3.58 to 7.36). For the analysis of in‐hospital mortality, studies permitting interventions other than adjustment of PEEP values capable of influencing patient outcomes were excluded from analysis. As a result, three studies, by Brower *et al*.,^208^ Meade *et al*.,^209^ and Mercat *et al*.^210^ were included in the final analysis. As a result of a meta‐analysis involving studies in which the influence of non‐PEEP interventions could not be ignored,^203^, ^207^, ^211^, ^212^ no significant difference in mortality rate was observed in the high PEEP group in comparison with the low PEEP group (risk ratio: 0.87, 95% CI: 0.74–1.02). In addition, when focusing the analysis on moderate or severe ARDS cases (PaO_2_/FiO_2_ ratio ≤200 mmHg), the results of both the sub‐analyses including the studies permitting non‐PEEP interventions as well as the sub‐analyses excluding such interventions indicated that the high PEEP group exhibited a significantly lower mortality rate than the low PEEP group (risk ratio: 0.82, 95% CI: 0.73–0.92, risk ratio: 0.85, 95% CI: 0.75–0.96, respectively).

Setting the PEEP value is possible just by adjusting the mechanical ventilator settings, and there is no extra workload on ICU staff. However, the benefits and risks of this technique remain unclear, and therefore, the benefit‐risk balance is currently uncertain.

### CQ10‐4: How should daily fluid balance be maintained in adult patients with ARDS?

#### Answer (Recommendation)

We suggest fluid restriction when managing adult ARDS patients (2B; excerpted from the ARDSGL).

#### Rationale

Pulmonary edema occurring in ARDS patients is believed to be caused by vascular endothelial damage and vascular hyperpermeability. The larger number of ARDS patients increases the mortality rate, and pulmonary extravascular fluid volume is linked to ARDS severity and frequency of mortality.

However, to date, no RCT has reported an improvement in mortality with fluid management in ARDS patients. Although attempting to optimize body fluid volume is routinely attempted in the treatment of other conditions and is regarded as an important measure, the mechanisms controlling fluid balance in ARDS patients is not well understood. As such, this is a high‐priority issue, and the current recommendation is to manage fluid balance on a daily basis to avoid excessive body fluid volume as much as possible.

As a result of the systematic review, three RCTs comparing adult ARDS patients subjected to some manner of fluid restriction against those who were not were found. Studies permitting the adjustment of the infusion burden on shock patients in addition to ARDS were excluded. Although a large number of cases were included in the Fluids and Catheters Treatment Trial (FACTT 2006 study),^213^ the other two studies RCTs^214^, ^215^ included only a small number of cases. No significant differences in short‐term mortality were observed, and the number of VFDs within a 28‐day period increased significantly (+2.5 days). There were also no differences with respect to renal replacement therapy within a 60‐day period. Regarding the indicators of fluid management, although two RCTs comparing pulmonary extravascular water content with pulmonary artery wedge pressure^216^ and central venous pressure^143^ were found, neither study demonstrated an improvement in mortality rate. The former study results demonstrated a reduction in the mechanical ventilation period, while the latter study failed to demonstrate any particular clinical utility.

Shortening of the VFD can be expected as a result of limiting fluid infusion volume. However, there is a risk of electrolyte abnormalities when diuretics are used. There are currently no clear standard indicators for assessing fluid balance, but many medical institutions use some manner of indicators to evaluate hemodynamics. The addition of new indicators for this purpose is of low necessity as treatment objectives can be achieved with general practice techniques. Thus, the benefits greatly exceed the potential risks.

## CQ11: MANAGEMENT OF ANALGESIA, SEDATION, AND DELIRIUM

### Introduction

Delirium is a mental disorder often encountered in clinical practice and on general wards. It is characterized by a variety of neurological symptoms (such as altered consciousness, attention, and sensory perception) which mostly resolve following an improvement in the patient's physical condition. Delirium commonly progresses rapidly over a period of days, and symptoms may fluctuate. From a psychiatric point of view, delirium is a type of consciousness‐related disorder accompanied by varying degrees of altered perception and mild dulling of the awareness. Patients presenting with delirium exhibit various symptoms, and the disorder is classified into three subtypes depending on the symptoms observed: hyperactive, hypoactive, and mixed. Among these subtypes, hyperactive delirium is most frequently addressed by therapeutic intervention as it is easily recognized by general medical staff due to the severity of its presentation and the hindrance it poses to treatment. In contrast, hypoactive delirium rarely presents as dangerous behavior and does not require extraordinary effort from the nursing staff. At first glance, hypoactive delirium can appear to be a state of “sustained sedation,” and to date has been actively diagnosed infrequently. However, delirium presents in an extremely hypoactive form in many cases, particularly those involving the management of critically‐ill patients such as intensive care unit (ICU) patients, and some studies have reported that many delirious ICU patients are neglected and left unattended by medical staff.^217^ Another report states that delirium occurring in critically‐ill patients requiring mechanical ventilation is an independent risk factor for patient outcome, and can have a significant adverse impact on prognosis.^218^ ICU delirium, a type of acute cerebral disorder that affects the central nervous system is one of several organ disorders that affect critically‐ill patients. Moreover, the central nervous system, after the respiratory and cardiovascular systems, is the most frequent target of physicians’ attention regarding organ systems that are prone to damage in critically‐ill patients.^219^ Therefore, regular monitoring of the central nervous system is recommended and should be accorded the same degree of importance as monitoring the respiratory state and hemodynamics over time using metrics such as SpO_2_, blood gas content, blood pressure, and electrocardiography.

Although the causes and underlying mechanisms of various mental disorders and risk factors affecting ICU patients as described above remain unknown, post‐traumatic stress disorder (PTSD), the subject with the largest number of case reports, hallucinations, and delusions experienced by ICU patients are drawing research attention. Also, prolonged periods of delirium giving rise to hallucinations and delusions during hospitalization has also been recognized as a key independent risk factor^220^ associated with long‐term cognitive dysfunction after discharge. Currently, the onset of delirium is considered a factor that can significantly worsen the long‐term prognosis after discharge of critically‐ill patients, and it is believed that these are two iatrogenic risks arising from deficient management by medical staff.^221^, ^222^ Accordingly, the general importance of taking suitable measures to address delirium must be emphasized.

Although haloperidol and various atypical antipsychotics have been used in the management of delirium in the past, there have been no reports to date proving their efficacy as treatments for ICU delirium, including postoperative delirium,^223^, ^224^, ^225^, ^226^, ^227^ and there are only a few studies that can assert to the efficacy of quetiapine.^228^ Currently, while the use of antipsychotics may slightly reduce the frequency of delirium onset, the consensus is that their routine use is not recommended, as it does not ultimately lead to improved patient prognosis while exposing them to various side effects including extrapyramidal symptoms, torsade de pointes, and ventricular arrhythmias.^229^ However, dexmedetomidine, which has sedative effects and acts similarly to the induction of natural sleep, has long been expected to become a treatment for delirium and various clinical studies have been conducted to date, but, due to methodological inadequacies and other obstacles, a solid conclusion is yet to be reached. Thus, the reality is that there are currently no drugs proven to be effective as treatments for delirium. However, as delirium manifests as a disorder of the central nervous system and is an independent risk factor that affects patient prognosis, some manner of response is required, and if insufficient results are obtained regarding pharmacological approaches, non‐pharmacological approaches will become critical.

The basic principle in treating delirium is the identification and elimination of causative factors, and the first step in non‐pharmacological approaches is to lower patients’ stress by adjusting their environment; physicians must determine how patients lived their lives before being hospitalized. Among these non‐pharmacological approaches, the promotion of nighttime sleep and early rising, in particular, have attracted attention in recent years. Unfortunately, although no study regarding sleep management with an overall high level of evidence has been conducted to date, improving sleep quality is believed to offer various health benefits, and many authorities recommend this approach.^230^ Also, the promotion of early rising has accumulated a strong body of supporting evidence with respect to critically‐ill patients.^231^, ^232^ In addition, as the only non‐pharmacological approach shown to be effective in the treatment of ICU delirium is early‐stage rehabilitation, its implementation is strongly recommended.

Issues concerning analgesia/sedation have already been highlighted in many reports as a way of addressing delirium in critically‐ill patients and the onset of neurological disorders after intensive care. As it is clear that excessively sustained sedation unnecessarily prolongs mechanical ventilation periods due to factors such as the onset of ventilator‐associated pneumonia and unsuccessful weaning trials,^233^, ^234^, ^235^, ^236^ the mainstream approach is shifting from “hypnosis‐focused sedation” to a “minimum sedation.” Preventing hallucinations and delusions is also important, and to do this, attending physicians should “avoid unnecessary sedation.” The safety of early‐stage rehabilitation in critically‐ill patients has also been pointed out,^237^ but reports made around the same time have highlighted how rehabilitation measures are infrequently implemented.^238^, ^239^ The issue of “excessive sedation” has been raised as a possible reason. Implementing rehabilitative measures will naturally become difficult if a patient's sedation is excessively deep such that delirium cannot be assessed. Establishing a basic policy of directing physicians “to avoid unnecessary sedation” is necessary from the perspectives of performing routine delirium assessments and early‐stage rehabilitation.

There is a need for careful planning when managing critically‐ill patients, such as those under mechanical ventilation, in line with the policy of “administer the minimum sedation necessary while avoiding unnecessary sedation,” with a primary focus on “sufficient pain relief.” The sedatives currently indicated for use in mechanically ventilated patients in Japan (midazolam, propofol, and dexmedetomidine) alone do not have clinically satisfactory analgesic effects, and increasing patients’ dosages and deepening sedation in response to complaints of pain is counterproductive. In contrast, if pain can be sufficiently managed with opioids or similar drugs, it is possible to manage even critically‐ill mechanically ventilated patients with “no sedation”.^240^ When selecting an analgesic, opioids, whose effects are virtually predictable, should be the first choice, followed by fentanyl or morphine, although the former is the mainstay in Japan. However, the use of narcotic‐antagonist analgesics, which are widely used in Japan, is desirable only after fully understanding their analgesic mechanism, such as avoiding concomitant use of opioids. Combination therapies such as acetaminophen + nonsteroidal anti‐inflammatory drugs (NSAIDs) can also be effective to reduce opioid usage.

The current basic principle in the management of critically‐ill patients including sepsis patients is “minimum necessary sedation based on adequate pain management protocols and frequent delirium assessment; facilitate rehabilitation as quickly as possible,” and this concept is already encapsulated as the ABCDE bundle.^221^, ^222^ Recently, the Japanese Society of Intensive Care Medicine published the Japanese guidelines for the management of Pain, Agitation, and Delirium in intensive care unit (J‐PAD guidelines)^241^ based on the Clinical practice guidelines for the management of pain, agitation, and delirium in adult patients in the intensive care unit (PAD guidelines)^242^ in consideration of the medical circumstances of Japan and new evidence after the PAD guidelines. Therefore, the guideline committee extracted recommendations from the J‐PAD guidelines. Thus, the recommendations in this area included some contents of the J‐PAD and PAD guidelines.

### CQ11‐1: What clinical outcomes can be expected with respect to delirium in adult ICU patients?

#### Answer

1. Delirium is associated with worsens ICU patients’ prognoses (A: excerpted from the PAD guidelines).
2. Delirium is associated with longer ICU stay time and overall hospitalization time (A:excerpted from the PAD guidelines).
3. Delirium is linked to impaired cognitive function following discharge from the ICU (B: excerpted from the PAD guidelines).

#### Rationale

Delirium occurring in critically‐ill patients is an acute cerebral disorder and a form of multiple organ dysfunction involving the central nervous system. Similar to other types of vital organ dysfunction, it is believed to worsen both short‐term and long‐term patient prognosis. The PAD guidelines^242^ also state, based on numerous high‐quality observational studies, that the onset of ICU delirium increases ICU stay time as well as the overall hospitalization time, and can be a cause of impaired cognitive function and neurological disorders in the long‐term. The PAD guidelines further state that the onset of delirium in critically‐ill patients is also an independent risk factor for poor prognosis, irrespective of the underlying disease. Due to these observations, it is important for physicians attending to sepsis patients in the ICU to correctly recognize the effects of delirium, as such patients frequently have sepsis as an underlying disease.

### CQ11‐2: Should a non‐pharmacological delirium protocol be followed when treating adult ICU patients to reduce the incidence and duration of delirium episodes?

#### Answer (Recommendation)

1. We recommend discharge from the ICU as early as possible to reduce the onset and duration of delirium (1B: excerpted from the PAD guidelines).
2. We suggest the use of musical interventions when possible to decrease the amount of sedatives required and to reduce patient anxiety (2C).

#### Rationale

The basic principle in treating delirium is the identification and elimination of causative factors, and the first step in implementing a non‐pharmacological delirium protocol is to alleviate patients’ stress through appropriate adjustments to their environment. The promotion of early rising and sleeping during the night has attracted particular attention in recent years.

Results of early rising in interventional studies targeting critically‐ill adult subjects^231^, ^232^ indicated reduced delirium incidence, a reduction in excessive sedation, and significant shortening of ICU stay time as well as overall hospital admission time at moderate evidence levels. PAD guidelines^242^ have also accumulated a body of supporting evidence with respect to critically‐ill patients. Currently, as the only effective non‐pharmacological approach in the treatment of ICU delirium is early‐stage rehabilitation, its implementation is strongly recommended. Regarding sleep promotion, unfortunately, at the moment there is no research with a high level of evidence, but it is easy to appreciate that improvement of sleep quality is advantageous to patients, and many authorities recommend this.

Although one randomized controlled trial (RCT) examining musical interventions for patients under mechanical ventilation has been conducted,^243^ as there are currently no musical therapists currently practicing at medical institutions in Japan, introducing this type of intervention at this time is believed to be difficult. Medical interventions using music require assessing together with related medical staff which types of music and sound quality are likely to produce the desired clinical effects, methods for reducing noise and other interferences, the selection of suitable speaker devices, among various other potential factors. However, it is unlikely that interventions using music pose any harm to patients, and even if the current body of supporting evidence level is low, this approach may be considered in addition to daily therapy regimens. Although few studies regarding musical interventions are limited to sepsis patients, each of these reports is considered applicable to sepsis patients as well.

### CQ11‐3: Should a pharmacological delirium prevention protocol be followed when treating adult ICU patients to reduce the incidence and duration of delirium episodes?

#### Answer (Recommendation)

The use of a pharmacological delirium prevention protocol to reduce the onset and duration of delirium in adult ICU patients is not necessarily required (insufficient data; C: excerpted from the PAD guidelines).

#### Rationale

While haloperidol and various atypical antipsychotic drugs have conventionally been used as drug therapies for delirium, very few reports have been published to date supporting the efficacy of these therapies with respect to ICU delirium (including postoperative delirium). The PAD guidelines^242^ state that the use of these drugs cannot be recommended firmly due to a lack of supporting data. In addition, the results of a meta‐analysis^229^ verifying the effects of non‐pharmacologic delirium prevention protocols in critically‐ill adult patients also indicate that the prophylactic administration of antipsychotics to surgical ICU patients and the prophylactic administration of dexmedetomidine to mechanically ventilated patients may decrease the incidence of delirium. However, others argue that these drugs are generally used to treat delirium and cannot be said to have a significant impact on clinical outcomes, including mortality rate.

Currently, while there is a possibility that antipsychotics may cause a slight decrease in the frequency of delirium, this may not necessarily lead to improvement in a patient's final prognosis, and in consideration of the various side effects of antipsychotics (e.g., onset of extrapyramidal symptoms, torsade de pointes, ventricular arrhythmias, etc.), the use of these drugs cannot be recommended as a routine approach. As the recognition of these observations is not yet mainstream, the importance of this particular clinical question is considered to be high.

### CQ11‐4: Should a “discontinue daily sedation” or an “aim for a mild depth of sedation” protocol be followed when treating adult patients under mechanical ventilation?

#### Answer (Recommendation)

We recommend the routine application of a “discontinue daily sedation” protocol or an “aim for a mild depth of sedation” protocol when treating adult patients under mechanical ventilation (1B: excerpted from the PAD guidelines).

#### Rationale

A moderate level of evidence indicates that a protocol calling for “discontinuing daily sedation” can improve prognosis more than continuous sedation in critically‐ill mechanically ventilated adult patients. A moderate level of evidence similarly indicates that a protocol calling for “aiming for a mild depth of sedation” can improve patient prognosis more than maintaining a deep level of sedation. For these reasons, the PAD guidelines^242^ also recommend that either of these protocols is used when determining a type and depth of sedation. However, there is currently insufficient data to support protocols calling for “discontinuing daily sedation” or “aiming for a mild depth of sedation,” and even the PAD guidelines have not yet been able to offer a consensus.

Based on the above, the current consensus is that attending physicians may select sedation protocols calling for either “discontinuing daily sedation” or “aiming for a mild depth of sedation.” In addition, although there were few studies limited to sepsis patients and most of the studies forming the basis for this consensus targeted general critically‐ill adult patients, each of these studies is considered also to apply to sepsis patients.

It is already clear that excessive maintenance of a sedated state unnecessarily prolongs the period of mechanical ventilation leading to the occurrence of ventilator‐associated pneumonia and unsuccessful weaning trials. Critically‐ill patients must be kept as alert as possible to conduct routine delirium monitoring, which has high clinical importance.

### CQ11‐5: Should “analgesia‐first sedation” or “hypnosis‐focused sedation” be used when treating adult patients under mechanical ventilation?

#### Answer (Recommendation)

We suggest the use of “analgesia‐first sedation” when treating adult patients under mechanical ventilation (2B: excerpted from the PAD guidelines).

#### Rationale

Analgesia‐first sedation has been demonstrated to result in greater improvement in patient prognosis in comparison to hypnosis‐focused sedation in critically‐ill adult patients under mechanical ventilation, and the PAD guidelines^242^ also recommend this approach with the support of a moderate level of evidence. In addition, although critically‐ill patients must be kept as alert as possible in order to routinely assess for delirium, maintaining the sedation level of these patients, who are under a great deal of stress, as low as possible must also provide “sufficient pain relief,” and has high clinical importance.

Many of the studies underpinning these observations targeted general critically‐ill adult patients, and although there are few studies limited to sepsis patients, each of these reports is considered to apply to sepsis patients as well.

## CQ12: ACUTE KIDNEY INJURY/BLOOD APHERESIS

### Introduction

The Risk, Injury, Failure, Loss, End‐Stage Kidney Disease (RIFLE) classification proposed by the Acute Dialysis Quality Initiative (ADQI) in 2004^244^ was the first specific, internationally accepted, universally understandable, and widely accepted definition of acute renal failure. The clinical utility of the RIFLE criteria with respect to metrics such as prognostic prediction has already been demonstrated via meta‐analysis.^245^ A subsequent report stated that slight increases in serum creatinine values could have a substantial impact on patient prognosis.^246^ In 2007, the Acute Kidney Injury Network (AKIN) defined acute kidney injury (AKI) as a condition characterized by a very mild increase in serum creatinine (sCre) as well as (i) ΔsCre ≥0.3 mg/dL (within 48 h), (ii) a 150% increase from the baseline sCre value (within 7 days), and (iii) hourly urine volume of ≤0.5 mL/kg. The AKIN criteria, a modified version of the RIFLE criteria, was also proposed at the same time.^247^ Furthermore, the Kidney Disease: Improving Global Outcomes (KDIGO) group presented the AKI Clinical Practice Guidelines in 2012, which summarized the then‐current body of evidence and also proposed the KDIGO criteria, which integrated the RIFLE and AKIN criteria.^248^ Today, the KDIGO criteria are widely used in the assessment of acute‐stage renal disorders and have also been adopted as an entry standard in various clinical studies.

Some epidemiological studies using the internationally accepted AKI definition in this manner have since been reported. Among the various etiologies of AKI, AKI arising from sepsis (sepsis‐induced AKI) most frequently occurs in patients requiring intensive care and is said to comprise 30%–70% of all cases of AKI.^249^ Other reports have also stated that sepsis‐induced AKI occurs in ~10%–20% of all intensive care unit (ICU) patients.^250^ Furthermore, because sepsis‐induced AKI often results in serious injury to vital organs due to the persistent overproduction of inflammatory mediators, this type of AKI increases in severity more rapidly than AKIs of other etiologies and various studies have highlighted its association with a high rate of mortality. Conversely, recovery of renal function can be easily achieved if a patient's general condition improves.^249^ Based on these observations, diagnosing AKI at an early stage is particularly critical in sepsis cases in order to prevent disease progression. Thus, the first clinical question (CQ) in this chapter addresses the diagnosis of sepsis‐induced AKI.

Acute blood apheresis techniques such as hemodialysis and hemofiltration are initiated to avoid life‐threatening events when AKI progresses and marked decline in renal function is observed. Blood apheresis is a supplementary therapy to the function of the kidney, rather than a curative treatment for kidney damage. There is believed to be no room for debate concerning the utility of emergency initiation of blood apheresis in cases of pathological conditions such as life‐threatening hyperkalemia, advanced acidosis, and overflow. However, to maintain homeostatic conditions such as the electrolyte and acid‐base equilibria and the adjustment of body fluid volume, earlier initiation of blood apheresis, adjustments in treatment approaches (amount of treatment given, etc.), and stronger measures to improve abnormalities are likely correlated with improved survival rates and recovery of kidney function, and various studies have been conducted from this standpoint. Several CQs on this topic as well as the AKI clinical practice guidelines behind the creation of KDIGO are presented.

Sepsis‐induced AKI often develops as a type of organ disorder in which the dysfunction of several vital organs is induced sequentially as a result of prolonged secretion of large quantities of inflammatory mediators as described above. As such, the goal of acute blood apheresis is not only to supplement normal renal functions such as maintaining the balance of electrolytes, the acid‐base equilibrium, and appropriate body fluid volume but has also come to include the treatment and prevention of organ disorders through the removal and control of these inflammatory mediators. Although there have been many studies examining whether earlier initiation of blood apheresis or adjustments in treatment approaches can be expected to lead to further improvements in survival rates and recovery of renal function in sepsis‐induced AKI, it must be emphasized that there are numerous studies examining renal supplementation as well as other treatment objectives, and careful interpretation is necessary when assembling bodies of evidence.

With respect to the performance of acute blood apheresis, it cannot be overstated that there were never any standardized aspects (e.g., modality selection, initiation/discontinuation criteria, treatment procedure, etc.) at any point of treatment provision. Because of this, not only treatment modality and initiation/discontinuation criteria, but also the volume of blood filtered (and if dialysis and filtration are performed simultaneously, the proportion of blood filtered as well), the route used to administer fluid supplementation, the type of dialysis/apheresis apparatus, the frequency of exchange, the type(s) of anticoagulants administered, as well as many other factors pertaining to treatment methodology must be clarified to determine the optimal treatment approach for a given pathology in actual practice. While selecting CQ topics in this area, it was believed that of the various aspects of the methodology of treatment mentioned previously, CQ12‐2 on whether blood apheresis should be performed early, CQ12‐3 on whether blood apheresis should be performed continuously or intermittently, and CQ12‐4 on whether the volume of blood to be filtered should be increased were suitable for presentation in consideration of their respective importance and quality of the accompanying evidence.

Polymyxin B‐immobilized fiber column direct hemoperfusion (PMX‐DHP) is performed as a specialized acute blood apheresis technique intended for the control and removal of inflammatory mediators. This technique was developed in Japan in 1994 as an endotoxin adsorption column, is covered by the Japan National Health Insurance (NHI), and is widely performed as a supportive therapy in patients with septic shock. As such, CQ12‐5 discusses this technique.

Next, no specific and effective treatment for AKI has been established to date. The utility of some drugs (furosemide, dopamine, and carperitide) has been studied, and these studies are discussed in CQs 12‐6 to 12‐8. Furosemide is expected to be useful in the treatment of AKI, as it prevents renal tubular obstruction caused by detached cells through its diuretic action arising from suppression of sodium reabsorption, raising oxygen concentration and increasing blood flow to the renal medulla. Dopamine is believed to have renoprotective effects due to its ability to cause renal vasodilation and to suppress sodium reabsorption, especially at low doses (1–3 μg/kg/min). Carperitide (atrial natriuretic peptide, ANP) has demonstrated vasodilative action, suppressive action on sodium reabsorption, ability to increase glomerular filtration rate by means of afferent arteriolar dilation and efferent arteriolar contraction, and is also believed to have renoprotective effects arising from its diuretic and glomerular filtration rate enhancing properties. Many reports have stated that these drugs do not improve outcomes as measured by survival rates and rates of initiation of dialysis. Meanwhile, furosemide has been associated with side effects such as tinnitus and hearing loss, dopamine may increase the risk of developing arrhythmias, and carperitide has been associated with side effects such as hypotension. Accordingly, the corresponding CQs for these drugs will examine their clinical utility.

### CQ 12‐1: Are the KDIGO clinical practice guidelines useful for diagnosing sepsis‐induced AKI?

#### Answer (Opinion)

We recommend applying of the KDIGO clinical practice guidelines for diagnosing and determining the severity of sepsis‐induced AKI (expert consensus/quality of evidence “D”; rate of agreement: 100%).

#### Rationale

Seven observational studies^251^, ^252^, ^253^, ^254^, ^255^, ^256^, ^257^ examining patient mortality rate as a clinical outcome were extracted during an assessment comparing the KDIGO guidelines and the AKIN and RIFLE criteria, and as a result of comparing AKI diagnoses based on the KDIGO guidelines with diagnoses based on the RIFLE or AKIN criteria, diagnoses based on the AKIN criteria were found to have higher accuracy or were reflective of similar in‐hospital mortality rates. Although only the study by Peng *et al*.^257^ focused specifically on sepsis cases, the KDIGO guidelines are currently still considered to be useful in predicting prognosis in patients with sepsis‐induced AKI. However, few studies regarding renal prognosis have been conducted to date, and currently little is known about this aspect. All published studies found were observational studies, and there have been no studies assessing diagnostic criteria as an interventional approach. No additional burden is placed on patients or medical personnel during diagnostic assessment. Additional medical costs are required for tests procedures such as measurement of sCre, urine volume measurement over time, etc. Virtually all ICUs are capable of performing such measurements, and it is likely that the potential benefits outweigh the potential harms.

### CQ 12‐2: Should blood apheresis be initiated at an early stage in patients with sepsis‐induced AKI?

#### Answer (Recommendation)

We suggest against the initiation of blood apheresis at an early stage in sepsis‐induced AKI except when emergency apheresis is necessary, such as those involving advanced metabolic acidosis, hyperkalemia, or renal overflow (2C; rate of agreement: 100%).

#### Rationale

Blood apheresis in sepsis‐induced AKI has come to be performed not only to supplement renal function but also to prevent or treat organ disorders through the removal and control of inflammatory mediators. Initiation of blood apheresis at an early stage is believed to improve the ease with which inflammatory mediators can be removed and controlled, and many relevant randomized controlled trials (RCTs) have been conducted in recent years. Although most of these RCTs are multiple objective studies examining both renal supplementation and inflammatory mediator removal, attempting to assess these purposes separately is not practical. A new meta‐analysis was conducted after the results of two large‐scale RCTs were reported in May 2016.

Three RCTs^258^, ^259^, ^260^ were used to perform the new meta‐analysis. In the results, the impact of early‐stage initiation of blood apheresis on 28‐day or 30‐day mortality was a risk ratio of 0.83 (95% confidence interval [CI]: 0.64–1.09), and a risk ratio of 0.51 for the rate of dialysis dependence on day 60 (95% CI: 0.25–1.06). No statistically significant differences and no benefits of early‐stage initiation were observed. However, although there was no significant difference in the rate of dialysis dependence, early‐stage initiation is expected to have some benefits regarding clinical utility. Two multicenter RCTs^261^, ^262^ were ongoing as of June 2016, and the consensus on this subject may change based on their results.

Although decreased mortality rate is an expected benefit of this intervention, no differences between the intervention group and the control group were observed with respect to ICU stay time, 28‐day mortality rate, or rate of transition to chronic dialysis. In general, hemorrhagic complications and other adverse events are recognized as potential harms, but no differences in the incidence of these events were observed between the study groups in these two RCTs. The burden on medical staff will also increase as a result of this intervention. The potential harms will outweigh the potential benefits, and associated medical costs and staff burden will also increase. Moreover, there are serious concerns over the feasibility of performing this intervention at facilities without adequate human resources or staff proficient in its implementation.

### CQ 12‐3: Should blood apheresis be performed continuously or intermittently in patients with sepsis‐induced AKI?

#### Answer (Recommendation and opinion)

We suggest that attending physicians select either continuous or intermittent blood apheresis when treating sepsis‐induced AKI patients exhibiting stable hemodynamics (2B; rate of agreement: 94.7%).

We suggest selecting continuous apheresis in patients exhibiting unstable hemodynamics (expert consensus/no evidence; rate of agreement: 84.2%).

#### Rationale

When deciding whether to perform continuous or intermittent blood apheresis, it is important to consider various factors such as whether the medical staff can cope with the additional workload and whether suitable equipment are available. If either option is feasible, the discretion of the attending clinician during selection is considered to be of high importance.

Only one new RCT has been conducted since the existing systematic review,^263^ which also yielded the same results as the systematic review, and so the assessment was made using the existing systematic review. A total of 15 studies were examined; “in‐hospital mortality” was assessed based on seven RCTs^264^, ^265^, ^266^, ^267^, ^268^, ^269^, ^270^ and yielded a risk ratio of 1.01 (95% CI: 0.92–1.12); “transition to chronic dialysis” was examined based on three RCTs^264^, ^266^, ^269^ and yielded a risk ratio of 1.01 (95% CI: 0.92–1.07); no difference was observed between apheresis performed continuously and intermittently. Three RCTs^269^, ^270^, ^271^ were also examined with respect to reduction in blood pressure, and no differences in the risk ratio of 0.92 (95% CI: 0.72–1.16) were observed in the studies. However, the two RCTs^266^, ^268^ extracted from the systematic review followed protocols that excluded cases involving patients with unstable hemodynamics. Continuous blood apheresis is already recognized as a standard treatment in such cases, and no RCT conducted to date has compared continuous and intermittent apheresis.

Continuous and intermittent blood apheresis are considered to be equivalent regarding potential benefits relating to in‐hospital mortality rate and rate of transition to chronic dialysis. Although there is currently a lack of evidence regarding potential harms, continuous blood apheresis is associated with an increased risk of bleeding, greater medical costs arising from a longer implementation period, and increased workload on medical technicians and nurses.

Based on the above, the expert consensus reached is that continuous apheresis is preferable when patients present with unstable hemodynamics. However, because continuously and intermittently performed variants of blood apheresis have different characteristics, in cases where minimizing anticoagulant use is necessary, attending intensive care physicians or renal specialists must carefully consider these characteristics when determining whether to implement measures such as performing short‐term apheresis without administering an anticoagulant.

### CQ12‐4: Is increasing the volume of blood filtered via blood apheresis beneficial in sepsis‐induced AKI cases?

#### Answer (Recommendation and opinion)

We recommend against increasing the volume of blood filtered beyond the international standard volume (20–25 mL/kg/h) (1B; rate of agreement: 89.4%). It should also be noted that the evidence supporting the blood filtration volume covered by the NHI system (10–15 mL/kg/h) is weak (expert consensus/no evidence; rate of agreement: 73.7%).

#### Rationale

RCTs are actively being conducted elsewhere to assess whether increasing blood filtration volume (filtrate volume + dialysis volume) can improve patient outcomes in sepsis‐induced AKI. However, the NHI system defines an upper limit for filtration volume that is coverable under the system. As such, this CQ will reassess the current body of evidence.

No new RCTs were completed since the existing systematic review,^272^ and the assessment for this CQ was conducted using this systematic review. Eight RCTs comparing an intervention group (high‐volume: 40 mL/kg/h) and a control group (international standard quantity: 25 mL/kg/h) were assessed with respect to “28‐day mortality rate.” In addition, “recovery from kidney failure” was used to evaluate “transition to chronic dialysis.” The risk ratio for “28‐day mortality rate” was 0.89 (95% CI: 0.76–1.04) and the risk ratio for “recovery from kidney failure” was 1.12 (95% CI: 0.95–1.31). Treatment effects were evaluated in the same manner even if the blood filtration volume was increased. There is no evidence from Japan regarding a comparison of the NHI‐defined filtration volume and the international standard volume, and so evaluation was not possible.

No differences between the intervention and control groups were observed with respect to 28‐day mortality rate and the rate of transition to chronic dialysis. Based on these findings, the effects of blood apheresis remain unchanged even after increasing the blood filtration volume. Although there is no supporting evidence, when selecting a fluid replacement solution for blood filtration, which are widely used as dialysis fluids or fluid replenishers in Japan, electrolyte imbalances such as hypokalemia and hypophosphatemia can easily occur as a result of increasing the apheresis volume. The labor burden placed on medical technicians and nurses who replace fluid pouches will increase as a result of this intervention. In consideration of the above, the potential harms likely outweigh the potential benefits.

### CQ12‐5: Is performing PMX‐DHP recommended when treating patients with septic shock?

#### Answer (Recommendation)

We suggest against performing PMX‐DHP as a standard treatment for patients with septic shock (2C; rate of agreement: 84.2%).

#### Rationale

PMX‐DHP is a specialized variant of acute blood apheresis used to remove and control inflammatory mediators. This technique was developed in Japan in 1994 as an endotoxin adsorption column, is covered by the NHI, and is widely performed as a supportive therapy in patients with septic shock.

Three RCTs (Vincent *et al*.^273^; Cruz *et al*.^274^; and Payen *et al*.^275^) were extracted as a result of the systematic review conducted for this CQ. However, the participants in each of these studies presented with septic shock arising from intraperitoneal infection requiring emergency abdominal surgery. Each of these RCTs reported increased mortality, and two reported increased mean blood pressure. In contrast, none of these RCTs reported results related to shock recovery rate. The odds ratio with regard to the impact of PMX‐DHP on mortality rate was 1.1 (95% CI: 0.68–1.79), and no improvements in survival rate were observed. The studies by Vincent *et al*.^273^ and Cruz *et al*.^274^ examined increases in mean blood pressure. The mean difference observed was 4.59 (95% CI: −1.71 to 10.90), and no significant improvements in blood pressure were observed. No differences between the intervention and control groups were observed with respect to decreases in mortality rate or increases in mean blood pressure. Moreover, these RCTs did not assess shock recovery rate, and also did not recognize thrombocytopenia as an adverse event. Meanwhile, Payen *et al*.^275^ reported a significant decrease in the incidence of thrombocytopenia occurring on day 3 of treatment in the PMX‐DHP group, but did not quantify the reductions in platelet count. Based on the above, the potential risks associated with PMX‐DHP are likely to outweigh the potential benefits.

All RCTs extracted for this CQ targeted subjects presenting with septic shock arising from intraperitoneal infection, and no RCTs targeting other patients with septic shock could be found. For this reason, cases other than those involving intraperitoneal infections cannot be examined at this time due to a dearth of evidence. One large‐scale RCT, the EUPHRATES (Evaluating the Use of Polymyxin B Hemoperfusion in a Randomized controlled trial of Adults Treated for Endotoxemia and Septic shock) study^276^ is currently in progress and is scheduled to be completed in 2016. The results of the EUPHRATES study are expected to be noteworthy as it targets participants presenting with septic shock arising from diseases other than intraperitoneal infection such as pneumonia, and also focuses on severe cases.

### CQ12‐6: Should furosemide be administered to prevent or treat sepsis‐induced AKI?

#### Answer (Recommendation)

We suggest against administering furosemide to prevent or treat sepsis‐induced AKI (2B; rate of agreement: 94.7%).

#### Rationale

Although furosemide inhibits sodium reabsorption and produces a diuretic effect, this drug is expected to prevent or promote improvement in AKI by maintaining steady urine volume, and numerous clinical studies have been conducted to assess this potential.

Two meta‐analyses^277^, ^278^ and eleven RCTs were extracted. The results of both meta‐analyses indicated no effect on in‐hospital mortality rate and the need for blood apheresis as a result of furosemide administration, and no new RCTs have been completed to date. Although decreased mortality and need for dialysis were the expected benefits of this intervention, no significant differences between the intervention groups that received furosemide as a preventive or treatment and the control groups were observed with regard to in‐hospital mortality and necessity of dialysis. Administration of high‐dose furosemide (1–3.4 g/day) was associated with temporary hearing loss and tinnitus^278^ (risk ratio 3.97, 95% CI: 1.00–15.78). Based on the above findings, the potential harms likely outweigh the potential benefits.

According to the meta‐analysis by Ho *et al*.,^277^ furosemide administration demonstrated no significant effect on in‐hospital mortality or the need for blood apheresis. Likewise, furosemide is not recognized as effective for the prevention of AKI or for promoting recovery of impaired renal function. Neither of the two RCTs that were limited to AKI patients undergoing blood apheresis showed a significant decrease in the blood apheresis period (days) or earlier recovery from renal dysfunction in the furosemide group. In addition, the results of another meta‐analysis^278^ indicated that high‐dose furosemide, which is often given to treat AKI, resulted in a significantly increased incidence of symptoms such as tinnitus and hearing loss in comparison with the control group. In contrast, it has also been reported in actual clinical practice that the administration of furosemide improves electrolyte imbalances such as excessive body fluid volume and hyperkalemia. However, no RCTs limited to AKI patients presenting with such clinical signs have been reported to date. It should be noted that the above recommendation does not rule out the use of furosemide to manage excess body fluid volume.

### CQ12‐7: Should dopamine be administered to prevent or treat sepsis‐induced AKI?

#### Answer (Recommendation)

We recommend against adinistering dopamine to prevent or treat sepsis‐induced AKI (1A; rate of agreement: 100%).

#### Rationale

Although dopamine is expected to cause renal vasodilation and natriuresis at low doses (1–3 μg/kg/min) resulting in a renoprotective effect, its clinical utility has not yet been verified. In contrast, there are also concerns that dopamine administration can lead to the incidence of adverse effects such as tachycardia, myocardial ischemia, and reduced intestinal blood flow. Complications such as arrhythmia and cardiac/limb/skin ischemia have occurred clinically, but no significant increase in these events was observed in the meta‐analysis targeting AKI cases. However, it has been reported that dopamine administration in the treatment of sepsis significantly increases the frequency of arrhythmias.^279^ Based on the above observations, clarifying the clinical utility of low‐dose dopamine in patients with sepsis‐induced AKI is considered to be of importance to attending physicians.

Two meta‐analyses were extracted,^279^, ^280^ and it has been shown that administering dopamine does not lead to improvement in terms of either prolonged survival time after receiving low‐dose dopamine or decreased rate of dialysis initiation. No new RCTs involving patients with sepsis have been completed to date.

Although reduced mortality and need for dialysis were expected benefits of this intervention, no significant differences between the intervention and control groups in terms of in‐hospital mortality rate and the need for dialysis were observed.

The risk ratio for adverse effects relating to arrhythmia and ischemic findings was 1.13 (95% CI: 0.90–1.41) and was not statistically significant. Based on the above, the potential harms likely outweigh the potential benefits.

### CQ12‐8: Should ANP be administered to prevent or treat sepsis‐induced AKI?

#### Answer (Recommendation)

We suggest against administering ANP to prevent or treat sepsis‐induced AKI (2B; rate of agreement: 94.7%).

#### Rationale

ANP (carperitide, a synthetic analogue) is a cardiac hormone discovered in Japan together with brain natriuretic peptide (BNP) and C‐type natriuretic peptide (CNP), and acts as a vasodilator, suppresses sodium reabsorption, and increases glomerular filtration rate via dilation of the afferent renal arterioles and contraction of the efferent renal arterioles. In the prevention and treatment of AKI, ANP is expected to exhibit a renoprotective effect resulting from its diuretic effect and ability to increase glomerular filtration rate, and many clinical studies have been conducted to verify this effect. However, some have reported that the administration of high doses of ANP can increase the frequency of adverse events such as hypotension and arrhythmia. Based on the above, clarifying the clinical utility of ANP in patients with sepsis‐induced AKI is considered to be of importance to attending physicians.

Two meta‐analyses were extracted, and neither demonstrated any reduction in the frequency of blood apheresis. Subsequently, no new RCTs on this subject have been completed to date. The incidence of complication by hypotension significantly increased (risk ratio: 1.69; 95% CI: 1.29‐ 2.22), but lower doses did not yield a significant association with hypotension (risk ratio: 1.25; 95% CI: 0.87–1.81). In addition, the AKI preventive effect obtained with low doses has been verified based primarily on cases of AKI occurring after heart surgery, and the results of another meta‐analysis^281^ also suggest the utility of ANP. However, the level of assessment with respect to sepsis cases remains insufficient.

Although reduced mortality and need for dialysis were expected benefits of this intervention, no significant differences between the intervention and control groups were observed regarding in‐hospital mortality rate and the need for dialysis.

Overall, associated risks included the significantly greater likelihood of complication by hypotension, and lower doses did not yield a significant association with hypotension. Based on the above, the potential harms likely outweigh the potential benefits.

## CQ13: NUTRITION

### Introduction

Catabolism is accelerated during serious conditions such as trauma, burns, or sepsis.^282^, ^283^, ^284^ Malnutrition progresses due to catabolic processes, increasing susceptibility to infection and resulting in a deterioration of physiological function. Then, it leads to an increase in the frequency of infections, duration of ventilation, the length of hospital stay, and mortality rate.^285^ Appropriate nutritional intervention has been demonstrated to control these vital reactions and improve prognosis.^286^

This guideline presents five Clinical Questions (CQs) focusing on basic aspects of administering nutritional supplementation to sepsis patients. Recommendations offered by CQ13‐1, 3, 4, and 5 were formulated based on either the same procedures for conducting systematic reviews and meta‐analyses applied in the preparation of the Japanese Guidelines for Nutrition Support Therapy in the Adult and Pediatric Critically Ill Patients (hereinafter, the JSICM guidelines),^287^ or were created based on the results of a meta‐analysis examining randomized controlled trials (RCTs) pertaining to the subject of the CQ. While CQ13‐2 was prepared based on the existing international guidelines reflected in the JSICM guidelines, a literature review, systematic review, and meta‐analysis were each conducted again in the preparation of this CQ. Little evidence limited to sepsis patients could be found for any CQ, and recommendations are offered based on the results of RCTs targeting critically‐ill patients.

The first CQ on the topic of nutrition management offers a recommendation on how to prioritize the route of administering nutritional support (i.e., enterally or parenterally). Six meta‐analyses were conducted to analyze 36 RCTs that compared the influence of enteral and parenteral nutrient delivery routes on various clinical outcomes (such as mortality rate, incidence of infection, and length of ICU/hospital stay) by targeting various disease/injury states (including external trauma, surgery, acute pancreatitis, and burns). These RCTs are also systematically reviewed in the JSICM guidelines,^287^ but only one study specifically targeted sepsis patients. As such, the literature upon which this guideline is based is limited to RCTs targeting “critically‐ill patients requiring ICU management.” Stated differently, literature sources targeting postoperative patients, and those with mild or chronic diseases were excluded.

CQ13‐2 offers a recommendation for when enteral nutritional support should be initiated. In the meta‐analysis, enteral nutrition within 24 h after ICU admission decreased mortality,^288^, ^289^, ^290^ the frequency of complication from infection,^288^, ^289^, ^290^ and length of hospital stay.^291^ However, the RCTs targeted by these meta‐analyses were small‐scale studies, and many were of low research quality. Many of the RCTs that reported observing decreased mortality rates were prone to selection bias and execution bias, and according to the results of the most recent meta‐analysis, initiating early enteral nutrition was not found to reduce mortality rates when only studies less prone to bias were examined.^292^ The studies targeted for this CQ initially included critically‐ill patients because there are few studies on sepsis. However, some of these studies included patients who were not critically ill. For this reason, studies that may have involved patients on parenteral nutrition or included patients who were not critically ill were excluded from the analysis. Sensitivity analysis was conducted with six RCTs that grouped participants who received enteral nutrition within 24 h of ICU admission into “early” groups.

CQ13‐3 offers a recommendation concerning optimal quantities for enteral feeding. The definitions of dose‐limiting groups vary among the studies on this topic, and how nutritional supplementation administered is limited must be assessed carefully. Types of dose limitation can be roughly classified as follows: (i) Low‐volume administration: refers to the so‐called trophic feeding. This method involves ~25% of daily calorie consumption or 500 kcal/day (20 kcal/hr) and is often used to maintain the intestinal mucosa or immune function. (ii) Mild calorie restriction: this method is also referred to as the so‐called permissive underfeeding or hypofeeding and calls for ~60%–70% of daily caloric consumption. This method is designed to provide slightly less than the typical daily caloric consumption to avoid oxidative stress and autophagic disorders. (iii) Standard administration: supplementation starts with a small quantity and increases to the typical daily caloric consumption over time. (iv) Administration of the typical daily caloric consumption from the beginning: an amount approximating the typical amount of calories consumed daily is administered from the start of nutritional supplementation, which can be reduced if symptoms of intolerance such as increased stomach volume or diarrhea are observed. This method is used to minimize a patient's caloric deficit as much as possible. In accordance with the rules of guideline committee, the recommendations offered in this CQ cite the latest version of the meta‐analysis conducted while formulating the JSICM guidelines. The meta‐analysis for this guideline was conducted using three distinct groups: (i) a trophic feeding group, (ii) a permissive underfeeding or hypofeeding group, and a “full feeding” group comprised of supplementation methods (iii) and (iv) together. However, there were only a few references available for each group, and no significant differences or trends were observed regardless of the outcome. As such, methods (i) and (ii) together are presented as the “underfeeding” group, which is compared with the “full feeding” group. As a result, it was also found that no significant differences exist between the groups with respect to mortality rate and the incidence of complication by infection. In the “full feeding” group, concerns regarding the risk of aspiration and the increased frequency of diarrhea increase in proportion with increases in the residual gastric volume. In addition, the potential need for renal replacement therapy also increases. Accordingly, providing enteral nutrition in quantities approximating the typical daily caloric consumption from the start is not recommended. According to a study by Rice *et al*.,^293^ while even 15% or 25% of daily caloric consumption could be sufficient to provide optimal nourishment, the body mass index (BMI) of the patients targeted was close to 30, and the average age was 53 years. This situation is often encountered when adapting evidence derived from such patients in Japanese ICUs, where many patients are elderly and have an average physique. Meanwhile, no RCTs involving malnourished patients could be found, and the optimal volume of supplementation for such patients remains unknown. However, recommendations are offered in consideration of the observation that complications tend to increase with increasing caloric deficit.^294^

CQs 13‐4 and 13‐5 offer recommendations regarding when to initiate parenteral nutrition and the optimal quantities. Stated differently, these recommendations consider the difference between energy consumed and the caloric value of enteral nutrition (i.e., whether it is necessary to supplement a caloric deficit parenterally or to administer parenteral nutrition at an early stage in cases where enteral nutrition is not possible). The discussion of when to initiate parenteral nutrition in CQ13‐4 is based on a novel meta‐analysis targeting six RCTs extracted during the formulation of the JSICM guidelines (Doig, 2013; Langouche, 2013; Heidegger, 2013; Casaer, 2011; Singer, 2011; and Bauer, 2000; please refer to the CQ comments for further details on each study).

Meanwhile, no studies targeting patients with malnutrition could be found for the corresponding CQ, and recommendations were formulated based on international guidelines and the expert opinions of members of the Guideline Committee. As no studies capable of serving as an evidentiary basis for a recommendation regarding the optimal quantity of parenteral nutrition addressed by CQ13‐5 could be found, it was suggested that the recommendation be based on three RCTs (Doig, 2013; Heidegger, 2013; and Casaer, 2011; please refer to the CQ comments for further details on each study). These RCTs influenced the recommendations offered for the corresponding topics in the JSICM guidelines, as well as one additional relevant RCT identified by conducting another literature search using the same search parameters (please refer to the CQ comments for further details on this study). The recommendation for this CQ is presented as the opinion of the Guideline Committee (expert consensus).

### CQ13‐1 Which route of nutrient delivery should be prioritized: enteral or intravenous?

#### Answer (Recommendation)

We recommend that enteral nutrition rather than parenteral nutrition in sepsis patients (1B; rate of agreement: 94.7%).

#### Rationale

Several studies targeting critically‐ill patients requiring ICU management were extracted from the JSICM guidelines. These studies included eleven RCTs concerning mortality rates^295^, ^296^, ^297^, ^299^, ^300^, ^301^, ^302^, ^303^, ^304^, ^305^, ^306^ and seven RCTs concerning infection frequency.^297^, ^298^, ^299^, ^300^, ^301^, ^302^, ^305^

Regarding potential benefits, although no improvement in mortality rate was observed in seven studies focusing on trauma, one on sepsis, one on pancreatitis, and two on surgery or serious internal illness, the benefit of enteral nutrition with respect to reduced infection frequency was confirmed in five studies focusing on trauma, one on sepsis, and one on pancreatitis. However, the study by Harvey *et al*. was not included in the analysis regarding the incidence of infection as it did not describe the incidence of all infections. Also, no complications impacting prognosis were observed, and as enteral nutrition was found to reduce the burden of medical costs as well,^307^ it was concluded that the benefits outweigh the potential risks.

### CQ13‐2 When should enteral nutrition be initiated?

#### Answer (Recommendation)

We recommend that enteral nutrition be initiated early (within 48 h) if a patient will be unable to maintain a sufficient oral caloric intake during the first several days after the onset of sepsis (1C; rate of agreement: 94.7%).

#### Rationale

Nine RCTs were extracted based on the systematic review conducted for this CQ.^308^, ^309^, ^310^, ^311^, ^312^, ^313^, ^314^, ^315^, ^316^ Mortality rate was assessed by eight RCTs; infection frequency, the length of ICU stay, and duration of ventilation were assessed by seven RCTs, and length of hospital stay was assessed by four RCTs. The risk ratio for the impact of early enteral nutrition on mortality rate was 0.9 (95% confidence interval [CI]: 0.52–1.41), the risk ratio for the impact on infection frequency was 0.7 (95% CI: 0.51–1.02), the impact on length of ICU stay was −2 days (95% CI: −5.25 to 1.18), no impact was observed with respect to length of hospital stay (0 days; 95% CI: −17.18 to 16.53), and the impact on duration of ventilation was −1 day (95% CI: −4.82 to 2.49).

Regarding potential benefits, although no differences in mortality rate, duration of ventilation, or length of hospital stay were observed based on a comparison of patients that were started on enteral nutrition within 48 h of ICU admission and patients receiving enteral nutrition after 48 h, the incidence of infections was lower in the early feeding group. In the sensitivity analysis, an additional intervention group started on enteral nutrition within 24 h of ICU admission was established, and it was found that the incidence of infection and length of ICU stay were significantly lower in the early feeding group. Regarding potential harm, no clear adverse effects were observed, and as enteral nutritional solutions are inexpensive and their use does not increase medical staff workload, it was concluded that the benefits outweigh the potential risks.

### CQ13‐3 What is the optimal amount of calories in early enteral nutrition?

#### Answer (Recommendation and opinion)

We suggest against administering an amount of calories approximating typical caloric intake during the initial ICU period (~1 week) if the patient was not malnourished before the onset of sepsis (2C; rate of agreement: 89.5%).

We suggest against limiting caloric intake in malnutrition. In this situation, nutrition should be provided while considering the risk of developing refeeding syndrome (expert consensus/no evidence).

#### Rationale

Six RCTs^317^, ^318^, ^319^, ^320^, ^321^, ^322^ included in the systematic review of the JSICM guidelines were extracted. All six RCTs reported on mortality rate, three reported on infection frequency, two RCTs reported on the duration of ventilation, the length of ICU stay, and length of hospital stay; four RCTs reported on ventilator‐associated pneumonia (VAP), and two RCTs reported on the frequency of continuous renal replacement therapy (CRRT) use. The risk ratio for the impact of caloric restriction on mortality rate was 0.93 (95% CI: 0.83–10.7), and the risk ratio for the impact on the frequency of infection was 1.08 (95% CI: 0.83–1.41). In addition, the effect of caloric restriction on duration of ventilation was −1.04 days (95% CI: −3.29 to 1.20), the effect on length of ICU stay was −1.8 days (95% CI: −4.22 to 0.86), the impact on length of hospital stay was −0.84 days (95% CI: −19.2 to 17.5), the risk ratio for the effect on VAP incidence was 0.9 (95% CI: 0.68–1.17), and the risk ratio for the effect on frequency of CRRT use was 0.64 (95% CI: 0.45–0.91).

The observed benefit was a lower frequency of CRRT administration in the calorie restricted feeding group. No clear adverse effects were observed. However, some previous studies have reported observing adverse outcomes (such as more hospital transfers than discharges) associated with low‐volume enteral nutrition (trophic feeding). In addition, enteral nutrition is inexpensive and requires small quantities of feeding solution, and as such, its potential risks are small.

### CQ13‐4 When should parenteral nutrition be initiated?

#### Answer (Recommendation and opinion)

We suggest against that the initiation of parenteral nutrition within the first week of hospitalization if there was no malnutrition prior to the onset of sepsis/septic shock. However, enteral nutrition may be initiated within 1 week of hospitalization (2D; rate of agreement: 84.2%).

We suggest that initiating parenteral nutritional supplementation should be considered while monitoring the risk of developing refeeding syndrome if malnutrition is observed before ICU admission, or enteral nutrition cannot be initiated within 1 week of hospitalization (expert consensus/no evidence).

#### Rationale

In a meta‐analysis of six RCTs extracted from the JSICM guidelines^287^ (Doig,^323^ Langouche,^324^ Heidegger,^325^ Casaer,^326^ Singer,^327^ and Bauer^328^), all six reported on mortality rate, four reported on bloodstream infection or respiratory infection, and five reported on urinary tract infection. The risk ratio for the impact of initiating parenteral nutrition within 1 week on mortality rate was 0.95 (95% CI: 0.81–1.11), the risk ratio for the impact on bloodstream infections was 1.22 (95% CI: 1.02–1.46), the risk ratio for the impact on respiratory infections was 1.07 (95% CI: 0.87–1.32), the risk ratio for the impact on urinary tract infections was 1.12 (95% CI: 0.84–1.49), and bloodstream infections significantly increased.

Although no benefits were observed in the context of this CQ, the incidence of bloodstream infections increased in the group that was started on total parenteral nutrition within 1 week. In addition, the greater cost of parenteral nutrition could place an additional burden on these patients. Based on these observations, the potential harm was determined to outweigh the potential benefits.

Selective bias in the studies adopted for this CQ was higher in those involving patients with a higher BMI compared to the Japanese patients and the group with high caloric deficit because we excluded patients in whom malnutrition was observed prior to ICU admission. In addition, the EPaNIC (Early versus Late Parenteral Nutrition in Critically Ill Adults) study accounted for 70% of the participants in both groups, more than half of whom were post‐cardiac surgery patients. The fact that many of these studies were conducted as open‐label studies has been pointed out as a source of execution bias. Thus, in light of the weak body of clinical trial evidence, it was determined that the benefits and risks of this intervention should be assessed in the clinical setting.

### CQ13‐5 What is the optimal amount of calories in parenteral nutrition?

#### Answer (Recommendation and opinion)

We suggest that the use of parenteral nutrition in cases where enteral nutrition cannot be initiated within 1 week after the onset of sepsis/septic shock or if malnutrition is observed (expert consensus/no evidence; rate of agreement: 94.7%).

We suggest against administering 100% of the target caloric value in such cases (2C). However, the optimal value remains uncertain (expert consensus/no evidence).

#### Rationale

No RCTs conforming to the PICO process could be found. Evidence for this CQ was gathered from three essential papers.^323^, ^325^, ^326^ Differences in the nutritional management discussed in these three papers were examined, and the results were used to formulate the opinions serving as a basis for the recommendation offered by this CQ (expert consensus). One more RCT^329^ was found as in an additional search. This was a small‐scale study examining 50 cases involving gastrointestinal disease. The incidence rates of sepsis in 50 consecutive cases requiring nutritional therapy for 5 days or more were compared between a group receiving 60% of the typical caloric value based on Schofield's estimation formula and a group receiving 100% of the typical caloric value. The incidence of sepsis (3 cases versus 12 cases; *P *=* *0.003), the incidence of systemic inflammatory response syndrome (SIRS; 9 cases versus 16 cases; *P *=* *0.017), and the frequency of nutrition‐related complications (2 cases versus 9 cases; *P *=* *0.016) were significantly lower in the 60% administration group. Based on these results, it is recommended that 100% of the target caloric value should not be given at least during the acute phase.

Regarding potential harm, early parenteral nutrition is expected to prevent various complications arising from an increased caloric deficit, particularly in malnutrition. However, the possibility that administering 100% of the target caloric value during parenteral nutrition may increase the risk of complication by infection could not be ruled out.

## CQ14: BLOOD GLUCOSE MANAGEMENT

### Introduction

Hyperglycemia can worsen patient prognosis by adversely affecting immune function, and increasing the risk of infection. Thus, blood glucose management is considered a crucial component of the treatment of patients with sepsis. An important adverse effect associated with insulin‐based glycemic control is hypoglycemia. As its onset can worsen the prognosis in critically‐ill patients, attending physicians must carefully consider the benefits and risks of different target levels of blood glucose control. Erroneous blood glucose measurements can also lead to the improper use of insulin.

According to the results of a single‐center randomized controlled trial (RCT) involving cardiac surgery patients in the ICU,^330^ ICU mortality declined as a result of administering intensive insulin therapy with a target blood glucose level of 80–110 mg/dL. Also, the results of a subsequent RCT involving patients whose ICU stay was estimated to be 3 days or longer indicated that intensive insulin therapy did not reduce the mortality rate of all patient groups.^331^ In the Normoglycemia in Intensive Care Evaluation and Survival Using Glucose Algorithm Regulation (NICE‐SUGAR) trial, intensive insulin therapy was demonstrated to have increased the 90‐day mortality rate.^332^ A meta‐analysis by Friedrich *et al*. reported that intensive insulin therapy was not beneficial to critically ill patients.^333^ Another meta‐analysis by Song *et al*. involving sepsis patients also showed that intensive insulin therapy carried a high risk of hypoglycemia.^334^ Given these findings, intensive insulin therapy cannot currently be recommended for sepsis patients. Based on the results of the NICE‐SUGAR trial, both the Surviving Sepsis Campaign Guidelines (SSCG) 2012 and previous guidelines recommend the initiation of insulin protocol at a blood glucose level of ≥180 mg/dL and establish a target blood glucose level of 144–180 mg/dL.^2^, ^29^

However, although there have been numerous comparisons of target blood glucose values ≤110 mg/dL with values ≥180 mg/dL among the various RCTs comparing the impact of target blood glucose levels on mortality rates, there is little direct evidence comparing other target blood glucose ranges. Therefore, the optimal range of these two target blood glucose levels (110–144 and 144–180 mg/dL) was unknown. Because of this, the network meta‐analysis (NMA) method was used to conduct indirect comparisons to be examined when direct comparisons did not exist to determine which of four different target blood glucose ranges: ≤110, 110–144, 144–180, or ≥180 mg/dL, was optimal in terms of balance of clinical benefits and potential harm.

The incidence of in‐hospital mortality and hospital infections did not differ in direct comparisons. NMA revealed that the 144–180 mg/dL range was significantly lower than ≥180 mg/dL with respect to mortality rate. The incidence of hypoglycemia, a potential adverse effect of blood glucose control, was significantly higher for the ≤110 and 110–144 mg/dL ranges in direct comparison to the 144–180 and ≥180 mg/dL ranges. As a result of the NMA, no significant differences were observed between the 144–180 and ≥180 mg/dL ranges. We recommend a target blood glucose range of 144–180 mg/dL based on these findings.

Blood glucose measurements in the ICU are often taken using a glucometer or an arterial blood gas analyzer. However, the results may differ depending on the device used as well as the blood sampling method. Glucometers are used in many ICUs, but because this method of measurement is frequently inaccurate and often yields an overestimation, it is possible that the onset of hypoglycemia may be overlooked.^335^ Glucometer measurements using capillary blood are significantly less accurate in comparison to glucometer measurements using venous blood or a blood gas analyzer.^336^ Erroneous glucometer measurements using capillary blood is a clinically significant problem particularly in hypoglycemia cases (blood glucose level ≤72 mg/dL), and blood glucose measurements taken with blood gas analyzers are more accurate.^335^ Regarding measurement time, the use of an arterial blood gas analyzer is recommended as well as the use of glucometer with arterial/venous blood. We do not recommend the use of glucometer with capillary blood. However, measurement errors can occur even with these recommended methods, and thus measurements should be taken at a central laboratory as appropriate in order to confirm accuracy.

### CQ14‐1 What is the optimal blood glucose target level in patients with sepsis?

#### Answer (Recommendation)

We suggest an optimal target blood glucose range of 144–180 mg/dL in patients with sepsis (2C; rate of agreement: 100%).

#### Rationale

Our analysis included 14,495 patients from 27 studies.^128^, ^330^, ^331^, ^332^, ^337^, ^338^, ^339^, ^340^, ^341^, ^342^, ^343^, ^344^, ^345^, ^346^, ^347^, ^348^, ^349^, ^350^, ^351^, ^352^, ^353^, ^354^, ^355^, ^356^, ^357^, ^358^, ^359^ None of the studies were especially prone to bias. We performed an NMA in addition to a direct comparison because there were no studies that directly compared patients with a target blood glucose level >180 mg/dL to those with a target blood glucose level of 144–180 mg/dL.

The direct comparison revealed that compared with target blood glucose levels of 144–180 and >180 mg/dL, target blood glucose levels of 110–144 mg/dL were associated with a significantly higher risk of hypoglycemia; however, there were no significant differences in the risk of mortality and infection.

The NMA showed that target blood glucose levels of 144–180 mg/dL were associated with a significantly lower risk of hospital mortality and sepsis or bloodstream infection compared with blood glucose levels >180 mg/dL, and there were no significant differences in the risk of hypoglycemia between the target blood glucose levels of 144–180 and >180 mg/dL.

The quality of evidence in the direct comparison was B (moderate) to C (weak), and that in the NMA was C (weak) to D (very weak). Finally, we decided that the quality of evidence was C (weak).

Reduction of hospital mortality and infection is considered a benefit of blood glucose management. Our NMA showed that compared with the target blood glucose levels >180 mg/dL, levels of 144–180 mg/dL were associated with a significantly lower risk of hospital mortality and sepsis. Furthermore, there were no significant differences between the target blood glucose levels <110 and 110–144 mg/dL with regard to these outcomes. The increase in the incidence of hypoglycemia is considered an adverse effect of blood glucose management.

Therefore, we assessed the balance between the risk and benefit of blood glucose management as follows: (i) Target blood glucose levels <110 and 110–144 mg/dL – equilibrium or uncertain, (ii) Target blood glucose levels of 144–180 mg/dL – benefit may exceed risk, (iii) Target blood glucose level >180 mg/dL – risk may exceed benefit.

### CQ14‐2 What devices/instruments should be used to measure blood glucose in sepsis patients?

#### Answer (Recommendation)

We recommend against the use of a glucometer with capillary blood in patients with sepsis (1B; rate of agreement: 94.7%).

We suggest the use of a glucometer with arterial/venous blood (2B) and recommend the use of an arterial blood gas analyzer (1C) in patients with sepsis (rate of agreement: 94.7%).

#### Rationale

The recommendation presented for this CQ was based on the results of a systematic review conducted by Inoue *et al*.^336^ In a comparison of measurements taken using a glucometer with capillary blood or a blood gas analyzer using arterial blood, measurements taken using the arterial blood gas analyzer were less likely to include measurement errors falling significantly outside the acceptable range (odds ratio: 0.04; 95% confidence interval [CI]: 0.01–0.14).^336^ In addition, in a comparison of glucometer measurements using capillary blood or arterial blood, the risk of measurement error was significantly lower when arterial blood was used (odds ratio: 0.36; 95% CI: 0.25–0.52). No significant difference was observed between glucometer measurements using arterial blood and blood gas analyzer (arterial blood), but measurement errors tended to be lower when the blood gas analyzer (arterial blood) was used (odds ratio: 0.17; 95% CI: 0.01–2.46).^336^

Regarding the benefit‐risk balance, it was determined that (i) the potential risks of glucometer (capillary blood) use clearly outweighed the benefits, (ii) the benefits of glucometer (arterial/venous blood) use likely outweighed the potential risks, and (iii) the benefits of using a blood gas analyzer (arterial blood) clearly outweighed the potential risks.

## CQ15: BODY TEMPERATURE REGULATION

### Introduction

Body temperature is an important indicator of the general condition of the body. It is not uncommon to initiate new medical treatment after observing hypothermia or fever.^360^ Fever is caused by endogenous interleukin‐1 and tumor necrosis factor‐α, which are produced in response to exogenous stimuli, and promote cyclooxygenase‐mediated prostaglandin E2 production in the arachidonic acid cascade.^361^ Fever is an important indicator of the presence of infection, but can also be caused by factors other than infection, such as surgery,^362^ blood transfusion,^363^ drug therapy,^364^, ^365^ and acute rejection.^366^ In addition, there are often multiple causes of fever in critically‐ill patients. In the Fever and Antipyretic in Critically ill patients Evaluation (FACE) study, a multicenter prospective observational study conducted in 25 facilities located in Japan and South Korea, fever over 38.5°C occurred in 40.5% of ICU patients, and fever over 39.5°C occurred in 11.5% of ICU patients.^367^ Fever causes patient discomfort, increased respiratory demand and myocardial oxygen demand,^368^ and central nervous system disorders. However, fever is also a defensive reaction that increases antibody production, T‐cell activation, cytokine synthesis, and activation of neutrophils and macrophages.

The heart rate and oxygen consumption can both be expected to decrease if body temperature decreases as a result of antipyretic therapy for fever. Antipyretic therapy is generally administered to critically‐ill patients to minimize discomfort and reduce minute ventilation. Although antipyretic therapy may be given to reduce or prevent fever‐related adverse events, this type of therapy is routinely administered to relieve the fever itself.^367^ However, antipyretic therapy may also suppress beneficial self‐defense reactions. In addition, side effects may occur, such as gastrointestinal, liver, or kidney disorders, among others.^369^

The first Clinical Question (CQ) on this topic is “Should antipyretic therapy be administered to sepsis patients presenting with fever?” Few studies have assessed the impact of antipyretic therapy on patient prognosis. Analyzing subgroups of patients treated via external cooling or antipyretic medication was not possible. In addition, the threshold of body temperature for initiating antipyretic therapy varies, and there is still no consensus regarding the body temperature at which antipyretic therapy should be initiated. At the very least, the uniform administration of antipyretic therapy as a standard treatment in adhering to the “initiate antipyretic therapy after body temperature reaches 38.5°C or higher” approach is considered to be undesirable.

Reduction in the body temperature of patients with sepsis is believed to be caused by the loss of the body's capacity to regulate temperature, sedation/muscle relaxation, or extracorporeal circulation. This phenomenon is more likely to occur in severe cases than those with fever. The definitions of Acute Physiologic Assessment and Chronic Health Evaluation II (APACHE II) score,^370^ sepsis,^371^ and Infection‐related Ventilator‐Associated Complication (IVAC)^372^ each specify temperature values <36°C as abnormal. It has also been reported based on the results of an analysis of the Japanese Sepsis Registry that sepsis patients who developed hypothermia with body temperatures below 36.5°C within 24 h of hospitalization had a high rate of mortality.^373^

The second CQ presented on this topic is “Should we attempt to correct the body temperature of hypothermic sepsis patients?” There have been no studies conducted to date that examine the influence of body temperature correction on prognosis in hypothermic sepsis patients. In addition, to carry out an interventional study in patients with hypothermia to compare (i) leaving patients to recover naturally and (ii) correcting body temperature would be unethical. Attending physicians should carefully monitor patients recovering from hypothermia since they may become hemodynamically unstable. Thus, physicians should consider a balance between the adverse effects of hypothermia itself and the risk associated with body temperature correction. When necessary, body temperature should be corrected gradually through methods such as extracorporeal circulation, passive incubation, covering the patient with a blanket, etc.

### CQ 15‐1: Should antipyretic therapy be administered to sepsis patients presenting with fever?

#### Answer (Recommendation)

We suggest against the routine use of antipyretic therapy for septic patients presenting with fever (2C; rate of agreement: 94.7%).

Comment: Administering antipyretic therapy may be considered as a method of alleviating physiological responses accompanying fever, such as tachycardia, tachypnea, and discomfort.

#### Rationale

Six randomized controlled trials (RCTs) were extracted as a result of the systematic review conducted for this CQ.^374^, ^375^, ^376^, ^377^, ^378^, ^379^ All six RCTs assessed mortality rates, one assessed ICU‐free survival days, four examined the length of ICU stay, and two examined the rates of complication from infection.

The risk ratio for the impact of antipyretic therapy on mortality rate was 1.12 (95% confidence interval [CI]: 0.83–1.51), the impact on the number of ICU‐free survival days was +1 day (95% CI: −0.38 to 2.38), and the risk ratio for the impact on the length of ICU stay was −0.04 days (95% CI: −0.76 to 0.68). Regarding the incidence of infection, one RCT reported the frequency of infection and another RCT reported the number of patients who developed infections; thus, it was not possible to compare the two.

In this CQ, mortality rate, ICU‐free survival days, and length of ICU stay were assessed as benefits of antipyretic therapy, and the incidence of infectious complications was assessed as a potential harm associated with antipyretic therapy. The strength of the evidence concerning the primary outcome of mortality rate was C (weak). While the strength of the evidence regarding ICU‐free survival days and length of ICU stay was B (moderate strength), there was no consistent assessment method for the potential harm of complication by infection, and evaluating the strength of this evidence was not possible. The strength of the evidence regarding all outcomes was C (weak) in keeping with the strength of the evidence on mortality rate as the primary outcome.

The risk ratio for the impact of antipyretic therapy on mortality rate was 1.12 (95% CI: 0.83–1.51), but the extent to which it reduces mortality is unclear. The results of one RCT suggested that ICU‐free survival days may increase by ~1 day. No significant shortening in length of ICU stay was observed. The results of two other RCTs indicated the clear potential for an increased risk of complication by infection as a result of antipyretic therapy. Accordingly, the balance of risk and benefit of administering antipyretic therapy for septic patients with fever is still uncertain. In addition, the use of antipyretic drug therapy or external cooling can be expected to increase the workload of medical staff.

### CQ15‐2 Should we attempt to correct the body temperature of hypothermic sepsis patients?

#### Answer (Opinion)

We suggest that the correction of body temperature while carefully considering hemodynamic stability for hypothermic sepsis patients with hypothermia‐related complications such as diminished cardiac contractility/dilatability or coagulation abnormalities (expert consensus; rate of agreement: 100%).

#### Rationale

There were no RCTs conforming to the Patient, Intervention, Comparison, Outcome (PICO) process. Attempts to gradually correct patient body temperature in cases where diminished cardiac contractility/dilatability or coagulation abnormalities occur in hypothermia^380^, ^381^ are believed to offer potential benefits when such symptoms are determined to be hypothermia‐related. However, carefully monitoring for possible hemodynamic instability due to factors such as decreased blood pressure or the relative decrease in circulating blood volume is necessary while patients recover from hypothermia.

The benefit‐risk balance can differ depending on the condition of the patient. Attending physicians should determine whether to act while considering both the adverse effects of hypothermia itself as well as the risk of attempting to correct body temperature and if treatment is necessary, body temperature should be corrected gradually through methods such as extracorporeal circulation, passive incubation, or covering the patient with a blanket.

## CQ16: DIAGNOSIS AND TREATMENT OF DISSEMINATED INTRAVASCULAR COAGULATION (DIC) IN PATIENTS WITH SEPSIS

### Introduction

#### Coagulation and fibrinolytic changes in sepsis

In the clinical course of sepsis, coagulation/fibrinolytic abnormalities are identified early on, and the risk of death due to multiple organ failure increases markedly when complicated by disseminated intravascular coagulation (DIC).^382^, ^383^ DIC in sepsis manifests primarily as a state of severe systemic activation of the blood coagulation cascade, and it is believed that microcirculatory damage caused by intravascular coagulation can become a cause of organ damage.^384^ In DIC, although fibrinolytic function also increases in response to the activation of the coagulation cascade, the extent of this increase varies depending on the underlying disease, and thus DIC can occur with suppressed fibrinolysis or with enhanced fibrinolysis. Of these, DIC occurring in sepsis exhibits typical fibrinolytic suppression patterns in which fibrinolytic function is suppressed relative to the enhancement of coagulation.^385^ In addition, DIC with suppressed fibrinolysis is considered to have a poor prognosis, particularly when complicated by multiple organ disorders.^385^

#### Necessity of diagnosing DIC in sepsis

There are two factors underlying the significance of evaluating the coagulation/fibrinolytic status of patients with sepsis: (i) gaining an accurate grasp of the patient's disease state, and (ii) determining the need for therapeutic intervention.^386^ The results of numerous studies have indicated that DIC is linked with poor prognosis in sepsis,^387^ and diagnosing DIC is necessary to predict outcomes and to determine the timing of interventions. As anticoagulant therapy carries a risk of excessive bleeding, selecting appropriate cases and applying rationally planned timing are essential.^382^ Administering anticoagulants inappropriately will not only fail to produce the desired results but can also increase patients’ risk of adverse events.^388^ Therefore, when treating patients with sepsis, attending physicians should acquire a sufficient grasp of the state of coagulation/fibrinolytic functions in real‐time and perform suitable interventions after the diagnosis of DIC.

#### Usefulness of anticoagulant therapy for sepsis‐associated DIC

Many anticoagulants have been evaluated to date in connection with sepsis‐associated DIC based on the understanding that excessive coagulation activity can cause microcirculatory damage leading to organ failure.^389^ However, there is currently no unified view regarding the efficacy of these drugs. One reason for this is that in the United States and European countries, the clinical utility of various anticoagulants has been studied primarily in large‐scale randomized controlled trials (RCTs) involving patients with severe sepsis.^390^, ^391^ These studies did not focus on sepsis‐associated DIC, and the anticoagulant therapies administered to the participants of these studies clearly differed from those used in Japan. According to the results of a recent meta‐analysis, anticoagulants cannot be expected to be effective in general sepsis cases, and their effectiveness is reported to be limited to the treatment of sepsis‐associated DIC.^387^ This guideline was formulated based on the entirety of available data, although the current body of evidence regarding anticoagulant therapy for sepsis‐associated DIC is still limited in both quality and quantity.

### CQ16‐1 Is the Japanese Association for Acute Medicine DIC diagnostic criteria useful in diagnosing sepsis‐associated DIC?

#### Answer (Opinion)

The Japanese Association for Acute Medicine (JAAM) DIC diagnostic criteria have been recognized as valid both as treatment initiation criteria and useful as an index of severity, and are also considered useful in diagnosing sepsis‐associated DIC (expert consensus/no evidence; rate of agreement: 94.7%).

#### Rationale

The question of what diagnostic criteria should be used for the diagnosis of sepsis‐associated DIC is frequently encountered in routine practice, and the current state of this issue on the background of multiple diagnostic criteria in use is addressed by this CQ. Given the fact that the JAAM DIC diagnostic criteria (hereinafter referred to as “acute‐phase criteria”) created in 2005 by the Research Committee on DIC of the JAAM have gained widespread acceptance in Japan, this CQ focuses the discussion on the clinical utility of the acute‐phase criteria.

The main diagnostic criteria currently in use are the Japanese Ministry of Health, Labour and Welfare DIC Diagnostic Criteria,^392^ the International Society on Thrombosis and Hemostasis (ISTH) overt DIC Diagnostic Criteria,^393^ and the acute‐phase criteria^382^ created by the JAAM.

It is impossible to discuss the superiority or inferiority of these diagnostic criteria, as there is no “correct diagnosis” for DIC. The acute‐phase criteria was formulated for the purpose of facilitating early‐stage DIC diagnoses, of the 3 diagnostic criteria, DIC should be diagnosed for the most widespread coagulopathies.^382^, ^394^ This criterion has become the most widely‐used diagnostic criterion for sepsis‐associated DIC in Japan because of the simplicity of its associated diagnostic procedures.

The acute‐phase criteria are considered to be useful for diagnosing sepsis‐associated DIC because they have been recognized as valid both as treatment initiation criteria^395^ and also suitable as an index of disease severity.^396^ The acute‐phase criteria are considered to be useful for diagnosing sepsis‐associated DIC and appear to present minimal risks. As such, it is believed that the potential benefits likely outweigh the potential risks.

### CQ16‐2 Should recombinant thrombomodulin be administered to patients with sepsis‐associated DIC?

#### Answer (Opinion)

No clear recommendation can be offered at this time concerning the use of recombinant thrombomodulin preparations in sepsis‐associated DIC (expert consensus/quality of evidence “B” ; a rate of agreement of 67% or higher in support of its use could not be obtained).

#### Rationale

Recombinant thrombomodulin, which was first launched in 2008, is one of the several anticoagulants that have come into wide use in treating sepsis‐associated DIC in Japan. However, to date, no consensus has been reached regarding its usefulness. Although the bulk of clinical evidence regarding this drug has been the product of a Japanese phase III trial^397^ and a multiregional Phase II trial,^398^ the scales of these studies were determined to have been inadequate, and no recommendation was offered. Another multiregional phase III trial is currently in progress with results expected in 2018, and this Clinical Question (CQ) was formulated based on the currently available body of evidence in view of the importance of clarifying the potential benefits and risks of recombinant thrombomodulin use in sepsis‐associated DIC treatment. An existing systematic review (Yamakawa *et al*., 2015)^399^ was used to formulate this recommendation.

The systematic review for this CQ was conducted using 3 RCTs^398^, ^400^, ^401^ as mentioned previously. The quality of evidence was “B” (moderate), and it was determined that the potential benefits likely outweigh the potential risks. The estimated effect of this treatment intervention on mortality rate was represented by a risk ratio (RR) of 0.81 (95% confidence interval [CI]: 0.62–1.06), and the point estimate for the number needed to treat was 15, with moderate benefits expected. The estimated effect on hemorrhagic complications was represented by an RR of 0.83 (95% CI: 0.22–3.11), and it was determined that there was a low risk that the treatment intervention increases the frequency of hemorrhagic complications. Meanwhile, a study by Aikawa^400^ comparing thrombomodulin therapy against heparin as a control was excluded from analysis due to the possibility that its evaluation of hemorrhagic complications may not have been appropriate. As a result, the RR was 1.11 (95% CI: 0.59–2.11), and the possibility that thrombomodulin may cause a slight increase in the frequency of hemorrhagic complications could not be eliminated.

The potential benefits of treatment intervention in this CQ were evaluated in terms of its capacity to improve mortality rate. Although the possibility of increased frequency of hemorrhagic complications as a result of thrombomodulin therapy could not be ruled out, it was determined to be highly likely that the potential benefits of its use outweigh the potential risks. However, there are different viewpoints regarding how to interpret the benefit‐risk balance, and no consensus could be reached as some committee members emphasized that no significant differences in beneficial outcomes were observed. It was decided that no recommendation would be offered at this time in anticipation of the results of the aforementioned ongoing phase III trial in 2018.

### CQ16‐3 Should antithrombin replacement therapy be administered in sepsis‐associated DIC?

#### Answer (Recommendation)

We suggest the use of antithrombin replacement therapy in patients with sepsis‐associated DIC whose antithrombin activity has decreased to ≤70% (2B; rate of agreement: 68.4%).

#### Rationale

Guidelines for use in various countries do not recommend the use of antithrombin based on the results of a large‐scale clinical trial (the KyberSept trial).^390^ In contrast, antithrombin replacement therapy is often used in patients with sepsis‐associated DIC in Japan. On this background, this guideline discusses the clinical utility of antithrombin replacement therapy based on a repeat analysis of patients with sepsis‐associated DIC.

This analysis targeted studies limited to patients with sepsis‐associated DIC. The KyberSept trial^390^ assessed severe sepsis rather than sepsis‐associated DIC, and so could not be adopted as evidence for this CQ, as it does not consider treatments for sepsis patients without DIC. Thus a post‐hoc analysis in a study by Kienast *et al*.,^402^ which was limited to sepsis‐associated DIC, was adopted. Meanwhile, the efficacy and risks of the dosage covered by the National Health Insurance for the intervention itself (1,500 units/day for patients with sepsis‐associated DIC with antithrombin activity levels of 70% or lower, and 40–60 units/kg in surgical cases) were also assessed.

In addition, four Japanese and overseas studies^395^, ^402^, ^403^, ^404^ were analyzed. The treatment intervention was expected to be beneficial in terms of reduced mortality rate based on an RR of 0.68 (95% CI: 0.49–0.93), but bias risk/indirectness may lower the reliability of the effect estimate. Although the estimated effect on hemorrhagic complications was represented by an RR of 1.17 (95% CI: 0.45–3.01) and the possibility of harm could not be eliminated, the confidence interval was wide, and thus the reliability of this finding was low.

The benefit of this treatment intervention was evaluated in terms of its capacity to reduce mortality. Although the possibility of increased frequency of hemorrhagic complications as a result of antithrombin replacement therapy could not be ruled out, it was determined to be highly likely that the potential benefits of use outweigh the potential risks.

### CQ16‐4 Should protease inhibitors be administered to patients with sepsis‐associated DIC?

#### Answer (Opinion)

We suggest against the use of protease inhibitors as standard treatment in sepsis‐associated DIC (expert consensus/quality of evidence “D”; rate of agreement: 89.5%).

#### Rationale

The body of evidence concerning the usefulness of protease inhibitors is poor, and only two RCTs^404^, ^405^ have been conducted to date. However, the results of these studies are currently referred to frequently in Japan. On this background, this guideline offers a discussion of the results of an analysis of the clinical utility of protease inhibitors in patients with sepsis‐associated DIC.

There have been two RCTs that have evaluated the utility of protease inhibitors in sepsis‐associated DIC; Hsu *et al*.^405^ reported a lack of utility, while Nishiyama *et al*.^404^ reported a potential utility. However, both reports were based on small‐scale studies that were not double‐blinded. Although these studies also assessed 28‐day mortality rate, they did not assess the frequency of hemorrhagic complications or the DIC recovery rate. No significant improvements in mortality rates were observed in these studies, and it was concluded that no prognostic improvement effect attributable to protease inhibitors could be determined, and at present, the available evidence is insufficient to support a recommendation. In addition, although the RCT conducted by Nishiyama *et al*.^406^ included trauma patients, it was ultimately decided that the results of this study were not suitable for adoption as evidence for this CQ due to a lack of sub‐analysis results.

The benefits of the treatment intervention were considered in terms of its ability to improve mortality rate, but the confidence intervals in the results were determined to contain major inaccuracies. Hemorrhagic complications were raised as a potential harm associated with this intervention and a major outcome, but the two RCTs extracted did not assess this outcome. As such, it was determined that the risks of this intervention could not be assessed and thus its benefit‐risk balance is uncertain.

### CQ16‐5 Should heparin or heparin analogs be administered in sepsis‐associated DIC?

#### Answer (Opinion)

We suggest against the use of heparin or heparin analogs as a standard treatment in sepsis‐associated DIC (expert consensus/quality of evidence “D”; rate of agreement: 84.2%).

#### Rationale

As mentioned previously, no conclusions have been reached regarding the utility of anticoagulant therapy for sepsis‐associated DIC. This assessment is particularly difficult because heparin and heparin analogs are often administered to sepsis patients to prevent deep vein thrombosis regardless of the presence of DIC. Thus, to verify the clinical efficacy of heparin/heparin analogs as treatments for sepsis‐associated DIC, this CQ discusses the results of an assessment limited to patients with sepsis‐associated DIC.

28‐day mortality rate, hemorrhagic complications, and DIC recovery rate were adopted as outcomes. Three RCTs^400^, ^407^, ^408^ conforming to the Patient, Intervention, Comparison, Outcome (PICO) process were selected to be included in the analysis. Of these three RCTs, unfractionated heparin was used in the study by Aikawa *et al*.^400^ as a control for recombinant thrombomodulin, while in the study by Aoki *et al*.,^407^ unfractionated heparin was used as a control for activated protein C concentrate. A report by Liu *et al*.^408^ focused on patients with sepsis‐associated pre‐DIC as defined in the Chinese diagnostic criteria. The 28‐day mortality rate statistics were reported by Aikawa *et al*.^400^ and Liu *et al*.,^408^ and the quality of evidence was determined to be “D” (very weak) as a result of the meta‐analysis.

Statistics pertaining to hemorrhagic complications were reported by Aikawa *et al*.^400^ and Aoki *et al*.,^407^ and the quality of evidence was determined to be “D” (very weak) as a result of the meta‐analysis. Only Aikawa^400^ reported on DIC recovery rate, and the quality of this evidence was also determined to be “D” (very weak) as a result of the meta‐analysis.

It was determined that the treatment intervention had the beneficial effect of improving the mortality rate, but the supporting evidence was found to be inadequate. Hemorrhagic complications were cited as potential serious adverse outcomes of this intervention, and it was concluded that the available evidence was insufficient to support a recommendation regarding hemorrhagic complications. In addition, although beyond the scope of the meta‐analysis, heparin‐induced thrombocytopenia (HIT) is a known complication associated with heparin use, and caution is required when administering heparin. Accordingly, it was concluded that the benefit‐risk balance for this intervention is currently uncertain.

## CQ17: VENOUS THROMBOEMBOLISM (VTE) COUNTERMEASURES

### Introduction

The clinical importance of the “prevention of venous thromboembolism (VTE)” was highlighted during the public comment stage for this clinical question (CQ) while formulating this guideline. VTE includes both deep vein thrombosis (DVT) and pulmonary embolism (PE). Although initially considered common in European and American populations, the prevalence of VTE in Japan has increased in recent years due to the westernization of lifestyle habits, an ageing population, greater awareness of the condition, and advancements in diagnostic techniques.^409^ The actual pathogenesis of VTE tends to begin following surgery, after child birth, or during hospitalization for an acute illness, and can result in serious outcomes such as PE. As such, the prevention, diagnosis, and treatment of VTE are of clinical concern. The VTE team accordingly assessed through a review of the literature whether the risk of developing VTE in sepsis patients is actually higher than in other acute conditions.

Only one recent report by Kaplan *et al*. (2015)^410^ was found as a result of a literature search for papers regarding VTE in sepsis patients. This paper discussed the results of a multicenter prospective study on the incidence of VTE based on venous echocardiography examinations of patients with sepsis or septic shock admitted to the intensive care unit (ICU), and reported that although measures to prevent VTE were taken in all cases, the incidence of VTE was high (42 of 113 cases [37.2%]). Although the results of this paper alone cannot be applied to general clinical practice in Japan, preventing and treating VTE is widely recognized as having great importance in the clinical management of sepsis patients. Based on the above, it was determined that presenting a view applicable to the medical care environment in Japan was needed, and this guideline addresses several clinically important CQs related to the occurrence of VTE in patients with sepsis.

One recent development of note was the inclusion of a section on DVT prophylaxis in the Surviving Sepsis Campaign Guidelines (SSCG) 2012, which calls for the prevention of VTE.^23^ The discussion provided in this section mentions reports indicating that the risk of VTE is higher in ICU patients,^411^ and that sepsis patients are believed to have an equal or higher risk of developing VTE compared to general ICU patients. Francesco *et al*. examined the relationship between patients with acute illnesses and the prevalence of VTE based on a review of the relevant literature published after SSCG 2012, and as a result, asymptomatic DVT was reported in 4.7% of cases, symptomatic DVT in 0.99%, PE in 0.6%, and DVT‐related death occurred in 1.9% of cases. It was also reported that for all patients, only the development of acute infection was positively correlated with the onset of VTE.^412^ Tichelaar *et al*. also demonstrated that the relative risk of VTE rises to 1.9–2.7 during pneumonia and to 1.8–2.1 during a urinary tract infection, in comparison to patients without infection.^413^ In contrast, a study of the perioperative period conducted by Donze *et al*. compared patients presenting with either systemic inflammatory response syndrome (SIRS) or sepsis prior to surgery against patients without SIRS, and reported an increase in the adjusted odds ratio for postoperative thrombotic complications to 3.3. When viewed in terms of disease severity, the risk of thrombosis increased in tandem with the severity of sepsis; the odds ratio was 2.6 in SIRS patients, 3.7 in patients with typical sepsis, and 6.1 in patients with severe sepsis.^414^

As described above, the risk of VTE is considered to be high in the presence of any infection, even if not associated with sepsis, and methods used to prevent and diagnose VTE are considered to be of great clinical importance.

For this CQ, the VTE team formulated and addressed the questions “Should anticoagulant therapy, compression stockings, and/or intermittent pneumatic compression be used to prevent DVT in sepsis patients?” (CQ17‐1) and “How should sepsis‐associated DVT be diagnosed?” (CQ17‐2), and provide the results of their discussions.

There have been virtually no report of VTE cases in Japan limited only to sepsis patients, and this is expected to be one of the various issues to be clarified in the future to ensure that appropriate preventive and diagnostic procedures are implemented.

### CQ17‐1 Should anticoagulant therapy, compression stockings, and/or intermittent pneumatic compression be used to prevent DVT in sepsis patients?

#### Answer (Opinion)

We suggest the use of anticoagulant therapy, compression stockings, and/or intermittent pneumatic compression for the prevention of DVT in accordance with a patient's risk level (expert consensus/no evidence; rate of agreement: 94.7%).

#### Rationale

VTE affecting inpatients and postoperative patients is widely recognized as a complication requiring preventative measures. In Japan, the Guidelines for the Diagnosis, Treatment and Prevention of Pulmonary Thromboembolism and Deep Vein Thrombosis (Revised 2009)^409^ and the Guidelines for Preventing Pulmonary Thromboembolism/Deep Vein Thrombosis (Venous Thromboembolism),^415^ offer a classification of various risk factors for developing VTE as well as corresponding prophylactic measures. Among them, severe infection is classified as a moderate risk factor for VTE and establishing a patient's risk level based on the underlying disease and clinical history are recommended. SSCG 2012^23^ recommends the administration of low molecular weight heparin (grade 1B) or unfractionated heparin (grade 2C) in addition to intermittent air compression of the lower extremities (grade 2C) for prophylaxis against DVT. However, the recommendations offered by these guidelines are based on studies not limited to sepsis patients, as well as data from postoperative or critically‐ill patients admitted to the ICU. The analysis conducted for this guideline was limited to sepsis patients to allow for the assessment of DVT prevention measures in such patients.

While no studies limited only to sepsis patients were found as a result of the literature search, DVT prophylactic measures are expected to prevent both pulmonary thromboembolism and mortality in sepsis patients, similar to other critically‐ill patients. However, there is currently no evidence limited only to sepsis patients, and the frequency and severity of adverse effects are unknown. The potential risks associated with anticoagulants include hemorrhagic complications and heparin‐induced thrombocytopenia (HIT), and care must be taken with their use. Compression stockings and intermittent pneumatic compression should be used with caution in patients with arterial blood flow disorders such as diabetes, due to the associate risk of exacerbation of circulatory disorders. However, because the interventions addressed in this CQ include intravenously administered pharmacologic therapies, compression stocking use, and intermittent pneumatic compression, the intervention itself creates little physical burden on patients. Based on the above, the potential benefits of VTE prevention measures clearly outweigh the risks.

### CQ17‐2 How should sepsis‐associated DVT be diagnosed?

#### Answer (Opinion)

We suggest diagnosing DVT using techniques such as clinical symptoms, D‐dimer fluctuations, venous compression ultrasonography findings, contrast‐enhanced computed tomography scan etc. assessing risk factors and adverse effects (expert consensus/no evidence; rate of agreement: 100%).

#### Rationale

Because no evidence limited only to sepsis patients was found as a result of the literature search, an expert consensus about general DVT diagnostic methods, such as the use of risk factors, clinical symptoms, D‐dimer values, and imaging, is presented.

##### Risk factors/clinical symptoms

Risk factors such as age, history of VTE, malignant tumor presence, prolonged bedridden periods, obesity, pregnancy, trauma, spinal cord injury, surgery, and cerebrovascular disorders should be assessed in the patient's medical history. Additional risk factors to be considered in sepsis cases include sedation, vasopressor use, a history of artificial respiration, central venous catheter placement. Clinical symptoms potentially indicative of acute DVT of the lower extremities include local tenderness, swelling, pitting edema, and skin discoloration of the affected limb. The diagnosis of DVT based on symptoms is difficult in a sedated sepsis patient; diagnoses based on signs are also difficult due to the generalized edema.

##### D‐dimer value

Sepsis patients often present with high D‐dimer values accompanying disseminated intravascular coagulation, and ruling out DVT based only on D‐dimer values can be difficult. However, actively monitoring for DVT onset in cases involving prolonged elevation or repeated spikes in D‐dimer values during treatment is considered to be good practice.

##### Imaging diagnostics

1. Venous compression ultrasonography: While a relatively simple bedside test, venous compression ultrasonography can be difficult to perform when the subcutaneous tissue is thickened as a result of edema, which can block ultrasonic waves.
2. Venography: Originally the gold standard for the diagnosis of DVT, venography is now considered unsuitable for routine examination because it is invasive.^416^
3. Computed tomography venography (CTV): Performing CTV can be difficult in sepsis cases due to the need for the use of a contrast agent and patient movement. According to other Japanese guidelines, CTV should be performed in patients in whom venous ultrasonography is difficult, and PE is suspected, and is similarly indicated for sepsis patients.^409^
4. Magnetic resonance imaging (MRI): Although noninvasive, routinely performing MRI is not recommended for the diagnosis of severe DVT in septic patients, due to the time required for performing an MRI and the risk of encountering difficulties or making observations.

Patients with sepsis are at a high risk of developing VTE, but it should be noted that their clinical symptoms are easily masked by factors such as sedation. As a result, attending physicians should check for associated features such as elevated D‐dimer values. The early diagnosis and treatment of DVT is considered to have a high potential for benefiting patients. In contrast, the use of contrast agents is contraindicated in sepsis cases accompanied by cardiac and renal dysfunction, and the burden on the patient should be carefully evaluated in cases involving artificial respiration or continuous apheresis due to the risks involved in transporting patients. Accordingly, the benefit‐risk balance may also vary depending on the condition of the patient.

## CQ18: ICU‐ACQUIRED WEAKNESS (ICU‐AW) AND POST‐INTENSIVE CARE SYNDROME (PICS)

### Introduction

The concepts of ICU‐acquired weakness (ICU‐AW) and Post‐Intensive Care Syndrome (PICS) were first proposed by the Society of Critical Care Medicine (SCCM)^417^ in 2010, on the backdrop of the increasing attention on somatic and psychological issues appearing during the subacute and chronic periods after discharge from the intensive care unit (ICU). ICU‐AW is a condition manifesting as acute symmetrical limb muscle weakness after ICU admission. PICS is a disorder of motor, cognitive, and neurological functions occurring during ICU admission or at the time of discharge, and in some cases even sometime after discharge. Both conditions are increasingly gaining wide recognition as affecting not only the long‐term prognosis of ICU patients but also psychologically affect their families. Recent reports have stated that subacute and chronic conditions such as PICS and ICU‐AW are closely linked with severe sepsis patients in the ICU, and the Japanese Clinical Practice Guidelines for Management of Sepsis and Septic Shock 2016 also devote separate chapters to these conditions. This guideline presents an overview of these conditions as well as respective clinical questions (CQs) concerning their diagnosis and prevention based on systematic reviews of the most recent relevant literature.

### ICU‐acquired weakness

Pathologies manifesting as acute symmetrical limb muscle weakness after ICU admission due to serious illnesses such as sepsis are attracting increasing attention.^418^ This concept is referred to as ICU‐AW and encompasses diffuse muscle weakness syndrome caused by critical illness polyneuropathy (CIP) and critical illness myopathy (CIM). It has been reported that ICU‐AW occurs in 46% of critically‐ill patients who present with sepsis, multiple organ failure, and need long‐term artificial respiration.^419^ According to a detailed assessment, the most frequently occurring type of ICU‐AW was CIP accompanying CIM, then CIM alone and CIP alone was the least frequent.^420^ While even quadriplegic ICU‐AW patients can recover from CIM in several weeks to several months, CIP has been reported to leave sequelae affecting motor functions lasting for years in some cases.^421^ Polyneuropathy has conventionally been cited as the primary cause of muscle weakness occurring in critically‐ill patients. However, severe sepsis accompanied by multiple organ failure is actually closely related to myopathy.^422^, ^423^ According to the systematic review by Stevens *et al*.,^419^ sepsis and multiple organ failure were risk factors for the onset of ICU‐AW. However, most studies on sepsis and muscle weakness conducted to date have focused mainly on respiratory system muscles, particularly the diaphragm and few studies have looked closely at the muscular strength in the extremities.^423^

The guidelines for the diagnosis of ICU‐AW were published by the American Thoracic Society in 2014.^424^ According to these guidelines, the diagnosis of ICU‐AW should be based on physiological findings (84%, 26/31), electromyogram (EMG) findings (90%, 28/31), and nerve conduction study (NCS) results (84%, 26/31), according to the results of a systematic review of 31 papers selected from the literature. The bedside manual muscle test (MMT) was used to gather physiological findings, and the Medical Research Council (MRC) score,^425^ which comprises numerous items, was also frequently used. A correlation between MMT and MRC score was confirmed for EMG and NCS, and severe muscle weakness was often defined as an MRC score of ≤48/60 total points. Maintaining the patient in an alert state is important for diagnoses based on these physical findings, and accurate judgments cannot be made unless the patient is in a suitable conscious state following reversal of sedation. Diagnosis is particularly inappropriate while patients are in a state of delirium or sepsis‐associated encephalopathy, and careful attention is necessary.

Clinical factors associated with ICU‐AW include sepsis, immobility, hyperglycemia, use of steroid drugs, use of muscle relaxants, among others.^426^ In particular, according to the above guidelines, if the aggregate of severe sepsis patients targeted by the referenced studies (262 patients in total) is taken, the percentage of patients also exhibiting severe muscle weakness was higher than that of other patient groups (504 patients in total; 64% versus 30%, *P *<* *0.001). It has also been pointed out that ICU‐AW incidence increases as the artificial respiration period lengthens.

### Post‐Intensive Care Syndrome

The SCCM hosted a consensus conference focusing on PICS in 2010.^417^ It was decided that the capacity of PICS to affect patients’ (i) motor function, and (ii) cognitive function, as well as cause (iii) psychiatric disorders and (iv) adverse effects on the mental condition of patients’ family members during ICU admission, shortly after discharge, and during the subsequent long‐term period should be widely recognized. The second SCCM consensus meeting was held in 2012, and the agenda topics included more concrete details regarding PICS, such as understanding the condition, preventative measures, risk assessment during treatment, and research promotion.^427^

Factors related to PICS can be broadly divided into four categories. (i) The patient's disease and its severity, (ii) treatment intervention(s), (iii) ICU environmental factors (alarm sounds, light levels), and (iv) patient's mental state (various stressors, economic aspects of the patient's condition, family anxiety). These factors are considered to be complexly intertwined, and each contributes to the onset of PICS. In 2000, Nelson *et al*.^428^ reported that the use of sedatives and muscle relaxants in patients with acute lung injury was associated with depression and the development of post‐traumatic stress disorder (PTSD), and therapeutic factors such as drug therapies, blood transfusions, fluid transfusions, artificial respiration, and blood apheresis may also contribute to the onset of PICS. Aside from treatment, care factors may also be related to the onset of PICS. Specifically, aspirating sputum and changes in posture have been cited. Psychological factors potentially contributing to the onset of PICS include delirium, insomnia, restlessness, and psychological stress, while environmental factors include electronic noises produced by monitors, alarm sounds, and the enclosed ICU environment. One fascinating method of preventing PICS that incorporates a variety of care and psychological factors is the ICU diary. In 2010, Jones *et al*.^429^ reported based on a multicenter prospective study that patients’ family members or attending medical professionals can suppress the onset of PTSD by keeping a diary of ICU inpatients. This report also pointed out that PICS is linked to sepsis, and severe sepsis survivors exhibited greater use of social welfare resources than non‐severe sepsis patients during a one‐year period.^430^

Although there have been several reports regarding ICU‐AW and PICS published in recent years, most involve observational studies, and the evaluation of functional prognosis based on multiple randomized controlled trials (RCTs) has only been conducted with respect to electrical muscle stimulation therapy and rehabilitation programs. For this reason, this guideline presents CQs addressing the validity and efficacy of these two interventions based on meta‐analyses.

Understanding ICU‐AW and PICS, and administering treatment interventions should be done with the aim of enabling ICU patients to return to society, and cooperating with medical personnel not involved in intensive care is also necessary. Both goals are attracting increasing attention as new tasks for those practicing intensive care medicine, and it is important to share the latest knowledge regarding methods of prevention and treatment.

### CQ18‐1 Should electrical muscle stimulation be performed as a method of preventing ICU‐AW?

#### Answer (Recommendation)

We suggest against performing electrical muscle stimulation as an ICU‐AW preventative measure when handling sepsis or ICU patients (2C; rate of agreement: 100%).

#### Rationale

Although artificial respiration period, ICU stay time, and hospitalization period may each increase as a result of ICU‐AW, no consensus has been reached regarding effective therapies for ICU‐AW, and preventative measures are expected. Electrical muscular stimulation induces muscle contractions by percutaneously channeling low‐frequency electrical currents. In some cases, sufficient rehabilitative therapy may not be implemented for patients with chronic heart failure and chronic obstructive pulmonary disease arising from exertional dyspnea, and electrical muscle stimulation, which can be performed even in a resting state is used as an alternative therapy.^431^, ^432^ Although improvements in muscular strength and exertion capacity have been reported as results,^433^ the effectiveness of this intervention in critically‐ill patients or sepsis patients is currently unknown. As such, this CQ examines the capacity of electrical muscle stimulation to prevent the onset of ICU‐AW.

Two single‐center RCTs have reported on the efficacy of electrical muscle stimulation as a measure to prevent ICU‐AW.^434^, ^435^ According to the results of an intent‐to‐treat analysis^436^ of the findings obtained by Routesi *et al*.^434^ and Kho *et al*.,^435^ there were no significant differences in the incidence of ICU‐AW in comparison with the control group. This body of evidence can be said to be inadequate, in consideration of the low number of subjects in the electric muscle stimulation group and the associated risk of bias, in addition to the lack of a high‐quality systematic review or meta‐analysis at this time. Three single‐center RCTs examining whether muscle mass increases as a result of electrical muscle stimulation^437^, ^438^, ^439^ were identified and subjected to a meta‐analysis.^440^ Although subject muscle mass increased significantly, the total number of subjects in the electric muscle stimulation group was low (72 subjects) and the bias risk was high, and accordingly, this evidence may be considered to be of poor quality. No studies analyzing the period of artificial respiration and ICU stay time have been conducted. Although the incidence of ICU‐AW is the most important outcome considered by this CQ, due to the low statistical power of the two studies examined, this body of evidence was rated C (weak).

To perform this intervention, patients must undergo electrical muscle stimulation in the lower limbs for ~1 h each day with time allotted for rest periods. Some pain may occur during this intervention. Although the labor burden placed on nurses, attending physicians and physical therapists in connection with this intervention is not anticipated to be great, medical facilities must possess an electrical stimulation apparatus to perform the intervention, and equipping all facilities for this intervention is considered to be impracticable.

### CQ18‐2 Should early‐stage rehabilitation be implemented to prevent PICS (as well as ICU‐AW)?

#### Answer (Recommendation)

We suggest implementing early‐stage rehabilitation as a PICS preventative measure for sepsis or ICU patients (2C; rate of agreement: 100%).

#### Rationale

PICS, in which the functional prognosis of the body's physical, cognitive, and psychological capacities deteriorate as a result of staying in the ICU, has increasingly been cited in recent years as a concern facing ICU patients, including sepsis patients. The epidemiology, prevention, and treatment of PICS are also gaining greater research attention. Early‐stage rehabilitation interventions are conducted as preventive measures. No RCTs assessing such interventions in sepsis cases only has been conducted to date. However, several RCTs targeting intensive care patients have been completed, and it was determined that this evidence could be extended to sepsis cases as well to assess validity.

We evaluated effect of early‐stage rehabilitation on PICS‐related outcomes used our meta‐analysis (8 studies; Brunmmel *et al*.,^441^ Burtin *et al*.,^442^ Dantas *et al*.,^443^ Denehy *et al*.,^444^ Jones *et al*.,^445^ Kayamabu *et al*.,^446^ Pattanshetty *et al*.,^447^ and Schweickert *et al*.^231^) and 2 meta‐analysis.^436^, ^448^ These results revealed that implementation of early‐stage rehabilitation interventions resulted in significantly improved motor function, 6‐min walk distance (6MWD), and shortened artificial respiration periods. Although no significant differences were observed regarding the incidence of PICS, because intervention subjects’ exhibited significant improvements in MRC score (an ICU‐AW assessment tool), 6MWD, and artificial respiration period, this intervention is expected to be more beneficial than harmful. However, patients targeted by the studies examined were ICU patients rather than sepsis patients, and moreover, the scale of the studies for each PICS‐related outcome was small. As such, in considering the influence of the median/interquartile range to mean/standard deviation conversion applied during the meta‐analyses, the level of this body of evidence cannot be said to be high. In addition, the lack of analysis of side effects also increased the difficulty of the assessment.

To administer this intervention, patients must participate in a rehabilitation program scheduled on a daily basis. This intervention will increase the workloads of nurses, physical therapists, and attending physicians. Administering the intervention with great care and under adequate observation is necessary in serious disease cases, and this intervention should be considered to be highly technical. As such, serious concern is warranted regarding the feasibility of performing this intervention at facilities without sufficient medical personnel or other appropriately‐trained staff.

## CQ19: PEDIATRIC CONSIDERATIONS

### Introduction

The sepsis‐related mortality rate in children is over 15% in patients with severe sepsis cases and even higher in septic shock,^449^, ^450^, ^451^ according to reports published as of 2012. The Surviving Sepsis Campaign Guidelines (SSCG) 2012 edition includes recommendations pertaining to child patients as “Pediatric Considerations”.^29^ However, no content related to pediatric patients was included in the Japanese Society of Intensive Care Medicine (JSICM) guidelines^1^ published the same year. Therefore, a separate and independent chapter on pediatric patients was originally planned for inclusion in the Japanese Clinical Practice Guidelines for Management of Sepsis and Septic Shock 2016 during its creation. The clinical questions (CQs) presented in this chapter were formulated in reference to the aforementioned SSCG 2012^29^ and the consensus statement for the management of pediatric severe sepsis in 2014^452^ (included the American College of Critical Care Medicine/Pediatric Advanced Life Support (ACCM/PALS) algorism^453^), while considering the degree to which they could be supported by the related literature and their importance in the clinical setting.

The definition of pediatric sepsis presented the greatest challenge to the team responsible for this subject matter. A new definition of sepsis (Sepsis‐3) was published in 2016,^5^ characterizing sepsis as “infection accompanied by organ dysfunction.” In essence, the new definition of sepsis is closer to the conventional definition of severe sepsis, and it was decided that the term “severe sepsis” would no longer be used. However, this new definition only applies to adult patients, and to date there has been no movement by any nation towards establishing a definition specific to pediatric patients. Meanwhile, there have been no concrete efforts to accumulate and analyze clinical data from pediatric sepsis patients, making it difficult to propose a new definition of sepsis that also conforms to the Sequential Organ Failure Assessment (SOFA) criteria for adult sepsis patients.^454^ As such, currently, the description of “infection‐induced systemic inflammatory response syndrome (SIRS)” based on the criteria and definition proposed by Goldstein *et al*. in 2005^455^ is designated as sepsis (“‘old’ sepsis”), and the conception of severe sepsis as “infection accompanied by organ dysfunction” is designated as “‘old’ severe sepsis”, and “sepsis” has been newly defined to include “infection accompanied by organ dysfunction” as well, in line with the Sepsis‐3^5^ definition.

However, it should be noted that various important concerns have been identified concerning the criteria proposed by Goldstein *et al*.^455^ First, the following issues have been raised regarding the SIRS diagnostic criteria for pediatric patients:

1. There is no basis for requiring the inclusion of body temperature or white blood cell count.
2. The threshold value for respiratory rate overlaps with the normal range.
3. The results of several recent large‐scale studies^456^, ^457^, ^458^, ^459^ examining normal heart rate and respiratory rate in children are not reflected in the threshold values for respiratory rate and heart rate.

Next, various concerns have also been raised regarding the pediatric evaluation criteria for organ dysfunction, such as the following:

1. There have been no studies evaluating the validity of the respective definitions for each type of organ dysfunction.
2. There is a lack of evidence supporting the threshold value for hypotension used to diagnose septic shock.
3. The evaluation criteria state that severe sepsis may be diagnosed based on single organ dysfunction in Acute respiratory distress syndrome (ARDS) or circulatory failure cases, and although an initial diagnosis of severe sepsis may be made based on the observation of the dysfunction of two or more other organ systems, the basis for this is weak.

In the future, a pediatric version of the definition of sepsis reflecting a confirmed correlation with prognosis based on clinical data will need to be developed.

Pediatric intensive care units (PICU) primarily handle infant and preadolescent patients as well as neonates less than 28 days old. Problems concerning premature births and unborn fetuses, or complications arising during the postnatal period to the transitional period are also the jurisdiction of the neonatal intensive care unit (NICU), and this guideline does not present any CQs related only to neonates. However, the accompanying data analyses and discussions do address in part neonates (regular term/mature fetuses). However, as the definition of the age range considered as “pediatric” differs among papers from different countries or regions, this aspect was not strictly defined. High‐quality scientific evidence focusing only on pediatric cases is given priority, and in unclear cases, consideration is given to ensure consistency with the recommendations pertaining to adult patients. This can be seen in the consensus opinions in the SSCG 2014^29^ or the 2014 consensus statement for the management of pediatric severe sepsis,^452^ and supplementing the findings in children in accordance with the basis of the adult recommendations was considered to be both a scientific and rational approach.

### CQ19‐1 Should sepsis in pediatric patients be defined as infection (including the possibility of infection) complicated by SIRS?

#### Answer (Opinion)

Pediatric sepsis is currently defined as “SIRS arising from infection” according to the criteria and definitions proposed by Goldstein *et al*.^455^ Although “severe sepsis” was defined as “a condition accompanying organ dysfunction” in the definition of Goldstein *et al*., this term has now been replaced with “sepsis,” in line with the terminology revision in the Sepsis‐3^5^ definition. When using this criterion, refer to the proposed changes presented in CQ19‐2 below pertaining to the number of inspirations, hypotension, and creatinine value. However, because the concept of SIRS was excluded from the adult Sepsis‐3 definition,^5^ equating similar expressions, specifically “organ dysfunction accompanying infection,” to “sepsis” should not be dismissed (expert consensus/no evidence; rate of agreement: 100%).

#### Rationale

The pediatric sepsis definition proposed by Goldstein *et al*.^455^ in 2005 (the “Goldstein definition”) was referenced. The diagnostic criteria for SIRS included in the Goldstein definition^455^ are described in Table 2. Table 3 describes the diagnostic criteria for organ dysfunction, which are preconditions for severe sepsis under the same definition. This guideline also allows for the use of the conventional replacement of “severe sepsis” as “sepsis” when using these criteria, in consideration for consistency with the SOFA scoring system.^454^

**Table 2:** Pediatric diagnostic criteria for SIRS (excerpted from Ref. 455)

	Tachycardia (bpm)	Bradycardia (bpm)	Respiratory rate (breaths/min)	WBC count (×1,000/μL)	Systolic blood pressure (mmHg)
0 days–1 week	>180	<100	>50	>34	<59
1 week–1 month	>180	<100	>40	>19.5 or <5	<79
1 month–1 year	>180	<90	>34	>17.5 or <5	<75
2–5 years	>140	–	>22	>15.5 or <6	<74
6–12 years	>130	–	>18	>13.5 or <4.5	<83
13–17 years	>110	–	>14	>11 or <4.5	<90

Systolic blood pressure also draws reference from 460

**Table 3:** Organ dysfunction criteria for the diagnosis of severe sepsis (excerpted from Ref. 455)

Cardiovascular system Despite the infusion of ≥40 mL/kg of fluid for 1 h, presentation of:
Hypotension Use of vascular inotropic drugs to maintain blood pressure Any two of the following conditions: metabolic acidosis, high serum lactate level, oliguria, prolonged capillary refill time (CRT), or central/peripheral temperature discrepancy
Respiratory system PaO_2_/FiO_2_ ratio <300 PaCO_2_ >65 Torr or increase from the standard value by 20 Torr ≥92% SpO_2_ despite maintenance of FiO_2_ > 0.5 Need for mechanical ventilation requiring tracheal intubation, or noninvasive positive pressure ventilation
Nervous system Score of ≤11 on the Glasgow Coma Scale (GCS) Acute changes in state of consciousness (fall of ≥3 on the GCS)
Coagulatory function Platelet count under 80,000/μL or 50% decrease in 3‐day peak platelet count Prothrombin time‐international normalized ratio (PT‐INR) >2
Renal function Two‐fold or higher serum creatinine value compared with the normal upper limit creatinine value applicable to the age group or two‐fold increase from the typical creatinine value
Hepatic function Total bilirubin ≥4 mg/dL Two‐fold or higher alanine aminotransferase (ALT) level compared to the normal upper limit applicable to the age group

Meanwhile, a new definition for adult sepsis (Sepsis‐3) was proposed in 2016.^5^ Under this new definition, sepsis refers to infection (including suspected infection) accompanying organ dysfunction with a SOFA score of two or higher.^454^ However, no pediatric SOFA score system currently exists. The definition of pediatric sepsis is expected to be revised internationally in the future.

### CQ19‐2 What criteria should be used with regard to respiratory rate?

#### Answer (Opinion)

A clear recommendation regarding the threshold value for respiratory rate in the diagnosis of pediatric SIRS cannot be offered at this time. As an example, refer to the criteria proposed by Nakagawa and Shime^461^ (expert consensus/no evidence; rate of agreement: 100%).

#### Rationale

The reference values given in the Goldstein definition regarding the number of inspirations in the diagnosis of SIRS of 18/min for patients aged 6–12 years, and 14/min for patients aged 13–18 years are both lower than the 20/min mentioned in the adult SIRS diagnostic criteria and also overlap with the normal range.^455^ In addition, according to many widely used guidelines such as the pediatric resuscitation guidelines^462^ and the triage standards,^463^ abnormal respiratory rate in infants is defined as ≥60 inspirations per minute. In consideration of these, the standards proposed by Nakagawa & Shime^461^ were created based on the results of Fleming's research^456^ (Table 4). The suitability of the use of this standard should be verified in the future.

**Table 4:** Threshold values for respiratory rate (excerpted from Ref. 461)

0 days–1 week	60
1 week–1 month	60
1 month–1 year	50
2–5 years	30
6–12 years	24
13–18 years	20

Table values are normal upper limit values for respiratory rate proposed by the Guideline Committee.

### CQ19‐3 What criteria should be used with regard to hypotension?

#### Answer (Opinion)

A clear recommendation concerning a threshold value for systolic blood pressure as a diagnostic criterion for pediatric septic shock cannot be offered at this time. As an example, refer to the hypotension criteria used in the SPROUT (Sepsis Prevalence, Outcomes, and Therapies) study^464^ (expert consensus/no evidence; rate of agreement: 100%).

#### Rationale

The diagnostic criteria for severe sepsis under the Goldstein definition are shown in Table 2 of CQ19‐1,^455^ but as the threshold value for systolic blood pressure does not steadily increase with age, an unnatural impression is unavoidable. Therefore, with respect to hypotension criteria for the diagnosis of septic shock, the criteria prepared based on the inclusion criteria of the SPROUT study^464^ have been cited as creating less discomfort than those of the Goldstein definition (Table 5). The validity of the use of these criteria must be confirmed in the future.

**Table 5:** Threshold values for hypotension (excerpted from Ref. 464)

Age range	Hypotension (mmHg)
Up to 1 week	60
1 week–1 month	65
1 month–1 year	70
2–5 years	75
6–12 years	85
13–18 years	90

Instead of Table 2, you can use following formula; 70 + 1.6 × [age] (for patients ≥1 year old).

To reach a septic shock diagnosis under Sepsis‐3, two conditions must be satisfied: (i) vascular inotropic drugs must have been used to maintain a specific mean blood pressure (65 mmHg in adults), and (ii) a serum lactate level of ≥2 mmol/L.^5^ In contrast, to diagnose septic shock under the Goldstein definition, a patient must meet the following criteria despite having received ≥40 mL/kg of fluid during resuscitation within 1 h after hospitalization^455^:

1. Hypotension.
2. Use of vascular inotropic drugs to maintain blood pressure.
3. Any two of the following conditions: metabolic acidosis, high serum lactate level, oliguria, prolonged CRT, or central/peripheral temperature discrepancy.

Therefore, although septic shock can be said to be a relatively broad concept under Goldstein's definition,^455^ under the Sepsis‐3 definition,^5^ severe septic shock is defined in the group of patients presenting with shock facing a particularly high degree of mortality risk (approximately 40% mortality in adults). That is, the Goldstein definition^455^ anticipates an intention to administer early‐stage treatment, while the Sepsis‐3 definition^5^ is considered to isolate a specific group of patients with a relatively high mortality risk. The proportion of pediatric patients satisfying the criteria for septic shock under both the Goldstein definition^455^ and the Sepsis‐3 definition^5^ is currently unknown.

Currently, the Goldstein definition^455^ is used as a standard for pediatric septic shock, and the low blood pressure thresholds to be used in such cases are described in Table 1.

Instead of Table 1, you can use following formula; 70 + 1.6 × [age] (for patients ≥1 year old).

### CQ19‐4 Is establishing reference creatinine values for pediatric use necessary?

#### Answer (Opinion)

We suggest establishing respective reference creatinine values for pediatric patients in different age groups (expert consensus/no evidence; rate of agreement: 100%).

#### Rationale

Measuring creatinine values is indispensable for diagnosing renal dysfunction, but the reference values vary widely depending on the age group of the patient. When assessing organ dysfunction based on SOFA score,^454^ the normal upper limit threshold value for pediatric patients is adjusted to 0 points, and the following four items are multiplied by several numerical values according to the SOFA score^454^ (Table 6). The appropriateness of this method must be verified in the future. Regarding reference values, the PELOD (Performance of the Pediatric Logistic Organ Dysfunction)‐2 (Table 7(a))^465^ scoring system is widely used in international clinical research as an index of pediatric organ dysfunction, and do not necessarily match with the age‐specific reference values proposed in Japan (Table 7).^466^ Because the serum creatinine value depends on the muscle mass, particularly in post‐pubescent patients, large gaps can arise between different genders and ethnicities, and may also result from differences in measuring methods (e.g., Jaffe method, enzyme method, etc.), and validation will be necessary here as well.

**Table 6:** Renal SOFA scoring criteria for pediatric patients

SOFA score	0	1	2	3	4
Kidney	<Cr_0_ (standard value by age group)	1–1.6 × Cr_0_	1.7–2.8 × Cr_0_	2.8–4.1 × Cr_0_	≥4.2 × Cr_0_

Scores are calculated based on the respective standard values for each age group.

**Table 7:** Standard values by age (Cr_0_)

(a) Upper limit threshold value for PELOD‐2 (Ref. 465) renal disorder score of 0 points (unit: converted to mg/dL)	
0 to <1 month	0.8 mg/dL
1–11 months	0.3 mg/dL
1–2 years	0.4 mg/dL
2–5 years	0.6 mg/dL
5–12 years	0.7 mg/dL
≥12 years	1.0 mg/dL

(b) Normal creatinine values by age as obtained from a Japanese multicenter study (Ref. 466)						
Age	2.5th percentile (mg/dL)		50th percentile (mg/dL)		97.5th percentile (mg/dL)	
	M	F	M	F	M	F
3–5 months	0.14		0.20		0.26	
6–8 months	0.14		0.22		0.31	
9–11 months	0.14		0.22		0.34	
1 year	0.16		0.23		0.32	
2 years	0.17		0.24		0.37	
3 years	0.21		0.27		0.37	
4 years	0.20		0.30		0.40	
5 years	0.25		0.34		0.45	
6 years	0.25		0.34		0.48	
7 years	0.28		0.37		0.49	
8 years	0.29		0.40		0.53	
9 years	0.34		0.41		0.51	
10 years	0.30		0.41		0.57	
11 years	0.35		0.45		0.58	
12 years	0.40	0.40	0.53	0.52	0.61	0.66
13 years	0.42	0.41	0.59	0.53	0.80	0.69
14 years	0.54	0.46	0.65	0.58	0.96	0.71
15 years	0.48	0.47	0.68	0.56	0.93	0.72
16 years	0.62	0.51	0.73	0.59	0.96	0.74

### CQ 19‐5 Should a pediatric blood culture bottle be used for pediatric patients?

#### Answer (Opinion)

We suggest the use of pediatric blood culture bottles for pediatric patients (until approximately school‐age; expert consensus/no evidence). We also suggest the use of adult bottles even in pediatric patients if their physique is similar to an adult's and the patient can safely sustain blood collection (approximately ≥36 kg; expert consent/no evidence; rate of agreement: 100%).

#### Rationale

Blood culture testing is an essential technique for the optimization of antibiotic therapy in the treatment of infections/sepsis. In adults, typically as much blood is collected as possible to improve test accuracy. However, when handling pediatric patients, the collection of large volumes of blood is more difficult due to issues related to circulating blood volume, and collection methods similar to those employed in adult patients are generally not used.^467^ Blood culture test procedures suitable for children, including the use of pediatric blood culture bottles, are therefore necessary.

No randomized controlled trials (RCTs) conforming to the Patient, Intervention, Comparison, Outcome (PICO) process could be found. In observational studies, the comparison of aliquots (up to 5 mL) of blood from both adult and pediatric bottles revealed a high rate of positivity in the pediatric bottles and a short time to positive test results.^468^ In essence, this suggests that test results are more likely to be positive when a pediatric bottle is used even if the volume of blood collected is smaller. The target blood sample volume for neonates is 1–2, 2–3 mL for infants, 3–5 mL for infants/school‐age children, and 10–20 mL for pubescent children.^469^ The use of pediatric bottles is generally desirable with pediatric patients. The use of adult bottles is appropriate with older children of school‐age (approximately ≥36 kg) who are able to provide a sufficient volume of blood.

Because the positivity rate for blood volumes lower than 1 mL is low even when a pediatric bottle is used,^467^ pediatric bottles should contain at least 1 mL of blood. As the same amount of blood is collected, the frequency of adverse effects is not likely to increase. Therefore, the potential benefits likely outweigh the potential risks.

### CQ19‐6 How should circulatory inotropes be used to address septic shock in pediatric patients?

#### Answer (Opinion)

Adrenaline is the first‐line vasopressor drug for use in treating septic shock in pediatric patients (expert consensus/quality of evidence: “C”).

Noradrenaline should not be used as the first‐line treatment drug in pediatric patients with septic shock (expert consensus/no evidence).

Vasopressin should not be used to treat vasodilatory septic shock in pediatric patients (expert consensus/quality of evidence: “C”).

Dobutamine or milrinone may be used to treat pediatric septic shock as appropriate given the patient's condition (expert consensus/no evidence; rate of agreement: 100%).

#### Rationale

As with adults, the infusion of appropriate volumes of fluid often does not improve the hemodynamics of pediatric septic shock patients, and the use of circulatory inotropes often becomes necessary. Although the consensus statement for the management of pediatric patients with severe sepsis mentions that, “positive inotropes/vasoconstrictors should be administered as soon as possible to pediatric patients who are unresponsive to transfusion loading,” no specific drugs were recommended.^452^

SSCG 2012 also does not offer strong recommendations regarding the use of any specific circulatory inotrope in children.^23^ Noradrenaline is recommended for use in adults, but there have been no RCTs upon which to validate its use in children. The selection of circulatory inotropes for use in children presenting with septic shock is a routinely encountered issue in practice, and it is important to maintain current knowledge about which drugs are effective under different circumstances.

One RCT^470^ reported that adrenaline use in pediatric patients with sepsis was associated with a lower mortality rate in comparison to dopamine^470^ and recommended adrenaline as a first‐line drug therapy. However, this was a single center study with 120 participants, and it is necessary to recognize that the design of this study weakens it as an evidentiary basis for the use of adrenaline as a first‐line drug.

While there have been no RCTs conducted to date comparing noradrenaline with other drugs or placebo, noradrenaline may also be considered for first‐line use in patients exhibiting high cardiac output and peripheral vascular dilatation. Another RCT investigating children presenting with vasodilatory shock reported that low‐dose vasopressin temporarily increased the blood pressure compared with placebo, but worsened survival and prognosis.^471^ It is important to note, however, that this study did not focus only on sepsis patients. In the management of pediatric patients with septic shock, vasodilators may be considered in cases characterized by peripheral vasoconstriction and blood pressure is maintained, but there is currently no basis for recommending the use of milrinone. Moreover, there have been no RCTs conducted to date comparing dobutamine with other drugs or placebo.

In another study^470^ comparing adrenaline and dopamine, adrenaline was associated with a lower rate of mortality than dopamine. Potential risks include a tendency for dopamine to exacerbate inflammation, and adrenaline was linked to hyperglycemia and persistent hyperlactatemia. Based on these observations, the potential benefits of adrenaline use are believed to outweigh the risks. According to the results of a comparison of vasopressin and placebo,^471^ vasopressin was linked to a higher mortality rate and incidence of adverse events, and the risks likely exceed the potential ‐benefits.

### CQ19‐7 Should CRT be used as a circulatory management indicator in pediatric sepsis cases?

#### Answer (Opinion)

CRT should be used in conjunction with other hemodynamic indicators to monitor the state of circulatory management in pediatric sepsis patients (expert consensus/no evidence; rate of agreement: 100%).

#### Rationale

According to SSCG 2012,^29^ when performing initial resuscitation, ideal conditions include a CRT within 2‐s, normal blood pressure for the age group, normal heart rate, no difference in between the central and peripheral pulses, warmness of the extremities, 1 mL/kg/hr urine volume, normal consciousness, central venous oxygen saturation (ScvO_2_) of 70% or higher, and a cardiac index of 3.3–6.0 L/min/m^2^. However, the measurement of ScvO_2_ requires the insertion of a central venous line, and accurately measuring the cardiac index in children can be difficult. A CRT of ≤2 s in children admitted to the PICU has been reported to be correlated with an ScvO_2_ ≥70%.^472^ A meta‐analysis also found that abnormal CRT was linked to a higher risk of mortality.^473^

It is important to verify the significance of CRT as a noninvasive and continuous circulatory management index that can easily be used by clinicians in the initial diagnosis of pediatric patients with sepsis, particularly in Japan, which has underdeveloped intensive care treatment systems.

No RCTs conforming to the PICO process could be found. CRT is an index of circulatory management that can be evaluated noninvasively and repeatedly over time. Correlations between CRT and ScvO_2_ as well as between abnormal CRT and mortality have also been reported,^473^ and the use of CRT as an indicator of hemodynamic status is believed to offer substantial benefits to patients.

In contrast, the use of CRT alone to gauge the state of circulatory management may lead to excessive treatment intervention. CRT is also influenced by a variety of factors such as measurement site, compression time, temperature, etc.,^474^ and it must be recognized that inconsistencies in the method of evaluation may lead to erroneous interpretation.^475^ However, there are no RCTs conforming to the PICO process, and therefore the benefit‐risk balance is currently unknown but is believed to differ depending on the unique circumstances of individual patients.

### CQ19‐8 Should ScvO_2_, or serum lactate value be used as a circulatory management indicator in pediatric sepsis cases?

#### Answer (Opinion)

Both ScvO_2_ and serum lactate values may be used as indicators of circulatory management in pediatric sepsis cases. However, use of comprehensive circulatory evaluation together with other hemodynamic indicators is required (expert consensus/no evidence; rate of agreement: 100%).

#### Rationale

ScvO_2_ and serum lactate value are both indicators of the tissue oxygen supply/demand balance, and numerous studies have been conducted to investigate the positive and negative aspects of their use as indicators of hemodynamic status in patients with sepsis. ScvO_2_, in addition to central venous pressure, mean arterial pressure, and urine volume, have been recommended as hemodynamic indicators when performing initial resuscitation in adult sepsis patients.^29^ However, the clinical utility of quantitative protocols for circulatory management applying these metrics has been called into question in recent years. In children, the measurement of ScvO_2_ has been recommended in conjunction with the use of indices measurable through noninvasive methods, such as vital signs, peripheral circulation, and urine volume.^29^, ^453^ However, the invasiveness and costs accompanying this technique warrant its reconsideration.

SSCG 2012 recommends the use of normalization of serum lactate values as an indicator of initial resuscitation in adult sepsis patients presenting with hyperlactatemia.^29^ Also, according to Sepsis‐3,^5^ elevated serum lactate level is used to define septic shock. The utility of the absolute or chronological lactate value in predicting mortality or organ dysfunction in pediatric patients with severe sepsis has also been reported based on an observational study.^452^ An evaluation of the utility of these metrics in sepsis cases as well must be conducted in the future.

One RCT^476^ conforming to the PICO process was found. Application of the American College of Critical Care Medicine (ACCM)/Pediatric Advanced Life Support (PALS) guideline,^453^ which calls for the continuous monitoring of ScvO_2_, was linked to a decrease in 28‐day mortality rate and a lower incidence of novel organ disorders in comparison to cases where ACCM/PALS was not applied. However, this was an unblended overseas study, and it must also be recognized that baseline characteristics of the two study groups were different.

No RCTs investigating the use of serum lactate value in managing circulation could be found.

ScvO_2_ and serum lactate value are both indicators of the tissue oxygen supply/demand balance, and implementing circulatory management aiming for normalization of these indices is considered valid. However, the placement of a central venous catheter or an arterial catheter is necessary. While both are useful in the management of pediatric patients with sepsis, adverse events such as mechanical complications during placement, catheter‐related bloodstream infection (CRBSI) following placement, thrombosis, or peripheral blood flow disorders may occur. Also, sedation, as well as tracheal intubation, may be necessary when placing the central venous catheter in pediatric patients, and the workload of attending medical staff can also be expected to increase. However, the frequency of complications will vary depending on the physician/technician, the facility environment, and individual patient characteristics, and clinical benefits obtained will also vary depending on the patient's condition. Therefore, the benefit‐risk balance is expected to vary by case.

### CQ19‐9 What should the target hemoglobin (Hgb) value be in pediatric sepsis cases?

#### Answer (Opinion)

Hgb >7 g/dL may be used as a target value as appropriate based on the patient's condition after shock and hypoxemia have been corrected, although each patient's course will be different. (expert consensus/no evidence; rate of agreement: 100%).

#### Rationale

Anemia develops readily in sepsis as a result of multiple concurrent events, such as bleeding tendency due to disseminated intravascular coagulation and frequent blood sampling for diagnostic testing. Correcting anemia is important to normalize the supply of oxygen to tissues and to ease the burden on the heart. Contrastively, cardiac overflow due to excessive blood transfusion may worsen respiratory condition and hemodynamics. Performing blood sampling and transfusions only when necessary is also advantageous from the perspective of infection control and prevention.

The Transfusion Requirements in the Pediatric Intensive Care Unit (TRIPICU) study^477^ investigated target Hgb values for pediatric intensive care patients. Slightly less than 40% of the patients targeted were sepsis patients, and the study compared two groups that underwent “blood transfusion with Hgb <7 g/dL; target range: 8.5–9.5 g/dL” and “blood transfusion with Hgb <9.5 g/dL; target range: 11–12 g/dL”. As a result, the average Hgb values were 8.7 ± 0.4 g/dL versus 10.8 ± 0.5 g/dL, respectively, and the mortality rate, incidence of multiple organ failure, and hospitalization time did not differ between the two groups. When determining the target Hgb levels in pediatric sepsis patients, a literature review of relevant studies was considered to be an important clinical task.

No RCTs conforming to the PICO process could be found. By establishing Hgb >7 g/dL as the target value in septic shock cases or patients presenting with hypoxemia despite stable hemodynamics, blood transfusion frequency can be reduced, medical resources can be utilized more effectively, medical costs can be reduced, and the frequency of complications associated with blood transfusion may be lowered.

In contrast, gaining a sufficient grasp of the patient's circulatory condition is necessary if Hgb >7 g/dL is to be established as the target Hgb value after hemodynamics have stabilized. Also, when maintaining a lower Hgb value, responses to acute hemorrhaging may narrow the scope of options for managing shock. As no RCTs conforming to the PICO process were found, the benefit‐risk balance is currently unclear. However, this balance is believed to differ depending on the condition and circumstances of individual patients.

### CQ19‐10 Should steroids be administered in pediatric sepsis cases?

#### Answer (Recommendation)

We suggest against the administration of steroids as a standard treatment in pediatric septic shock cases (2D; rate of agreement: 100%).

#### Rationale

Administering steroid therapy in sepsis cases had long been considered to be a vital clinical task. However, according to SSCG 2012,^29^ the routine use in adult septic shock patients is not recommended. The use of steroids in pediatric patients with severe sepsis has not been linked with survival prognosis in the overseas or Japanese literature.^451^, ^478^

In contrast, steroid therapy is recommended in the pediatric section of SSCG 2012.^29^ This recommendation is based on the observation that pediatric septic shock cases in Europe and the United States are often caused by meningococcal bacteria, and the effect of the rapid development of acute adrenal dysfunction and the high rate of mortality in the pediatric population must also be taken into account. Japan has a low incidence of this condition, and its treatment warrants a different approach than in the western nations.^479^ In addition, dengue fever patients with shock have been disproportionately represented in other clinical studies assessing the significance of steroids in the treatment of pediatric sepsis, which is far from the situation in Japan. As such, further investigations not influenced by the results of studies on dengue heat shock are believed to be necessary. Although many clinical studies have assessed neurological prognosis by administering multiple steroid drugs from the start of meningitis treatment, this CQ only addresses the use of steroids to support recovery from shock.

Administering steroids will not lead to a reduced risk of mortality or shortened shock recovery period in pediatric septic shock patients who are unresponsive to fluid resuscitation and dependent on circulatory inotropes. However, the incidences of complications such as bleeding and secondary infections do not increase. The evidence upon which this recommendation is based was limited to a single RCT conducted in a developing country.^480^ The most important outcome considered in this CQ is mortality rate, and shock recovery rate and the incidence of complications are somewhat less important. As the evidence pertaining to mortality rate is rated “D” (very weak), the overall evidence regarding this outcome is also rated “D” (very weak).

Decreased mortality rate and increased shock recovery rate/faster shock recovery are considered to be benefits of this intervention. However, no significant differences with respect to mortality rate and shock recovery period were observed in the only RCT adopted for this CQ. Meanwhile, potential harm associated with this intervention include an increased incidence of complications (e.g., bleeding and secondary infection), but no significant difference was observed. Accordingly, the benefit‐risk balance for this intervention is currently suspected to be unfavorable, or uncertain.

### CQ19‐11 Should blood apheresis be performed as a treatment for septic shock in pediatric patients?

#### Answer (Opinion)

No recommendation regarding the use of blood apheresis as a treatment in pediatric septic shock cases can be offered at this time due to insufficient evidence (expert consensus/no evidence; rate of agreement: 100%).

#### Rationale

SSCG 2012^29^ does not address the use of blood apheresis to treat septic shock in adult or pediatric patients. However, as several new RCTs^274^, ^275^, ^481^ to investigate the utility of blood apheresis in the treatment of sepsis in adult patients have been announced successively in Europe and the United States, assessing the significance of this intervention for pediatric patients is believed to be valuable.

Only a single study^482^ conforming to the PICO process was found. However, this study enrolled few subjects and was terminated early; thus, the body of evidence for this CQ is currently considered to be inadequate. Accordingly, no recommendation could be offered, and an expert consensus is presented instead.

Patient condition may be improved as a result of performing blood apheresis to aid in regulating immunological response through the removal of inflammatory cytokines and mediators. However, no meta‐analysis or RCT has been conducted to date assessing renal replacement therapy and direct hemoperfusion with polymyxin B‐immobilized fiber in children, and a study on plasma filtration^482^ found no improvement in mortality rate even after adjusting for disease severity. Adverse events associated with blood apheresis include bleeding while securing venous access, drops in blood pressure after initiation of blood apheresis, electrolyte anomalies, hypothermia, bleeding due to anticoagulant use, among others.

Mechanical complications occurring while securing blood access are expected to be particularly high in pediatric patients. When handling pediatric patients, the experience level of the attending medical personnel can affect the frequency of adverse events. A decision to perform blood apheresis may greatly increase the workload of medical personnel, and this is considered to have a major impact. Although the evidence obtained from the literature search was inadequate to form a basis for any conclusion, the severity of the potential risks associated with this intervention must be considered fully.

### CQ19‐12 Should immunoglobulin therapy be administered in pediatric sepsis cases?

#### Answer (Opinion)

We recommend against the administration of immunoglobulin therapy as a standard treatment in pediatric sepsis cases (expert consensus/no evidence; rate of agreement: 94.7%).

#### Rationale

The use of immunoglobulin therapy to address severe infections is covered under the Japanese National Health Insurance program and is in wide use despite unclear evidence regarding its capacity to improve clinical prognosis. Meanwhile, although numerous studies have been conducted overseas to assess a technique known as immunomodulation, which involves administering large doses of immunoglobulin preparations, the results of these studies have not been consistent. Also, with the exception of neonates, high‐quality RCTs targeting pediatric patients are currently lacking. Immunoglobulin preparations are expensive, and further clarification of their clinical efficacy will have great significance.

The adult patient‐oriented sections of SSCG 2012^29^ do not support the administration of intravenous immunoglobulin (IVIG) preparations, and while the accompanying commentary touches on the significance of the International Neonatal Immunotherapy Study (INIS) trial,^483^ a multicenter RCT that verified the efficacy of IVIG in neonates, SSCG 2012 does not contain a pediatric patient‐oriented chapter on IVIG. The INIS trial is the largest multicenter RCT on this subject, and the fact that IVIG's effectiveness was not observed in adult or pediatric patients cannot be ignored. The fact that IVIG was found to be ineffective despite how many subjects were premature infants with a history of hypogammaglobulinemia is particularly significant.

No studies conforming to the PICO process were found. According to the results of a systematic review or meta‐analysis^107^ limited to neonates only, no improvement in mortality rate as a result of polyclonal IVIG use was observed, and in the INIS trial^483^ (*n* = 3,493) as well, which was also adopted for this systematic review, no significant differences between the intervention and control groups were observed with respect to mortality rate and incidence of severe sequelae. Although adverse effects such as hyperviscosity syndrome and acute renal failure have been associated with this intervention, these were not among the frequently occurring adverse events reported for the intervention group in the INIS trial^483^ (intervention group: 12/1,759; control group: 10/1,734). The INIS trial^483^ was not adopted as a direct basis for the recommendation decision for this CQ because the majority of subjects were premature infants and the targets differed. It can, however, be inferred from the fact that the administration of IVIG preparations to adult sepsis patients leads to neither benefits nor risks that the same is likely true for pediatric patients.

### CQ19‐13 Should strict glycemic management be implemented for pediatric sepsis patients?

#### Answer (Recommendation)

We do recommend against applying strict glycemic management in pediatric sepsis cases (1B; rate of agreement: 100%).

#### Rationale

As with adult patients, numerous reports have suggested relationships between hyperglycemia and high mortality rate, as well as hyperglycemia and hospitalization period in pediatric patients as well. Although not limited to sepsis cases, several successively announced large‐scale RCTs^484^, ^485^, ^486^, ^487^ have assessed the significance of implementing strict glycemic management in critically‐ill children. Thus, investigating the importance of this type of intervention in patients with severe sepsis is considered to be an important task.

While four RCTs^484^, ^485^, ^486^, ^487^ targeting critically‐ill pediatric patients were adopted for a meta‐analysis of strict glycemic management (Srinivasan and Agus^488^), all four studies involved intensive care unit (ICU) patients other than sepsis patients, and no subgroup analysis limited to sepsis patients was reported.

While the incidence of complication by secondary infection declines as a result of implementing strict glycemic management in pediatric sepsis or PICU patients, hypoglycemia occurs more frequently, and no significant improvement in mortality rate can be expected. Hypoglycemia is a serious complication that can lead to severe neurological sequelae over the longer term in children, particularly infants. Therefore, it was concluded that the potential risks associated with this intervention likely outweigh the benefits, and so it is not recommended, regardless of cost or feasibility.

Although lowered mortality is an expected benefit of this intervention, no significant difference was observed between the intervention and control groups, and the corresponding odds ratio was 0.79 (95% confidence interval [CI]: 0.55–1.15). Meanwhile, the decline in complications by secondary infections is also considered to be a clinical benefit, but one of less overall importance. The corresponding odds ratio was 0.76 (95% CI: 0.59–0.99), and a statistically significant decrease was observed in the intervention group. While increased incidence of hypoglycemia is a harm caused by this intervention, the odds ratio for this adverse effect was 6.14 (95% CI: 2.74–13.78) and was significantly higher in the intervention group. Given that severe hypoglycemia is a serious complication that gives rise to concerns regarding its long‐term impact on neurological development, it was concluded that the potential harms associated with this intervention likely outweigh its potential benefits.

### CQ19‐14 Is the ACCM‐PALS algorithm useful for managing septic shock in pediatric patients?

#### Answer (Opinion)

The ACCM‐PALS initial treatment algorithm may be used as necessary in consideration of patient condition and needs of the clinical environment (expert consensus/no evidence; rate of agreement: 100%).

#### Rationale

Performing resuscitative measures without delay and facilitating recovery from the shock state as soon as possible is desirable when treating pediatric septic shock. As such, the application of the ACCM‐PALS algorithm for pediatric septic shock has been globally adopted, and use of its translated version has entered the mainstream in Japan.^29^ At the same time, it is also important to carefully consider the reliability and validity of other algorithms and their translations and to confirm the utility of existing algorithms.

No RCTs applicable to the subject of this CQ could be found during a literature search of the PubMed database. Although no systematic reviews or RCTs evaluating the validity and utility of the ACCM‐PALS algorithm itself currently exist, several observational studies were found.^489^, ^490^, ^491^ However, it was determined that summarizing these observational studies (conducting a meta‐analysis) would be difficult due to the low quality study design, variation in outcome indicators, and the possibility for high heterogeneity, and no repeat systematic review and meta‐analysis was conducted during the formulation of this guideline. In line with the above, the supporting evidence for this CQ is currently insufficient, and no recommendation can be presented at this time. However, as the expert consensus reached by the Guideline Creation Committee, it was determined that the ACCM‐PALS initial treatment algorithm might be used as necessary in consideration of patient condition and needs of the clinical environment during the treatment of pediatric sepsis. The usefulness and validity of the algorithm itself will need to be verified in the future.

By following this algorithm, various pediatric sepsis treatments can be administered as appropriate and without omission. Contrastively, by adhering to the algorithm, excessive treatments may be offered while treatments not covered by the algorithm may be ignored or their implementation delayed. In addition, some increase in workload can be expected to accompany the work of monitoring and confirming adherence to the algorithm, but this additional burden is considered to be minor. No RCTs conforming to the PICO process were found and as such the benefit‐risk balance for this technique is currently unclear, and is believed to vary depending on patient condition. Because the ACCM‐PALS algorithm is freely accessible, no additional medical costs will be incurred, and the drugs and medical devices covered by the algorithm are available in many intensive care units.

### CQ19‐15 Should the intraosseous route be used temporarily for the administration of fluid resuscitation and circulatory inotropes when treating septic shock in pediatric patients?

#### Answer (Opinion)

The intraosseous route may be used as a temporary route of administration for fluid resuscitation and circulatory inotropes in pediatric septic shock cases, in consideration of patient condition and needs of the clinical environment (expert consensus/no evidence; rate of agreement: 100%).

#### Rationale

The use of the intraosseous route as a temporary delivery route for the administration of fluids and circulatory inotropes is well‐recognized as a pediatric resuscitative technique. However, the use of the intraosseous route accelerates the start of initial resuscitation and may influence outcomes even in pediatric patients with septic shock requiring rapid infusion and the use of circulatory inotropes, and as such the clinical utility of this technique is worth considering.

No RCTs examining the utility of the intraosseous route in pediatric sepsis cases have been conducted to date, but according to the results of one RCT,^492^ the intraosseous route is as useful as the peripheral venous route in pediatric patients with severe dehydration. In this RCT, all bone marrow needles were placed within 5 min (100%), while the venous route was successfully secured 67% of the time. In addition, successfully securing a venous route required much more time in comparison to the time needed when using bone marrow needles (venous route: 129 ± 13 s, 95% CI: 103–156 s versus intraosseous route: 67 ± 7 s, 95% CI: 55–80 s).

Patients requiring resuscitation treatment due to shock or similar conditions exhibit collapsed peripheral blood vessels and securing a venous delivery route frequently presents difficulties. At the same time, resuscitation treatments such as fluid transfusion and drug therapy are also impeded as they often cannot be initiated without access to a venous delivery route. In situations such as these, the intraosseous route can allow for rapid transfusion and drug delivery and is believed to have a high potential for being beneficial to patients. The use of the intraosseous route is also believed to cause little additional increase in workload in comparison to central venous puncture in cases where securing peripheral venous access during resuscitation is difficult. However, careful attention should be paid to the potential for complications when using bone marrow needles, such as malpositioning, hemorrhage, osteomyelitis, compartment syndrome, fat embolism, and tibial fractures.

Although there is currently insufficient evidence to support a recommendation, based on the available evidence pertaining to use of the intraosseous route in pediatric patients with severe dehydration in addition to the accepted understanding of the difficulty in securing peripheral and central venous access in such patients compared to adults, it was determined by expert opinion that both the intraosseous route and the peripheral venous access may be used to facilitate initial fluid resuscitation and administration of circulatory inotropes as treatments in pediatric patients with sepsis.

## FUNDING

These guidelines were prepared with financial support from the Japan Society of Intensive Care Medicine and the Japanese Association for Acute Medicine.

## DISCLOSURE

All committee members and working group members submitted disclosure forms of financial and academic conflict of interest (COI) prior to being requested to participate in individual activities. All COI were collected according to the guideline by Japanese Society of Intensive Care Medicine and the Japanese Association for Acute Medicine. Detailed information of COI and the roles in creating this clinical guideline are summarized in Additional file.

## ABBREVIATION

6MWD; 6‐minute walk distance, ACCM; American College of Critical Care Medicine, ACTH; adrenocorticotropic hormone, ADQI; the Acute Dialysis Quality Initiative, ADRENAL; ADjunctive coRticosteroid trEatment iN criticAlly iLl patients with septic shock, AKI; Acute Kidney Injury, AKIN; the Acute Kidney Injury Network, ALT; alanine aminotransferase, AMSTAR; A Measurement Tool to Assess Systematic Reviews, ANP; atrial natriuretic peptide, ANZICS; Australian and New Zealand Intensive Care Society, APACHE; Acute Physiology and Chronic Health Evaluation, ARDS; Acute respiratory distress syndrome, ARDSGL; ARDS Clinical Practice Guidelines 2016, ARISE; Australasian Resuscitation in Sepsis Evaluation, AUA; American Urological Association, BMI; body mass index, BNP; brain natriuretic peptide, CI; confidence interval, CIM; critical illness myopathy, CIN; contrast‐induced nephropathy, CIP; critical illness polyneuropathy, CIRCI; critical illness‐related corticosteroid insufficiency, CNP; C‐type natriuretic peptide, COI; conflicts of interest, CORTICUS; Corticosteroid Therapy of Septic Shock, CQ; clinical question, CRBSI; catheter‐related bloodstream infection, CRISTAL; Colloids Versus Crystalloids for the Resuscitation of the Critically Ill, CRP; c‐reactive protein level, CRRT; continuous renal replacement therapy, CRT; capillary refill time, CT; computed tomography, CTV; computed tomography venography, CVP; central venous pressure, DIC; disseminated intravascular coagulation, DPC; diagnosis procedure combination, DVT; deep vein thrombosis, EGDT; early goal‐directed therapy, EMG; electromyogram, EPaNIC; Early versus Late Parenteral Nutrition in Critically Ill Adults, EUPHRATES; Evaluating the Use of Polymyxin B Hemoperfusion in a Randomized controlled trial of Adults Treated for Endotoxemia and Septic shock, FACE; Fever and Antipyretic in Critically ill patients Evaluation, FLAIR; Fluid attenuation inversion recovery, GCS; glasgow coma scale, GDT; goal‐directed therapy, GRADE; Grading of Recommendations Assessment, Development and Evaluation, HC; hydrocortisone, Hgb; hemoglobin, HIT; heparin‐induced thrombocytopenia, HYPRESS; Hydrocortisone for Prevention of Septic Shock, ICU; intensive care unit, ICU‐AW; Intensive Care Unit‐Acquired Weakness, IDSA; Infectious Diseases Society of America, IL; interleukin, INIS; International Neonatal Immunotherapy Study, ISTH; International Society on Thrombosis and Hemostasis, IVAC; Infection‐related Ventilator‐Associated Complication, IVIG; Intravenous immunoglobulin, JAAM; Japanese Association for Acute Medicine, J‐PAD; Japanese guidelines for the management of Pain, Agitation, and Delirium in intensive care unit, JSICM; Japanese Society of Intensive Care Medicine, J‐SSCG 2016; The Japanese Clinical Practice Guidelines for Management of Sepsis and Septic Shock 2016, KDIGO; the Kidney Disease: Improving Global Outcomes, KUB; kidney, ureter, and bladder simple X‐ray image, LeoPARDS; Levosimendan for the Prevention of Acute oRgan Dysfunction in Sepsis, MD; mean difference, MIC; minimum inhibitory concentration, Minds; Medical Information Network Distribution Service, ML; mailing list, MMT; manual muscle test, MPSL; methylprednisolone, MRC; Medical Research Council, MRCP; magnetic resonance cholangiopancreatography, MRI; magnetic resonance imaging, NCS; nerve conduction study, NHI; National Health Insurance, NICE‐SUGAR; Normoglycemia in Intensive Care Evaluation and Survival Using Glucose Algorithm Regulation, NICU; neonatal intensive care unit, NMA; network meta‐analysis, NSAIDs; nonsteroidal anti‐inflammatory drugs, PAD guidelines; Clinical practice guidelines for the management of pain, agitation, and delirium in adult patients in the intensive care unit, PALS; Pediatric Advanced Life Support, PAWP; pulmonary artery wedge pressure, PCT; procalcitonin, PE; pulmonary embolism, PEEP; positive end‐expiratory pressure, PELOD; Performance of the Pediatric Logistic Organ Dysfunction, PICO; Patient, Intervention, Comparison, Outcome, PICS; Post Intensive Care Syndrome, PICU; Pediatric intensive care unit, PLR; Passive Leg Raising, PMX‐DHP; Polymyxin B‐immobilized fiber column direct hemoperfusion, POCT; point‐of‐care‐testing, ProCESS; Protocolized Care for Early Septic Shock, ProMISe; Protocolised Management in Sepsis, P‐SEP; presepsin, PT‐INR; Prothrombin time‐international normalized ratio, PTSD; post‐traumatic stress disorder, QoE; quality of evidence, qSOFA; quick SOFA, RBC; red blood cell, RCT; randomized controlled trial, RIFLE; the Risk, Injury, Failure, Loss, End‐Stage Kidney Disease, ROC; receiver operating characteristic, RR; relative risk, RR; risk ratio, RRT; renal replacement therapy, SAFE; Saline Versus Albumin Fluid Evaluation, SCCM; the Society of Critical Care Medicine, sCre; serum creatinine, ScvO2; central venous oxygen saturation, Sepsis‐3; the Third International Consensus Definitions for Sepsis and Septic Shock, SIMD; sepsis‐induced myocardial dysfunction, SIRS; systemic inflammatory response syndrome, SIS; Surgical Infection Society, SOFA; the Sequential Organ Failure Assessment, SOFA; Sequential Organ Failure Assessment, SPROUT; Sepsis Prevalence, Outcomes, and Therapies, SSCG; Surviving Sepsis Campaign Guidelines, SVV; stroke volume variation, TRALI; transfusion‐related acute lung injury, TRIPICU; Transfusion Requirements in the Pediatric Intensive Care Unit, USD; United States Dollar, VALI; ventilator‐associated lung injuries, VAP; ventilator‐associated pneumonia, VFD; ventilator‐free days, VTE; prevention of venous thromboembolism.

## Supporting information


**Appendix.** Committee for the Japanese Clinical Practice Guidelines for the Management of Sepsis and Septic Shock 2016 ‐ Members' names, workplaces, conflicts of interest (COI), and roles.

**Availability of data and materials.** Supplementary materials and files associated with the guidelines can be found at www.jsicm.org/pdf/supplementary_appendix.pdf.
