# Supplementary material for: The Japanese Clinical Practice Guidelines for Management of Sepsis and Septic Shock 2016 (J‐SSCG 2016)
Source: Acute Med Surg. 2018 Feb 5;5(1):3–89. doi: 10.1002/ams2.322 (PMC5797842; doi:10.1002/ams2.322)
Supplement: Supplementary file 2 — Availability of data and materials. Supplementary materials and files associated with the guidelines can be found at www.jsicm.org/pdf/supplementary_appendix.pdf. [file AMS2-5-3-s002.docx]

**Availability of data and materials**

Additional file 1 contains tables disclosing intellectual and financial conflicts of interest and roles for each person who participated in the creation of this guideline. Supplementary materials and files associated with the guidelines can be found at www.jsicm.org/pdf/supplementary_appendix.pdf.
